# Pattern of predictive features of continued cannabis use in patients with recent-onset psychosis and clinical high-risk for psychosis

**DOI:** 10.1038/s41537-022-00218-y

**Published:** 2022-03-09

**Authors:** Nora Penzel, Rachele Sanfelici, Linda A. Antonucci, Linda T. Betz, Dominic Dwyer, Anne Ruef, Kang Ik K. Cho, Paul Cumming, Oliver Pogarell, Oliver Howes, Peter Falkai, Rachel Upthegrove, Stefan Borgwardt, Paolo Brambilla, Rebekka Lencer, Eva Meisenzahl, Frauke Schultze-Lutter, Marlene Rosen, Theresa Lichtenstein, Lana Kambeitz-Ilankovic, Stephan Ruhrmann, Raimo K. R. Salokangas, Christos Pantelis, Stephen J. Wood, Boris B. Quednow, Giulio Pergola, Alessandro Bertolino, Nikolaos Koutsouleris, Joseph Kambeitz, the PRONIA Consortium, Nikolaos Koutsouleris, Dominic Dwyer, Anne Ruef, Lana Kambeitz-Ilankovic, Mark Sen Dong, Anne Erkens, Eva Gussmann, Shalaila Haas, Alkomiet Hasan, Claudius Hoff, Ifrah Khanyaree, Aylin Melo, Susanna Muckenhuber-Sternbauer, Janis Kohler, Omer Faruk Ozturk, David Popovic, Adrian Rangnick, Sebastian von Saldern, Rachele Sanfelici, Moritz Spangemacher, Ana Tupac, Maria Fernanda Urquijo, Johanna Weiske, Antonia Wosgien, Joseph Kambeitz, Stephan Ruhrmann, Marlene Rosen, Linda Betz, Theresa Lichtenstein, Karsten Blume, Mauro Seves, Nathalie Kaiser, Nora Penzel, Tanja Pilgram, Thorsten Lichtenstein, Julian Wenzel, Christiane Woopen, Stefan Borgwardt, Christina Andreou, Laura Egloff, Fabienne Harrisberger, Claudia Lenz, Letizia Leanza, Amatya Mackintosh, Renata Smieskova, Erich Studerus, Anna Walter, Sonja Widmayer, Rachel Upthegrove, Stephen J. Wood, Katharine Chisholm, Chris Day, Sian Lowri Griffiths, Paris A. Lalousis, Mariam Iqbal, Mirabel Pelton, Pavan Mallikarjun, Alexandra Stainton, Ashleigh Lin, Raimo K. R. Salokangas, Alexander Denissoff, Anu Ellila, Tiina From, Markus Heinimaa, Tuula Ilonen, Paivi Jalo, Heikki Laurikainen, Maarit Lehtinen, Antti Luutonen, Akseli Makela, Janina Paju, Henri Pesonen, Reetta-Liina Armio Säilä, Elina Sormunen, Anna Toivonen, Otto Turtonen, Ana Beatriz Solana, Manuela Abraham, Nicolas Hehn, Timo Schirmer, Paolo Brambilla, Carlo Altamura, Marika Belleri, Francesca Bottinelli, Adele Ferro, Marta Re, Emiliano Monzani, Mauro Percudani, Maurizio Sberna, Armando D’Agostino, Lorenzo Del Fabro, Giampaolo Perna, Maria Nobile, Alessandra Alciati, Matteo Balestrieri, Carolina Bonivento, Giuseppe Cabras, Franco Fabbro, Marco Garzitto, Sara PiCCuin, Alessandro Bertolino, Giuseppe Blasi, Linda A. Antonucci, Giulio Pergola, Grazia Caforio, Leonardo Faio, Tiziana Quarto, Barbara Gelao, Raffaella Romano, Ileana Andriola, Andrea Falsetti, Marina Barone, Roberta Passatiore, Marina Sangiuliano, Rebekka Lencer, Marian Surman, Olga Bienek, Georg Romer, Udo Dannlowski, Eva Meisenzahl, Frauke Schultze-Lutter, Christian Schmidt-Kraepelin, Susanne Neufang, Alexandra Korda, Henrik Rohner

**Affiliations:** 1 University of Cologne, Faculty of Medicine and University Hospital Cologne, Department of Psychiatry and Psychotherapy, Cologne, Germany; 2 Department of Psychiatry and Psychotherapy, Ludwig-Maximilian-University, Munich, Germany; 3 Group of Psychiatric Neuroscience, Department of Basic Medical Sciences, Neuroscience and Sense Organs, University of Bari ‘Aldo Moro’, Bari, Italy; 4 Max-Planck Institute of Psychiatry, Munich, Germany; 5 Department of Education, Psychology, Communication, University of Bari, Bari, Italy; 6 Department of Psychiatry, Brigham and Women’s Hospital, Harvard Medical School, Boston, MA USA; 7 Department of Nuclear Medicine, Bern University Hospital, Bern, Switzerland; 8 School of Psychology and Counselling, Queensland University of Technology, Brisbane, QLD Australia; 9 International Research Lab in Neuropsychiatry, Neuroscience Research Institute, Samara State Medical University, Samara, Russia; 10 Department of Psychosis Studies, Institute of Psychiatry, Psychology & Neuroscience, King’s College London, De Crespigny Park, London, SE5 8AF UK; 11 MRC London Institute of Medical Sciences, Hammersmith Hospital, London, W12 0NN UK; 12 Institute of Clinical Sciences, Faculty of Medicine, Imperial College London, London, W12 0NN UK; 13 South London and Maudsley NHS Foundation Trust, London, SE5 8AF UK; 14 Institute for Mental Health, University of Birmingham, Birmingham, UK; 15 Early Intervention Service, Birmingham Womens and Childrens NHS Foundation Trust, Birmingham, UK; 16 Department of Psychiatry (UPK), University of Basel, Basel, Switzerland; 17 Department of Psychiatry and Psychotherapy, University of Lübeck, Lübeck, Germany; 18 Department of Neurosciences and Mental Health, Fondazione IRCCUS Ca’ Granda Ospedale Maggiore Policlinico, University of Milan, Milan, Italy; 19 Department of Pathophysiology and Transplantation, University of Milan, Milan, Italy; 20 Department of Psychiatry and Psychotherapy, University of Münster, Münster, Germany; 21 Otto Creutzfeldt Center for Behavioral and Cognitive Neuroscience, University of Münster, Münster, Germany; 22 Department of Psychiatry and Psychotherapy, Medical Faculty, Heinrich-Heine University, Düsseldorf, Germany; 23 Department of Psychology, Faculty of Psychology, Airlangga University, Surabaya, Indonesia; 24 University Hospital of Child and Adolescent Psychiatry and Psychotherapy, University of Bern, Bern, Switzerland; 25 Department of Psychiatry, University of Turku, Turku, Finland; 26 Melbourne Neuropsychiatry Centre, University of Melbourne & Melbourne Health, Melbourne, VIC Australia; 27 Orygen, Melbourne, VIC Australia; 28 Centre for Youth Mental Health, University of Melbourne, Melbourne, VIC Australia; 29 Experimental and Clinical Pharmacopsychology, Department of Psychiatry, Psychotherapy, and Psychosomatics, Psychiatric Hospital of the University of Zurich, Lenggstr. 31, 8032 Zurich, Switzerland; 30 Institute of Psychiatry, Psychology & Neuroscience, Department of Psychosis Studies, King’s College London, London, UK; 31 General Electric Global Research Inc, Munich, Germany; 32 Workgroup of Paolo Brambilla, MD, PhD, University of Milan, Milan, Italy; 33 Programma 2000, Niguarda Hospital, Milan, Italy; 34 San Paolo Hospital, Milan, Italy; 35 Villa San Benedetto Menni, Albese con Cassano, Italy; 36 Workgroup of Paolo Brambilla, MD, PhD, Department of Medical Area, University of Udine, Udine, Italy; 37 IRCCUS Scientific Institute “E. Medea”, Polo FVG, Udine, Italy

**Keywords:** Psychosis, Neuroscience, Biomarkers

## Abstract

Continued cannabis use (CCu) is an important predictor for poor long-term outcomes in psychosis and clinically high-risk patients, but no generalizable model has hitherto been tested for its ability to predict CCu in these vulnerable patient groups. In the current study, we investigated how structured clinical and cognitive assessments and structural magnetic resonance imaging (sMRI) contributed to the prediction of CCu in a group of 109 patients with recent-onset psychosis (ROP). We tested the generalizability of our predictors in 73 patients at clinical high-risk for psychosis (CHR). Here, CCu was defined as any cannabis consumption between baseline and 9-month follow-up, as assessed in structured interviews. All patients reported lifetime cannabis use at baseline. Data from clinical assessment alone correctly classified 73% (*p* < 0.001) of ROP and 59 % of CHR patients. The classifications of CCu based on sMRI and cognition were non-significant (ps > 0.093), and their addition to the interview-based predictor via stacking did not improve prediction significantly, either in the ROP or CHR groups (ps > 0.065). Lower functioning, specific substance use patterns, urbanicity and a lack of other coping strategies contributed reliably to the prediction of CCu and might thus represent important factors for guiding preventative efforts. Our results suggest that it may be possible to identify by clinical measures those psychosis-spectrum patients at high risk for CCu, potentially allowing to improve clinical care through targeted interventions. However, our model needs further testing in larger samples including more diverse clinical populations before being transferred into clinical practice.

## Introduction

Cannabis use has a prominent role in the development of psychosis^1,2^, and exacerbates the course of the full-blown psychotic disorder^3,4^. Indeed, patients with psychosis who are habitual cannabis users have distinctly worse long-term outcome compared to those without concurrent cannabis use in terms of re-hospitalization, the severity of psychotic symptoms and general functioning^5^. In first episodes of psychosis, cannabis use is reportedly among the most powerful predictors of relapse to psychosis^6^. Likewise, in patients at clinical high-risk for psychosis (CHR) reporting lifetime cannabis use, continued cannabis use (CCu) after experiencing attenuated psychotic symptoms increased the risk of transition^7^. However, the number of individuals with cannabis use disorder has increased worldwide in recent years^8^, with correspondingly high rates of cannabis use disorder reported in early psychosis patients^9^. The vulnerability to CCu remains even after treatment to encourage cannabis abstinence^10^, and the response to abstinence interventions varies greatly between individuals^11^.

This body of evidence suggests that detecting individuals at risk for CCu as well as investigating and understanding the factors and mechanisms associated with CCu is from a preventive perspective important to improve the prospects for a good long-term outcome in CHR and recent-onset psychosis (ROP) patients^12^. In cannabis users drawn from community-based samples, sociodemographic factors such as young age, male sex, low income, higher body mass index (BMI) and substance use patterns each predicted relapse of cannabis use^13,14^. Further, the transition from irregular cannabis use to cannabis use disorder—a form of CCu that persists despite distress or impairment caused by the substance^15^—can be predicted by the pattern of substance use, as well as by mental health problems, history of traumatic events, schizotypal personality and living in an urban area^16–18^. Moreover, among clinically dependent cannabis users, poor current functioning predicts relapse of cannabis use^19,20^. Only one study^20^ has hitherto investigated predictors of cannabis relapse in psychotic patients (*N* = 66), wherein psychotic symptoms proved to be most predictive of relapse of cannabis use. However, a review of self-reported reasons for cannabis consumption by patients with psychosis^21^ concluded that present psychotic symptoms and self-medication are rarely reported as reasons for cannabis consumption. Instead, groups of psychotic patients^21^ and CHR patients^22^ both reported mood enhancement and social motives as their primary motivations for use. Cognitive deficits have been linked with relapse for several substances^23^, although the link with cognition is less consistently reported for cannabis compared to other substances^24^. Meta-analytic evidence of cognitive deficits attributable to cannabis use is complex, showing negative effects of cannabis on cognition in non-psychotic individuals, but also better preserved cognitive functions in psychotic patients with concurrent cannabis use^25^. Notably, the environmental risk factors for CCu, and the presence of cognitive deficits have also been individually associated with cannabis use in general, and with an increased risk for psychosis^26,27^.

Whether addiction—that is to say, a substance use disorder—should properly be called a “brain disease” remains a matter of debate^28^. Nonetheless, drug-seeking and relapse in the use of diverse substances, such as alcohol^29,30^ and cocaine^31^, have consistently been associated with underlying neurobiological alterations^32,33^. Interestingly, there is a substantial overlap between brain regions that are associated with drug-seeking in general, cannabis use disorder and psychosis^32–35^. Decreased grey matter volume (GMV) in the frontal cortex, hippocampus, insula and temporal lobe and increased volume in the cerebellar cortex, are common to all three conditions^32,34–37^. Further, effects of cannabis use on brain structure were more pronounced in psychotic individuals and individuals at clinical high-risk for developing psychosis compared to the effects in healthy individuals, potentially indicating a particular sensitivity to cannabis exposure^38^.

Several studies have investigated the association between these risk factors and cannabis relapse^13,20^ or the development of a cannabis use disorder^16–18^. Nevertheless, their power for predicting CCu in psychotic patients and their generalizability to other clinical cases—a precondition for model implementation into clinical practice^39^—have not yet been tested. Moreover, most studies have analyzed risk factors in isolation, without considering their potentially interconnected nature^40^. Progress in the field of predictive medicine using multivariable approaches has demonstrated that models enabling the simultaneous investigation of several risk factors and multiple data modalities can often outperform unimodal predictors for conversion to psychosis^41,42^, diagnostic approaches^43^ and functional outcome^37^.

In the current study, we (1) investigated multiple data modalities using machine learning^39^ to assess their power to predict CCu in patients with ROP. More specifically, we generated three predictive models of CCu based on single data modalities (*unimodal*); namely (i) clinical, (ii) cognitive and (iii) structural magnetic resonance imaging (sMRI)-based predictors. Next, we combined these models for super-ordinate prediction, to test whether combinations of unimodal predictors would improve the predictive performance of the algorithm. Then, (2) we applied the predictors to CHR individuals, aiming to assess the predictors’ generalizability to patients less severely affected in terms of psychotic symptoms and cannabis use. Finally, (3) we assessed how CCu is associated with several aspects of long-term clinical outcome to confirm previously published clinical relevance of CCu in ROP and CHR^4,5,7^. We hypothesized that there should emerge a pattern of interview-based variables at baseline that would predict CCu in our ROP sample above chance level and that, due to overlapping reasons for cannabis use between ROP and CHR patients^22^, this model would generalize well to a separate CHR population. Further, we hypothesized that including cognition and sMRI results would improve the algorithm’s predictive performance. In line with previous publications^4,5^ we expected that CCu would be associated with a worse long-term clinical outcome in ROP and CHR patients, thus highlighting the clinical relevance of the prediction.

## Results

### Sample characteristics

Overall, we included 182 patients (mean [SD] age, 23.8 [4.7] years and female = 68 [36.8%]) (Table 1) who all reported lifetime cannabis use at baseline. Eighty-seven patients (47%) had a CCu within a nine-month follow-up period, i.e. at least one cannabis consumption between baseline and follow-up. All other patients remained abstinent until at least nine months after baseline assessment and were labelled discontinued cannabis use *(DCu)*. Follow-up data for DCu patients was available on average for a mean (SD) of 597 (254) days from the baseline assessment. In this time period, only *N* = 8 (8.7%) subjects labelled as DCu had a relapse in cannabis use after the nine-month follow-up. On average, patients with CCu resumed cannabis consumption after a mean (SD) of 94 (100) days from the baseline assessment. The time between baseline and renewed cannabis use did not significantly differ between CHR and ROP groups (mean [SD], 87 [100] days for CHR and 97 [102] days for ROP; t_32_ = −0.33, *p* = 0.744). We trained and tested our model in repeated nested cross-validation strictly separating training and testing folds on *N* = 109 patients of age 15–40 years with ROP and tested our model in a separate group of *N* = 73 CHR patients.

**Table 1:** Demographic information of patients with recent-onset psychosis and patients with clinical high-risk for psychosis.

	CCu	DCu	Statistical analysis	*p*	CCu	DCu	Statistical analysis	*p*
Discovery sample (ROP; *N* = 109)					Validation sample (CHR; *N* = 73)			
Sample Size [*N* (%)]	54 (49.5)	55 (50.5)			36 (49.3)	37 (50.7)		
Sample Size per Study Site [*N* (%)]:								
Munich (%)	29 (53.7)	25 (45.5)	χ^2^_9_ = 11.90	0.156	12 (33.3)	15 (40.5)	χ^2^_9_ = −8.81	0.455
Milan (%)	4 (7.4)	3 (5.5)			2 (5.6)	2 (5.4)		
Basel (%)	9 (16.7)	3 (5.5)			4 (11.1)	1 (2.7)		
Cologne (%)	2 (3.7)	9 (16.4)			6 (16.7)	10 (27.0)		
Birmingham (%)	3 (5.6)	3 (5.5)			0 (0.0)	2 (5.4)		
Turku (%)	4 (7.4)	7 (12.7)			3 (8.3)	2 (5.4)		
Udine (%)	0 (0.0)	0 (0.0)			1 (2.8)	0 (0.0)		
Bari (%)	0 (0.0)	2 (3.6)			0 (0.0)	1 (2.8)		
Duesseldorf (%)	0 (0.0)	1 (1.8)			3 (8.3)	2 (5.4)		
Muenster (%)	3 (5.6)	2 (3.6)			5 (13.9)	2 (5.4)		
Time of Relapse [mean (SD) days after Baseline]	97.4 (102.0)	–			87 (100.1)	–		
Age [mean (SD) years]	23.8 (4.3)	25.1 (5.2)	t_104_ = −1.45	0.151	22.0 (3.7)	23.8 (5.2)	t_65_ = −1.80	0.076
Sex [Female (%)]	15 (27.8)	19 (34.5)	χ^2^_1_ = 0.31	0.578	**11 (30.6)**	**23 (62.2)**	**χ**^**2**^_**1**_ = **−6.11**	**0.013**
Race/ethnicity [*N* (%)]								
White (%)	43 (79.6)	42 (76.4)	χ^2^_5_ = −3.06	0.691	28 (77.8)	35 (94.6)	χ^2^_3_ = −5.64	0.131
Asian (%)	4 (7.4)	5 (9.1)			2 (5.6)	0 (0)		
African (%)	1 (1.9)	1 (1.8)			2 (5.6)	1 (2.7)		
Mixed (%)	4 (7.4)	3 (5.5)			0 (0)	0 (0)		
Other (%)	1 (1.9)	4 (7.3)			4 (11.1)	1 (2.7)		
BMI [mean (SD)]	23.1 (4.1)	22.9 (4.0)	t_97_ = −0.30	0.763	23.5 (4.4)	22.0 (2.9)	t_59_ = 1.71	0.092
Education [mean (SD) years]	13.3 (2.7)	13.9 (2.7)	t_105_ = −1.12	0.268	13.1 (2.6)	14.2 (2.7)	t_70_ = −1.64	0.106
Educational problems [mean (SD) years repeated]	0.7 (1.8)	0.4 (0.7)	t_67_ = 1.08	0.282	0.8 (2.2)	0.9 (2.5)	t_68_ = −0.21	0.837
GF-Social: highest lifetime	7.8 (0.8)	8.0 (0.8)	t_106_ = −1.36	0.177	7.7 (0.9)	8.1 (0.8)	t_68_ = −1.64	0.107
GF-Social: baseline	5.6 (1.5)	5.9 (1.5)	t_106_ = −0.94	0.352	5.9 (1.4)	6.6 (1.5)	t_71_ = −1.93	0.058
GF-Role: highest lifetime	**7.4 (0.9)**	**7.9 (0.9)**	**t**_**105**_ = **−2.67**	**0.009**	7.9 (0.9)	7.9 (0.8)	t_69_ = −0.16	0.877
GF-Role: baseline	4.6 (1.7)	5.3 (1.9)	t_106_ = −1.96	0.052	5.4 (1.9)	6.0 (1.4)	t_64_ = −1.60	0.114
GAF Disability/Impairment Highest Lifetime	77.5 (8.8)	78.5 (9.0)	t_104_ = −0.58	0.561	76.6 (8.6)	79.0 (8.3)	t_71_ = −1.22	0.227
GAF Disability/Impairment Highest Past Month	**41.3 (10.4)**	**49.0 (16.6)**	**t**_**91**_ = **−2.92**	**0.004**	48.3 (11.3)	53.0 (11.2)	t_70_ = −0.52	0.607
GAF Symptoms Highest Lifetime	77.7 (8.5)	79.6 (9.6)	t_106_ = −1.07	0.286	78.0 (9.9)	79.1 (8.8)	t_71_ = −1.78	0.079
GAF Symptoms Highest Past Month	40 (12.4)	43.4 (16.4)	t_100_ = −1.21	0.230	47.0 (10.2)	51.8 (11.8)	t_70_ = −1.86	0.067
Positive and Negative Syndrome Scale—Positive [mean (SD)]	20.0 (5.9)	19.2 (6.0)	t_105_ = 0.69	0.491	11.2 (3.5)	11.5 (3.1)	t_67_ = −0.36	0.718
Positive and Negative Syndrome Scale—Negative [mean (SD)]	15.2 (5.9)	14.2 (6.5)	t_104_ = 0.87	0.388	13.8 (6.8)	13.9 (6.0)	t_67_ = −0.07	0.941
Positive and Negative Syndrome Scale—General [mean (SD)]	**37.0 (10.8)**	**31.8 (9.5)**	**t**_**103**_ = **2.66**	**0.009**	30.8 (7.7)	29.5 (7.6)	t_68_ = 0.72	0.474
Becks Depression Inventory [mean (SD)]	22.9 (14.2)	19.1 (11.5)	t_93_ = 1.44	0.152	27.9 (11.5)	27.5 (10.4)	t_65_ = 0.14	0.891
Lifetime history of DSM-IV Cannabis use disorder [*N* (%)]								
Cannabis abuse (%)	**21 (38.9)**	**19 (34.5)**	**χ**^**2**^_**2**_ = −**9.61**	**0.008**	16 (44.4)	12 (32.4)	χ^2^_2_ = −1.14	0.566
Cannabis dependence (%)	**17 (31.5)**	**6 (10.9)**			1 (2.8)	1 (2.7)		
Time since last Cannabis Use at Baseline [mean (SD) months]	**8.7 (25.3)**	**33.4 (60.9)**	**t**_**66**_ = **−2.52**	**0.010**	**10.6 (29.2)**	**33.6 (53.8)**	**t**_**50**_ = **−2.14**	**0.038**
Duration Lifetime Cannabis Use [mean (SD) months]	63.7 (50.0)	51.8 (43.9)	t_90_ = 1.22	0.227	52.9 (64.4)	33.7 (43.4)	t_45_ = 1.31	0.195
Age at Cannabis Initiation [mean (SD) years]	16.5 (2.6)	17.5 (3.7)	t_86_ = −1.55	0.126	17.1 (2.2)	17.5 (2.7)	t_56_ = −0.64	0.526
Average Number of cigarettes smoked per day [mean (SD)]	8.0 (7.4)	7.2 (7.6)	t_100_ = 0.60	0.553	7.7 (8.6)	4.6 (6.1)	t_63_ = 1.71	0.093
Average Units of alcohol consumed per day [mean (SD)]	4.4 (4.2)	5.4 (6.1)	t_75_ = −0.92	0.360	4.2 (5.6)	4.0 (3.3)	t_46_ = 0.24	0.811

Bold: significant at *p* < 0.05.

*DCu* discontinued cannabis use, *CCu* continued cannabis use, *BMI* body mass index, *ROP* recent-onset psychosis, *CHR* clinical high-risk for psychosis, *GAF* Global Assessment of functioning, *GF* global functioning, *DSM-IV* Diagnostic and Statistical Manual of Mental Disorders, 4th edition, *SD* standard deviation.

In the ROP and CHR groups, CCu was significantly associated with more recent cannabis consumption at baseline. CHR patients with CCu were more likely to be male than those with DCu (χ^2^_1_ = −6.11, *p* = 0.013). ROP patients with CCu had significantly lower lifetime highest role functioning (t_105_ = −2.67, *p* = 0.009), more severe Positive and Negative Syndrome Scale (PANSS)^44^—general scores (t_103_ = 2.66, *p* = 0.009), as well as a higher number of SCID-IV diagnoses for cannabis use disorder compared with ROP patients with DCu (χ^2^_2_ = −9.61, *p* = 0.010; Table 1). Due to missing information or inadequate MR image quality, our samples differed slightly for the predictors based on cognition (N_ROP_ = 105, N_CHR_ = 73) and sMRI (N_ROP_ = 101, N_CHR_ = 61) (Supplementary Fig. 7).

### Prediction of continued cannabis use

Only the unimodal predictor based exclusively on clinical predictors yielded significant prediction of CCu in ROP patients (balanced accuracy (BAC) = 73.3%, *p* = 0.001). Further, this model had an acceptable Area Under the Curve (AUC = 0.75) as defined previously (AUC ≥ 0.7^45^). Applied to the CHR group, the BAC dropped significantly by 14.2% points (*p* < 0.001, Supplementary Fig. 8) but still provided a correct prediction in 58.7% of the CHR patients. The sMRI predictor performed with a BAC of 55.7% (*p* = 0.093) in the ROP and of 54.6% in the CHR group. The cognitive predictor performed below chance level in both groups (ROP: BAC = 45.6%; CHR: BAC = 49.7%). The clinical prediction accuracies could not be better explained by confounding effects (Supplementary 4), but sensitivity and specificity differed significantly depending on whether the criterion of cannabis use disorder in a lifetime was fulfilled (Supplementary 6). Stacking our significant clinical predictor with the sMRI-, the cognitive- or both predictors did not improve performance in the ROP group (BAC = 66.0–67.8%). The stacked predictors including sMRI yielded similar results as the clinical predictor when applied to the CHR group (BAC = 58.7%) (Table 2). Likewise, combining the clinical with the cognitive predictor did not significantly improve the prediction when applied to CHR compared with the unimodal clinical predictor (BAC = 60.0%, *p* = 0.065, Supplementary Fig. 8).

**Table 2:** Prediction results of unimodal and multimodal predictors.

	TP	TN	FP	FN	Sens%	Spec%	BAC%	PPV	NPV	PSI	NLR	PLR	AUC	*p*-value
Clinical predictor														
ROP (*N* = 109)	38	42	13	16	70.4	76.4	73.4	74.5	72.4	46.9	0.4	3.0	0.75	<0.001
Applied to CHR (*N* = 73)	15	28	9	21	41.7	75.7	58.7	62.5	57.1	19.6	0.8	1.7	0.65	NA
Cognitive predictor														
ROP (*N* = 106)	36	14	38	17	67.9	26.9	47.4	48.6	45.2	-6.2	1.2	0.9	0.41	0.763
Applied to CHR (*N* = 73)	26	12	25	10	72.2	32.4	52.3	51.0	54.5	5.5	0.9	1.1	0.48	NA
sMRI predictor														
ROP (*N* = 101)	39	17	34	11	78.0	33.3	55.7	53.4	60.7	14.1	0.7	1.2	0.56	0.093
Applied to CHR (*N* = 61)	25	8	25	5	83.3	25.8	54.6	52.1	61.5	13.6	0.6	1.1	0.68	NA
Stacked predictor (clinical and sMRI)														
ROP (*N* = 109)	34	40	15	20	63.0	72.7	67.8	69.4	66.7	36.1	0.5	2.3	0.73	0.001
Applied to CHR (*N* = 73)	15	28	9	21	41.7	75.7	58.7	62.5	57.1	19.6	0.8	1.7	0.67	NA
Stacked predictor (clinical and cognition)														
ROP (*N* = 109)	34	39	16	20	63.0	70.9	66.9	68.0	66.1	34.1	0.5	2.2	0.71	0.005
Applied to CHR (*N* = 73)	15	29	8	21	41.7	78.4	60.0	65.2	58.0	23.2	0.7	1.9	0.65	NA
Stacked predictor (clinical and sMRI and cognition)														
ROP (*N* = 109)	35	37	18	19	64.8	67.3	66.0	66.0	66.1	32.1	0.5	2.0	0.71	0.004
Applied to CHR (*N* = 73)	15	28	9	21	41.7	75.7	58.7	62.5	57.1	19.6	0.8	1.7	0.68	NA

*ROP* recent-onset psychosis, *CHR* clinical high-risk for psychosis, *TP* true positive, *TN* true negative, *FP* false positive, *FN* false negative, *Sens* sensitivity, *Spec* specificity, *BAC* balanced accuracy, *PPV* positive predictive value, *NPV* negative predictive value, *PSI p*rognostic summary index, *PLR* positive likelihood ratio, *NLR* negative likelihood ratio, *AUC* area under the curve.

### Predictive patterns of the clinical classifier

**Fig. 1:**
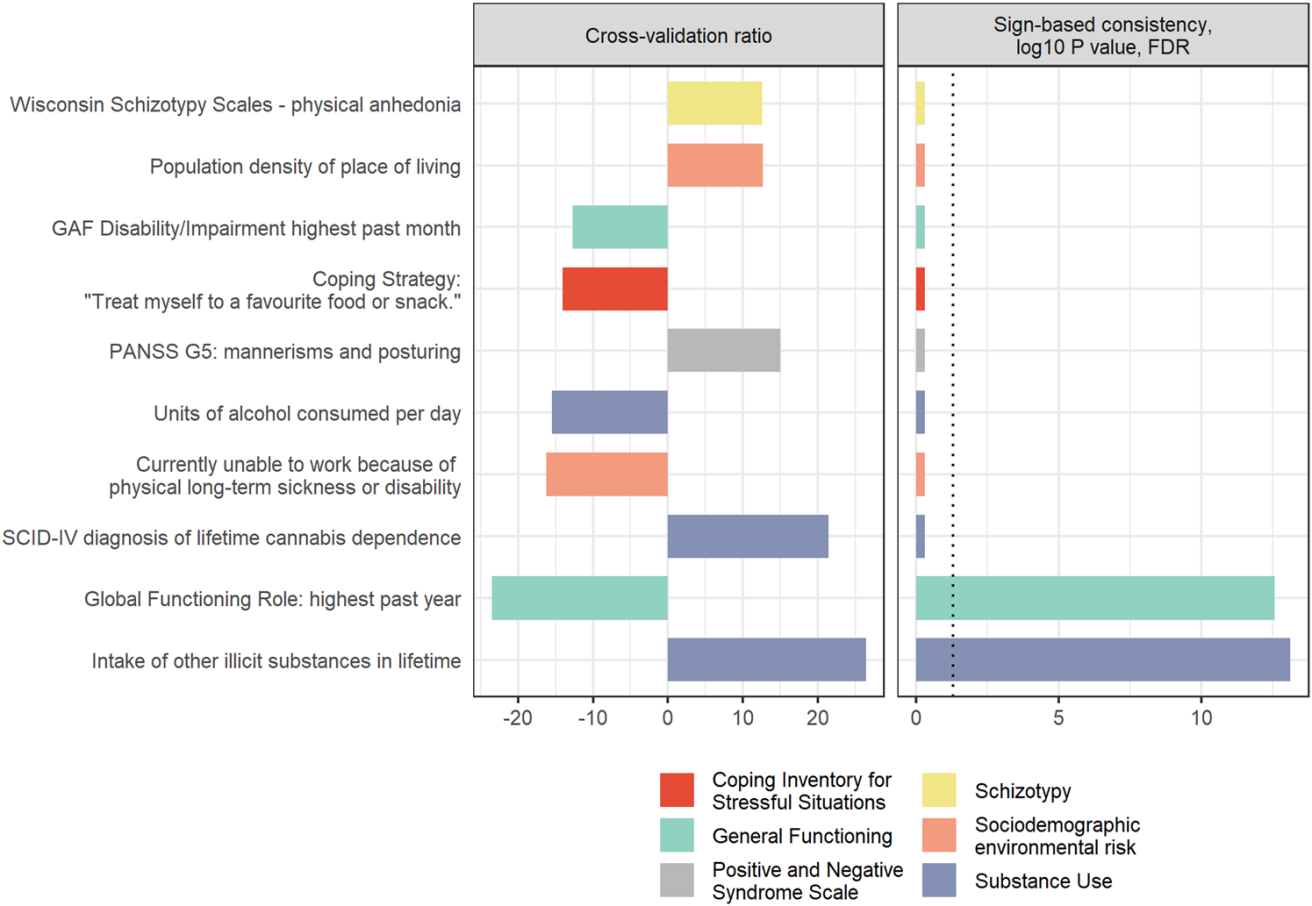
Feature importance. Top ten most predictive clinical variables differentiating between continued and discontinued cannabis use until nine-month follow-up in terms of cross-validation ratio (left-side) and significant predictive features measured in terms of sign-based consistency (right-side). GAF Global Assessment of Functioning, FDR false discovery rate, PANSS G Positive and Negative Syndrome Scale—General symptoms, SCID Structured Clinical Interview for DSM Disorders.

Features from different categories contributed reliably to the clinical classifier (Fig. 1). The significant and most reliable features predicting CCu were a higher number of substances from other substance classes tried in a lifetime and a lower lifetime highest role functioning. Further reliable predictors of CCu were a higher number of lifetime diagnoses of cannabis dependence and a lower number of units of alcohol consumption at drinking occasions, as well as lower functional disability scores of the split version of the Global Assessment of Functioning (GAF-F)^46,47^ score in the past month. A higher population density of place of living, higher physical anhedonia, less frequent use of favourite food as a coping strategy and more severe mannerisms and posturing were also reliable predictors of CCu. Further, an increased likelihood of being currently unable to work because of long-term physical illness was one of the top ten most predictive features of DCu. However, this variable might be spurious, as only one ROP patient with DCu replied to this query with “yes”, while all other CCu and DCu patients replied with “no” or did not respond to this question (16.5% missing answer, Supplementary Table 2 for %-missing of features and Supplementary Table 8 for univariate comparisons between CCu and DCu for all clinical variables included in the prediction).

### Exploration: Continued cannabis use and long-term clinical outcome

**Fig. 2:**
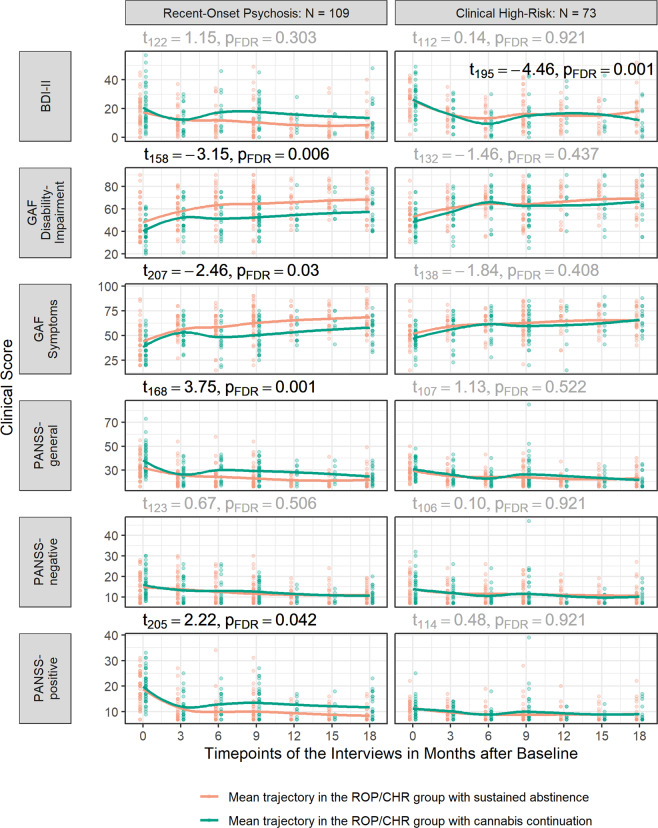
Association of continued cannabis use and long-term clinical outcomes. Association of continued cannabis use with the long-term course of several clinical outcomes from baseline till 18 months follow-up. Linear-mixed models were calculated modelling the clinical outcome as dependent variable and group (continued cannabis use/discontinued cannabis use), time since baseline, linear trends, quadratic trends and trend interactions as independent variable. Subject entered as random effect. Significant group effects are marked in black above and significant interactions effects are marked in black within the graphs. False-discovery rate correction was performed to control for the number of comparisons for each fixed effect across the clinical outcome variables. Of note: For graphical depiction, time from baseline is presented as ordinal variable, however, in the model calculation the time from baseline entered as a continuous variable. Further, as the model fit for the optimal complexity varied by outcome the regression-line in the plot is modelled with the ‘LOESS’ nonparametric function. PANSS Positive and Negative Syndrome Scale, GAF Global Assessment of Functioning, BDI-II Beck’s Depression Inventory-II, ROP recent-onset psychosis, CHR clinical high-risk for psychosis.

Following investigation of long-term effects of CCu by employing linear-mixed effects models (Fig. 2, Supplementary Table 10 for further details on all models calculated), our results showed that, on average, clinical measures improved in ROP patients over the 18 months follow-up period (all p_FDR_ < 0.007). In the ROP group, CCu was significantly associated with lower GAF-F (t_136_ = −3.15, p_FDR_ = 0.006), lower current symptoms of the GAF Symptoms (GAF-S) (t_167_ = −2.46, p_FDR_ = 0.030), higher PANSS-general scores (t_168_ = 3.75, p_FDR_ = 0.001) and higher PANSS-positive score (t_205_ = 2.22, p_FDR_ = 0.042), while CCu did not significantly predict the sum score of the Becks Depression and Inventory-II (BDI-II)^48^ (t_122_ = 1.15, p_FDR_ > 0.303). There were no significant interaction effects between time and CCu in ROP patients (all p_FDR_ > 0.060). In the CHR patients, all clinical measures besides BDI-II improved over the 18 months follow-up period (all p_FDR_ < 0.001). There was a significant time-by-group interaction for BDI-II (linear: t_195_ = −4.46, p_FDR_ < 0.001, quadratic: t_199_ = 3.89, p_FDR_ < 0.001, cubic: t_199_ = −3.35 p_FDR_ < 0.003), but no significant main effect of CCu on any of the clinical outcomes (all p_FDR_ > 0.220).

## Discussion

This is the first multivariable study examining the predictability of CCu in individuals with ROP and CHR based on unimodal and multimodal data domains. Our study adds to previous investigations by indicating (1) a potentially generalizable predictor for risk of CCu in a sample of patients who are particularly vulnerable to the harmful effects of cannabis consumption^4^, and (2) by revealing a pattern of factors that might be further investigated to ultimately inform the design of tailored preventive strategies.

We found evidence supporting the feasibility of generalizable and significant prediction, correctly predicting CCu and DCu within nine months after baseline in 73.3% of ROP patients, based solely on their baseline clinical data. This model generalized to CHR patients only slightly above chance (BAC = 58.7%). The most important predictors of CCu were lower lifetime best role functioning and the lifetime number of illicit substances consumed other than cannabis. Predictive performance was not improved by augmenting the model with cognitive or GMV data. Further, we found that CCu was significantly associated with worse clinical outcomes in psychotic patients, and interacted with longitudinal depressive symptoms in at-risk individuals, thus confirming the importance of timely efforts to discourage CCu in these clinical groups.

### Baseline clinical predictors of continued cannabis use

Our finding that the predictive power of interview-based variables outperforms other data modalities is in line with earlier results in CHR and ROP samples presenting predictive models of other clinical outcomes, such as treatment outcome after a first episode^40^, transition to psychosis^41,49–51^ or global functioning^37^.

We confirmed the importance of global functioning as an important predictor of CCu^52,53^ and extended previous literature in two ways. First, we assessed the model’s subject-specific predictive power and generalizability to at-risk individuals and investigated its effect by considering diverse factors simultaneously. Furthermore, our results reemphasize the importance of investigating broad aspects of global functioning in patients with psychosis^37,49,54^. Interestingly, CCu was mainly associated with lower levels of highest functioning. Assuming that the suboptimal functioning was also in part subjectively experienced, the lack of subjective well-functioning in several domains over a longer time period might lead to lower self-expectations, which are known to undermine abstinence^55^. The predictive power of lifetime diagnosis of cannabis dependence was expected because the diagnostic criteria of cannabis use disorder inherently entail an elevated likelihood to CCu^8,56^. The importance of the number of lifetime illicit substances is also in line with the literature^13,17,18^. Conversely, lower average alcohol consumption at drinking occasions predicted CCu. This is an interesting novel finding, as the literature has so far been inconclusive whether alcohol is typically used as a substitution or complementary to cannabis use^57^. Our finding would rather support the substitution hypothesis for alcohol use, which is in line with a previous study^58^ reporting changes in alcohol consumption patterns during cannabis abstinence. In line with that evidence, we found that patients with CCu were less likely to use food or snacks as a coping strategy in stressful life situations. Additionally, the CCu patients presented with higher physical anhedonia, a decreased ability to experience pleasure, which might reflect a general lack of coping strategies against relapse to cannabis use. Importantly, this conjecture is supported by studies showing that mood enhancement and social factors are the primary motivations for cannabis consumption in patients with psychosis^21^ and CHR patients^22^. Further, we replicated earlier findings on the importance of higher population density of place of living^18^ as a predictive risk factor of CCu. The population density was previously shown not only to be predictive of cannabis relapse, but also of lifetime cannabis use and psychosis^26,59^, suggesting that urbanicity and cannabis use may interact to increase the risk for psychosis^60,61^. Future studies should disentangle the specific impact of these two factors on psychosis.

### Validation of the clinical predictor in clinical high-risk patients

Our clinical predictor performed only slightly above the chance level when applied to CHR patients. Indeed, univariate statistics (Supplementary Table 8) show that several of the most important clinical predictors did not significantly differ between CCu and DCu among CHR patients. Importantly, the CHR group had a lower proportion of subjects with cannabis use disorder compared with the ROP group, which might indicate that even the CCu individuals among CHR patients are less heavy cannabis users. As our predictor seems to be more sensitive to patients with cannabis use disorder (Supplementary 6), further investigations and testing in more diverse clinical populations are warranted. Applying our predictor to CHR patients with concurrent cannabis use disorder, to ROP patients with and without cannabis use disorder, as well as non-psychotic individuals with cannabis use disorder might disentangle the coupling between psychotic symptoms and cannabis use.

### sMRI predictor of continued cannabis use

Contrary to expectation, our sMRI predictor did not perform significantly better than chance. This might be related to the study-specific outcome: CCu was defined as any cannabis consumption between baseline and nine-month follow-up. Most previous studies have instead investigated associations between more severe forms of cannabis use and GMV^32^. One study^62^ attempted to predict future cannabis use in 14-year-old abstinent adolescents, defined as at least ten instances of cannabis use during two years follow-up, with the finding that GMV differences did not precede cannabis use. Although general use in predictive models of additional and costly sMRI would not be justified, it still merits testing in future studies including larger samples to see if sMRI might help to predict more severe forms of CCu. Notably, although we carefully corrected for site-specific MR variation (Supplementary), the unbalanced sample sizes across sites might nonetheless have impacted the predictive accuracy of sMRI.

### Cognitive predictor of continued cannabis use

Cognition did not predict CCu above chance level, which was surprising since schizophrenia is characterized by severe impairments in cognition^43,63^, as is likewise heavy cannabis use^64^. On the other hand, a previous meta-analysis has shown that the evidence is inconclusive for an association between cannabis dependence and cognitive impairments^24^. This inconsistency might be explained by differences in the cognitive tests analyzed, as some performance deficits have been shown to be task-specific^65^. Moreover, other evidence shows that cognition is better preserved in cannabis-using psychotic individuals than in patients without concurrent cannabis use^25,66^, and hence cognition might be a less important factor for predicting CCu in this particular patient group. Furthermore, a recent review on acute and residual effects of cannabis on cognition^67^ concluded that the association between cognition and cannabis is likely explained by genetic and environmental factors that predispose certain individuals both to cannabis use and cognitive deficits, and to a lesser degree by actual neurotoxic effects. Future studies are warranted to disentangle whether these negative results reflect our use of tests that are insensitive to particular cognitive changes predicting CCu, or whether cognitive disturbances are indeed not predictive of CCu in psychotic and at-risk patients.

### Effect of continued cannabis use on long-term clinical outcome

Our longitudinal analyses partially support the notion that CCu increases the risk for a poor long-term outcome in ROP and CHR individuals. Even though we found significant differences between CCu and DCu for almost all clinical outcome measures in the ROP group, we found a significant interaction between time and CCu only with depression in the CHR group. Depressive symptoms are a common comorbidity in patients with recent-onset psychosis^68^. However, our finding was unexpected since other studies investigating the impact of altered cannabis use on depressive symptoms have been so far inconclusive^52,69^. In the ROP group, we found a trending interaction effect of CCu with general symptoms over time, which would be in line with the previous literature^52^. There are several possible explanations for these non-significant interaction effects: First, patients with DCu have been longer abstinent than CCu patients, and thus they might already have recovered from the detrimental effects of the cannabis consumption. Second, our analyses might have been less sensitive to time-dependent effects due to the attrition rate in our study, leading to missing data and a relatively small sample size. Third, some of the patients with CCu have reported only one cannabis use at follow-up. Previous studies have shown that even a decreased CCu might improve the long-term clinical outcome^4^. Future studies might investigate further baseline measures to disentangle the main effects of CCu versus general cannabis use.

### Limitations

Among the important limitations of our study, we note that missing assessments in several subjects for some timepoints hindered analysis of time-to-event data, which might otherwise have improved accuracy by disentangling further subjects’ risk^42^. Additionally, the patient population of our study is difficult to contact and typically present a high attrition rate^4^. Thus, the follow-up period was only nine months, and our final sample was relatively small and unbalanced across sites, which might well have influenced results—especially in the imaging domain. Even though we carefully corrected for site effects, future studies are needed to investigate thoroughly and replicate our findings in larger samples and across sites. This would also be important to validate the speculation, as might arise from our findings, that MRI and cognitive measures are not of pivotal importance for predicting continued cannabis use. Even though most individuals who remained abstinent during the nine-month follow-up remained abstinent thereafter, further studies are warranted specifically to investigate the long-term prediction of continued cannabis use. Furthermore, our relatively small sample size hindered a further stratification of the critical outcome “continued cannabis use”. Future studies might also assess the predictability of different severities of CCu. Indeed, any reduction in cannabis use improves psychosis outcome^5^, and may be a more realistic harm reduction aim in therapy than complete abstinence^52^. As such, it would be useful to predict the relevant amount of cannabis use as distinct from complete abstinence. Most critically, our study lacks an external validation of the prediction of CCu in ROP. Thus, it cannot be inferred whether the drop in the accuracy of our predictor is better explained by low generalizability or by the differences of our samples in terms of severity of clinical symptoms and substance use. Hence, future tests of generalizability in ROP samples with similar substance use profiles are called for. Moreover, our study lacks some variables with known associations with cannabis use disorder, such as the individual’s motivation to quit cannabis use^53^ or specific substance-related cognitive tests^65^, the inclusion of which might improve accuracy in future studies. Importantly, cannabis use was assessed via self-report, which might suffer from recall- and social desirability bias. Ideally, future studies should confirm cannabis use and ascertain cannabis abstinence by biological measurements, preferably via hair toxicology, given its long detection window^70^.

## Conclusion

This is the first multimodal examination of prognostication of CCu in ROP patients, along with generalizability testing in CHR patients. We found that the best predictor was based solely on clinical variables, reliably showing a contribution of global functioning, especially lower highest lifetime functioning, specific patterns of substance use, urbanicity and a lack of coping strategies. This predictor might be improved in future studies by adding specific cannabis-related questionnaires or additional data modalities such as cortical thickness, genetics or functional MRI, aiming to improve its clinical utility. Importantly, the ultimate aim to identify better those patients with ROP or CHR who are most likely to continue cannabis use, enabling tailored interventions and thus improve their clinical outcome, calls for testing and improvement of the model in larger and more diverse clinical samples.

## Methods

### Study design and population

As part of the multisite ‘Personalized Prognostic Tools for Early Psychosis Management’ study (PRONIA [www.pronia.eu, German Clinical Trials Register identifier DRKS00005042^37^]) *N* = 80 patients of age 15–40 years with ROP and *N* = 73 CHR patients were included. A further *N* = 29 patients of age 18–40 years with ROP were recruited within the monocentric, longitudinal cannabis-induced psychosis study (CIP)^71^. The ROP group included via PRONIA had experienced an affective or non-affective psychotic episode within the past 24 months that was present within the past three months prior to study entry. The ROP group included in CIP had a psychosis diagnosis originally associated with cannabis use that preceded the onset of psychotic symptoms by no more than two weeks in the last 24 months, as defined in the International Classification of Diseases, 10th Revision, criteria for substance-induced psychosis^72^. CHR individuals needed to fulfil (1) the basic symptom criterion “Cognitive Disturbances” assessed by the Schizophrenia Proneness Instrument^73^; and/or (2) a slightly adapted version of the ultra-high-risk criteria according to the Structured Interview for Psychosis-Risk Syndromes^74^.

ROP patients included in CIP were recruited at the Department of Psychiatry of Ludwig-Maximilian-University in Munich, while both PRONIA samples were recruited at ten different European sites (see ref. ^41^). Diagnoses were based on internationally established criteria and given by trained clinical raters^37,71^. Current or past alcohol dependence and polysubstance dependence within the past six months were exclusion criteria (Supplementary 1 for general exclusion criteria). Further, ROP and CHR patients included via PRONIA had to be abstinent from cannabis in the four weeks prior to inclusion. We imposed an additional inclusion criterion, only admitting patients with lifetime cannabis use prior to baseline.

All patients from PRONIA underwent baseline assessment between 2014 and 2019 and were followed for up to 36 months. The CIP recruitment took place from December 2016 until May 2019, and the follow-up period was nine months. The study protocols were largely harmonized (detailed assessments are listed in Supplementary Table 1).

Prior to inclusion, all patients provided written, informed consent (either personally or through a legal guardian if below the age of 18). Studies were approved at their respective sites by the local research ethics committees.

### Outcome target

Substance use was assessed in a semi-structured interview at each visit^71^ (Supplementary Fig. 1). At the baseline interview, clinical raters asked the patient about his/her history of cannabis use and subsequently if he/she had used cannabis since the previous examination. We defined *CCu* as any cannabis consumption between baseline and nine month follow-up. Conversely, we labelled each patient who remained abstinent until at least nine months after baseline assessment as discontinued cannabis use *(DCu)*.

### Definition of the predictors

We trained three unimodal classifiers: (i) clinical, (ii) cognitive and (iii) sMRI (Supplementary Table 2 for the full list of variables). Predictors for the clinical domain were selected based on their prior association with cannabis use, consisting of: (1) substance use-related items^56,71^, (2) environmental risk-factors^16^, (3) clinical symptoms^8,19,20^, (4) global functioning^75^, (5) stress and coping strategies information^76^, (6) demographic data and (7) the BMI^14^. The cognitive predictor variables were selected from subscores of the cognitive domains of the MATRICS Consensus Cognitive Battery^77^, following the previous approaches^41^. The sMRI classifier was based on whole-brain GMV. A harmonized protocol for the acquisition of sMRI data was used at all sites^37^. For pre-processing, we used the open-source CAT12 toolbox (version r1155; http://dbm.neuro.uni-jena.de/cat12/), which is an extension of SPM12 running in MATLAB 2018a (Supplementary 2 and Supplementary Table 3 for details of sMRI acquisition and pre-processing). We employed group information guided–independent component analysis (GIG-ICA)^78^, which simultaneously takes into account the covariance between brain voxels and their similarity to reference components (RCs) of interest^71,79^. We chose nine RCs^34^ previously shown to be linked with schizophrenia^34^, which included several regions that have also been associated with cannabis use disorder, namely the prefrontal cortex, insula and cerebellum^32^ (Supplementary Fig. 2 for RCs).

### Machine learning strategy

We generated and tested our predictors on the total sample of ROP patients (*N* = 109). Next, we tested if our predictors would generalize to CHR patients (*N* = 73). Our machine learning pipeline was implemented in NeuroMiner version 1.1 (www.pronia.eu/neurominer) running in MATLAB R2019. To build the set of predictors, we strictly separated the training and test phases in repeated nested cross-validation (CV) with ten folds and five permutations both at the outer (CV_2_) and inner cycles (CV_1_). All features of the (i) clinical and (ii) cognitive predictors were standardized based on the median, with imputation of missing values by Seven-Nearest Neighbour imputation, and pruning of non-informative features (zero-variance, infinity). Subsequently, all features were scaled from zero to one. To find a set of optimally predicting features, we employed a wrapper-based feature selection using linear support vector machines (SVM; LIBSVM 3.12^80^; http://www.csie.ntu.edu.tw/~cjlin/libsvm). Following a previous approach^41^, we trained the models on the CV_1_ training data and picked the best-performing models based on the average SVMs (BAC) at the CV_1_ training and testing data. More specifically, we performed a greedy sequential forward search^81^ across the range of the SVM *C* regularization parameters ($2^{[-4_\in{\mathbb{Z}}\rightarrow +4]}$^41^), adding one feature at a time until the top ten percent most predictive features were selected.

For the (iii) sMRI-based predictor, we accounted for site-specific heterogeneity in two steps. First, we used the so-called g-theory mask^37,41^ to exclude all voxels showing only between-site but no inter-subject variation^71,82^. Second, we adjusted the remaining voxels for site effects using ComBat^83,84^, a harmonization method based on an empirical Bayesian approach, frequently used to remove non-biological variation related to differences between MRI scanners. To preserve the biological variation of interest (CCu), we used ComBat on a subsample of healthy individuals from PRONIA that was matched for age and sex between sites (Supplementary Fig. 3, and Supplementary Table 4 for age and sex distribution of matched healthy control sample, Supplementary Fig. 4, and Supplementary Table 5 for pre/post comparisons). This model was then applied independently to our discovery (ROP) and validation samples (CHR) (Supplementary Fig. 5, Supplementary Table 6). Finally, the thresholded and site-corrected sMRI images entered our machine learning pipeline. Strictly separating between CV_1_ and CV_2_, we first scaled total intracranial volume proportionally from each voxel. We then corrected for sex and age effects based on betas computed in our healthy control subsample and employed GIG-ICA to reduce feature dimensionality. Next, the components were scaled between zero and one. Again, we employed an SVM^80^ with optimization of the *C*-parameter within a range from $2^{[-4_\in{\mathbb{Z}}\rightarrow+4]}$^41^. See Supplementary 3 for a detailed description of sMRI processing and Supplementary Fig. 2 for an overview of all steps.

### Multimodal prediction models

To combine our best-performing unimodal (i) clinical predictor with the other unimodal predictors we used a stacked generalization procedure^37^. Here, the CV_1_-test decision scores from unimodal predictors served as features within the same CV structure and were scaled from zero to one, with the imputation of any missing sMRI and cognitive data using Seven-Nearest Neighbour imputation. Again, we optimized the *C*-parameter within a range of $2^{[-4_\in{\mathbb{Z}}\rightarrow+4]}$.

We assessed the significance of all classifiers via permutation testing^85,86^ with 1000 permutations and α = 0.05. Further, we compared differences between all predictors’ performances in ROP using the nonparametric Quade-test^87^ at the omnibus level followed by post-hoc pairwise comparisons using the t-distribution^88^. Between the ROP and CHR groups we compared the performance of our best predictor (clinical) using the nonparametric and unpaired Wilcoxon rank-sum test, whereas in CHR we compared the best unimodal predictor (clinical) with the best multimodal (clinical-cognitive) predictor. Additionally, we assessed whether our clinical and sMRI-based unimodal predictions were biased by confounding effects such as age, site, sex or level of functioning (Supplementary 4). To assess whether the imbalanced group assignment of the clinical predictor in CHR patients was associated with differences in substance use severity between ROP and CHR groups, we compared the sensitivity and specificity of these models separately for subjects with and without cannabis use disorder (Supplementary 6).

### Feature importance

To understand which features were most reliably contributing to the prediction of CCu, we computed the CV ratio^37,85^. The significance of features for predictors that included wrapper-based feature selection (clinical and cognition) was calculated by sign-based consistency following previous approaches^41^ (Supplementary 5).

### Exploration: effect of continued cannabis use on long-term clinical outcome

To explore the clinical relevance of CCu-prediction, we examined the impact of CCu on long-term clinical outcome employing linear-mixed effects models using the package ‘lmerTest’^89^ in R language for statistical computing, version 3.6.3^90^ separately in ROP and CHR groups. Clinical outcomes, specifically the sum score of positive, negative and general symptoms from the PANSS^91^, the sum score of BDI-II^48^, current symptoms of the GAF-S^46,47^ and current functional disability of the GAF-F until 18 months follow-up entered the model as dependent variables. Following the approach in a previous study^92^ we tested the main fixed effects “group” (CCu vs. DCu), time since baseline, linear, quadratic and cubic trends and trend interactions with the outcome. Patients were modelled as a random effect. We assessed model complexity for both groups (ROP and CHR) and each outcome individually employing the parametric bootstrap method for the Likelihood Ratio Test (*R* package *PBmoDCuomp*^93^) with 200 iterations. We deleted missing data for each case per visit.

## Supplementary information

Supplementary: Pattern of predictive features of continued cannabis use in patients with recent-onset psychosis and clinical high-risk for psychosis

## Data Availability

The data are not publicly available due to Institutional Review Board restrictions—since the participants did not consent to their data being publicly available.

## Code availability

The NeuroMiner software available on github (https://github.com/neurominer-git/NeuroMiner-1) was used for the data analysis.

## References

[1] Stilo SA, Murray RM (2019). Non-genetic factors in schizophrenia. Curr. Psychiatry Rep..

[2] Marconi A, Di Forti M, Lewis CM, Murray RM, Vassos E (2016). Meta-analysis of the association between the level of cannabis use and risk of psychosis. Schizophr. Bull..

[3] Bhattacharyya S (2021). Individualized prediction of 2-year risk of relapse as indexed by psychiatric hospitalization following psychosis onset: Model development in two first episode samples. Schizophr. Res..

[4] Schoeler T (2016). Effects of continuation, frequency, and type of cannabis use on relapse in the first 2 years after onset of psychosis: an observational study. Lancet Psychiatry.

[5] Schoeler T (2016). Continued versus discontinued cannabis use in patients with psychosis: a systematic review and meta-analysis. Lancet Psychiatry.

[6] Bergé D (2016). Predictors of relapse and functioning in first-episode psychosis: a two-year follow-up study. Psychiatr. Services.

[7] Valmaggia LR (2014). Cannabis use and transition to psychosis in people at ultra-high risk. Psychol. Med..

[8] Allsop DJ, Norberg MM, Copeland J, Fu S, Budney AJ (2011). The Cannabis Withdrawal Scale development: patterns and predictors of cannabis withdrawal and distress. Drug Alcohol Depend..

[9] Koskinen J, Löhönen J, Koponen H, Isohanni M, Miettunen J (2010). Rate of cannabis use disorders in clinical samples of patients with schizophrenia: a meta-analysis. Schizophr. Bull..

[10] Budney AJ, Sofis MJ, Borodovsky JT (2019). An update on cannabis use disorder with comment on the impact of policy related to therapeutic and recreational cannabis use. Euro. Arch. Psychiatry Clin. Neurosci..

[11] Babbin SF, Stanger C, Scherer EA, Budney AJ (2016). Identifying treatment response subgroups for adolescent cannabis use. Addict. Behav..

[12] Murray, R. M., Bhavsar, V., Tripoli, G. & Howes, O. 30 years on: how the neurodevelopmental hypothesis of schizophrenia morphed Into the developmental risk factor model of psychosis. *Schizophr. Bull*. **43**, 1190–1196 (2017). 10.1093/schbul/sbx121 PMC5737804 28981842

[13] Choi NG, DiNitto DM, Marti CN (2018). Marijuana use among adults: Initiation, return to use, and continued use versus quitting over a one-year follow-up period. Drug Alcohol Depend..

[14] Ross JM, Pacheco-Colón I, Hawes SW, Gonzalez R (2020). Bidirectional longitudinal associations between cannabis use and body mass index among adolescents. Cannabis Cannabinoid Res..

[15] *Diagnostic and statistical manual of mental disorders. DSM-5* (American Psychiatric Association, Arlington, Va., 2013).

[16] Feingold D, Livne O, Rehm J, Lev-Ran S (2020). Probability and correlates of transition from cannabis use to DSM-5 cannabis use disorder: results from a large-scale nationally representative study. Drug Alcohol Rev..

[17] Flórez-Salamanca L (2013). Probability and predictors of cannabis use disorders relapse: results of the National Epidemiologic Survey on Alcohol and Related Conditions (NESARC). Drug Alcohol Depend..

[18] Lopez-Quintero, C. et al. Probability and predictors of transition from first use to dependence on nicotine, alcohol, cannabis, and cocaine: results of the National Epidemiologic Survey on Alcohol and Related Conditions (NESARC). *Drug Alcohol Depend*. **115**, 120–130 (2011). 10.1016/j.drugalcdep.2010.11.004 PMC3069146 21145178

[19] Allsop DJ (2012). Quantifying the clinical significance of cannabis withdrawal. PLoS ONE.

[20] Hides, L., Dawe, S., Kavanagh, D. J. & Young, R. M. Psychotic symptom and cannabis relapse in recent-onset psychosis: Prospective study. *Br. J. Psychiatry* **189**, 137–143 (2006). 10.1192/bjp.bp.105.014308 16880483

[21] Santacana AM, Pérez-Solá V (2014). Reasons and subjective effects of cannabis use among people with psychotic disorders: a systematic review. Actas Espanolas de Psiquiatria.

[22] Gill KE (2015). Reasons for cannabis use among youths at ultra high risk for psychosis. Early Interv. Psychiatry.

[23] Volkow ND, Koob GF, McLellan AT (2016). Neurobiologic advances from the brain disease model of addiction. N. Engl. J. Med..

[24] Domínguez-Salas S, Díaz-Batanero C, Lozano-Rojas OM, Verdejo-García A (2016). Impact of general cognition and executive function deficits on addiction treatment outcomes: systematic review and discussion of neurocognitive pathways. Neurosci. Biobehav. Rev..

[25] Schoeler T, Kambeitz J, Behlke I, Murray R, Bhattacharyya S (2016). The effects of cannabis on memory function in users with and without a psychotic disorder: findings from a combined meta-analysis. Psychol. Med..

[26] Howes OD, Murray RM (2014). Schizophrenia: an integrated sociodevelopmental-cognitive model. Lancet.

[27] Radua J (2018). What causes psychosis? An umbrella review of risk and protective factors. World Psychiatry.

[28] Heilig M (2021). Addiction as a brain disease revised: why it still matters, and the need for consilience. Neuropsychopharmacology.

[29] Beck A (2012). Effect of brain structure, brain function, and brain connectivity on relapse in alcohol-dependent patients. Arch. Gen. Psychiatry.

[30] Durazzo TC (2011). Cortical thickness, surface area, and volume of the brain reward system in alcohol dependence: relationships to relapse and extended abstinence. Alcoholism Clin. Exp. Res..

[31] Xu J (2014). Hippocampal volume mediates the relationship between measures of pre-treatment cocaine use and within-treatment cocaine abstinence. Drug Alcohol Depend..

[32] Ferland J-MN, Hurd YL (2020). Deconstructing the neurobiology of cannabis use disorder. Nat. Neurosci..

[33] Kroon E, Kuhns L, Hoch E, Cousijn J (2020). Heavy cannabis use, dependence and the brain: a clinical perspective. Addiction (Abingdon, England).

[34] Gupta CN (2015). Patterns of gray matter abnormalities in schizophrenia based on an international mega-analysis. Schizophr. Bull..

[35] Moeller SJ, Paulus MP (2018). Toward biomarkers of the addicted human brain: using neuroimaging to predict relapse and sustained abstinence in substance use disorder. Prog. Neuro Psychopharmacol. Biol. Psychiatry.

[36] Brandl F (2019). Specific substantial dysconnectivity in schizophrenia: a transdiagnostic multimodal meta-analysis of resting-state functional and structural magnetic resonance imaging studies. Biol. Psychiatry.

[37] Koutsouleris N (2018). Prediction models of functional outcomes for individuals in the clinical high-risk state for psychosis or with recent-onset depression: a multimodal, multisite machine learning analysis. JAMA Psychiatry.

[38] Rapp C, Hilal b, Riecher-Rossler A, Tamagni C, Borgwardt S (2012). Effects of cannabis use on human brain structure in psychosis: a systematic review combining in vivo structural neuroimaging and post mortem studies. Curr. Pharm. Des..

[39] Dwyer DB, Falkai P, Koutsouleris N (2018). Machine learning approaches for clinical psychology and psychiatry. Annu. Rev. Clin. Psychol..

[40] Koutsouleris N (2016). Multisite prediction of 4-week and 52-week treatment outcomes in patients with first-episode psychosis: a machine learning approach. Lancet Psychiatry.

[41] Koutsouleris N (2021). Multimodal machine learning workflows for prediction of psychosis in patients with clinical high-risk syndromes and recent-onset depression. JAMA Psychiatry.

[42] Rosen M (2021). Towards clinical application of prediction models for transition to psychosis: a systematic review and external validation study in the PRONIA sample. Neurosci. Biobehav. Rev..

[43] Antonucci LA (2020). A pattern of cognitive deficits stratified for genetic and environmental risk Reliably classifies patients with schizophrenia from healthy control subjects. Biol. Psychiatry.

[44] Kay SR, Fiszbein A, Opler AL (1987). The positive and negative syndrome scale (PANSS) for schizophrenia. Schizophr. Bull..

[45] Mandrekar JN (2010). Receiver operating characteristic curve in diagnostic test assessment. J. Thorac. Oncol..

[46] Jones SH, Thornicroft G, Coffey M, Dunn G (1995). A brief mental health outcome scale-reliability and validity of the Global Assessment of Functioning (GAF)*.*. Br. J. Psychiatry.

[47] Startup M, Jackson MC, Bendix S (2002). The concurrent validity of the Global Assessment of Functioning (GAF). Br. J. Clin. Psychol..

[48] Beck AT, Steer RA, Ball R, Ranieri W (1996). Comparison of Beck Depression Inventories -IA and -II in psychiatric outpatients. J. Personal. Assess..

[49] Brucato G (2017). Baseline demographics, clinical features and predictors of conversion among 200 individuals in a longitudinal prospective psychosis-risk cohort. Psychol. Med..

[50] Cannon TD (2016). An individualized risk calculator for research in prodromal psychosis. Am. J. Psychiatry.

[51] Sanfelici R, Dwyer DB, Antonucci LA, Koutsouleris N (2020). Individualized diagnostic and prognostic models for patients with psychosis risk syndromes: a meta-analytic view on the state of the art. Biol. Psychiatry.

[52] Barrowclough C, Gregg L, Lobban F, Bucci S, Emsley R (2015). The impact of cannabis use on clinical outcomes in recent onset psychosis. Schizophr. Bull..

[53] Chauchard E, Levin KH, Copersino ML, Heishman SJ, Gorelick DA (2013). Motivations to quit cannabis use in an adult non-treatment sample: are they related to relapse?. Addict. Behav..

[54] Fusar-Poli P (2010). Social dysfunction predicts two years clinical outcome in people at ultra high risk for psychosis. J. Psychiatr. Res..

[55] Gullo MJ, Matveeva M, Feeney GFX, Young RM, Connor JP (2017). Social cognitive predictors of treatment outcome in cannabis dependence. Drug Aalcohol Depend..

[56] Spitzer, M. L., Gibbon, R. M. & Williams, J. *Structured clinical interview for DSM-IV-TR axis I disorders, reserach version, non-patient edition (SCID-I/NP)* (Biometricx Research, 2002).

[57] Subbaraman MS (2016). Substitution and complementarity of alcohol and cannabis: a review of the literature. Substance Use Misuse.

[58] Allsop DJ (2014). Changes in cigarette and alcohol use during cannabis abstinence. Drug Alcohol Depend..

[59] McGrath J, Saha S, Chant D, Welham J (2008). Schizophrenia: a concise overview of incidence, prevalence, and mortality. Epidemiol. Rev..

[60] Isvoranu A-M, Borsboom D, van Os J, Guloksuz S (2016). A network approach to environmental impact in psychotic disorder: brief theoretical framework. Schizophr. Bull..

[61] Padmanabhan JL, Shah JL, Tandon N, Keshavan MS (2017). The “polyenviromic risk score”: aggregating environmental risk factors predicts conversion to psychosis in familial high-risk subjects. Schizophr. Res..

[62] Orr C (2019). Grey matter volume differences associated with extremely low levels of cannabis use in adolescence. J. Neurosci..

[63] van Os, J. & Kapur, S. Schizophrenia. *Lancet.* **374**, 635–645, (2009). 10.1016/S0140-6736(09)60995-8 19700006

[64] Volkow ND (2016). Effects of cannabis use on human behavior, including cognition, motivation, and psychosis: a review. JAMA Psychiatry.

[65] Cousijn J (2013). Cannabis dependence, cognitive control and attentional bias for cannabis words. Addict. Behav..

[66] Yücel M (2012). The impact of cannabis use on cognitive functioning in patients with schizophrenia: a meta-analysis of existing findings and new data in a first-episode sample. Schizophr. Bull..

[67] Bourque J, Potvin S (2021). Cannabis and cognitive functioning: from acute to residual effects, from randomized controlled trials to prospective designs. Front. Psychiatry.

[68] Upthegrove R (2021). The psychopathology and neuroanatomical markers of depression in early psychosis. Schizophr. Bull..

[69] González-Ortega I (2015). Subclinical depressive symptoms and continued cannabis use: predictors of negative outcomes in first episode psychosis. PLoS ONE.

[70] Karschner EL, Swortwood-Gates MJ, Huestis MA (2020). Identifying and quantifying cannabinoids in biological matrices in the medical and legal cannabis era. Clin. Chem..

[71] Penzel N (2021). Association between age of cannabis initiation and gray matter covariance networks in recent onset psychosis. Neuropsychopharmacology.

[72] Dilling, H., Mombour, W. & Schmidt, M. H. *Internationale Klassifikation psychischer Störungen. ICD-10 Kapitel V (F): diagnostische Kriterien für Forschung und Praxis*. 6th edn. (Hogrefe, 2016).

[73] Schultze-Lutter, F., Addington, J., Ruhrmann, S. & Klösterkötter, J. *Schizophrenia proneness instrument, adult version (SPI-A)*. (Giovanni Fioriti, 2007).

[74] McGlashan, T., Walsh, B. & Woods, S. *The psychosis-risk syndrome.: Handbook for diagnosis and follow-up*. (Oxford University Press, 2010).

[75] Cornblatt BA (2007). Preliminary findings for two new measures of social and role functioning in the prodromal phase of schizophrenia. Schizophr. Bull..

[76] Endler NS, Parker JD (1990). Multidimensional assessment of coping: a critical evaluation. J. Personal. Soc. Psychol..

[77] Nuechterlein KH (2008). The MATRICS Consensus Cognitive Battery, part 1: test selection, reliability, and validity. Am. J. Psychiatry.

[78] Du Y, Fan Y (2013). Group information guided ICA for fMRI data analysis. NeuroImage.

[79] Gupta CN (2017). Biclustered independent component analysis for complex biomarker and subtype identification from structural magnetic resonance images in schizophrenia. Front. Psychiatry.

[80] Chang C-C, Lin C-J (2011). LIBSVM: a library for support vector machines. ACM Trans. Intell. Syst. Technol..

[81] Saeys Y, Inza I, Larrañaga P (2007). A review of feature selection techniques in bioinformatics. Bioinformatics (Oxford, England).

[82] Mushquash C, O’Connor BP (2006). SPSS and SAS programs for generalizability theory analyses. Behav. Res. Methods.

[83] Fortin J-P (2017). Harmonization of multi-site diffusion tensor imaging data. NeuroImage.

[84] Fortin J-P (2018). Harmonization of cortical thickness measurements across scanners and sites. NeuroImage.

[85] Antonucci LA (2020). Multivariate classification of schizophrenia and its familial risk based on load-dependent attentional control brain functional connectivity. Neuropsychopharmacology.

[86] Golland, P. & Fischl, B. Permutation tests for classification: towards statistical significance in image-based studies. In *Biennial international conference on information processing in medical imaging*, 330–341 (Springer, 2003). 10.1007/978-3-540-45087-0_28 15344469

[87] Quade D (1979). Using weighted rankings in the analysis of complete blocks with additive block effects. J. Am. Stat. Assoc..

[88] Heckert, N. A. & Filliben, J. J. *Dataplot Reference Manual, Volume2: LET Subcommands and Library Functions*. https://www.itl.nist.gov/div898/software/dataplot/document.htm (2003).

[89] Kuznetsova, A., Brockhoff, P. B. & Christensen, R. H. B. lmerTest Package: tests in linear mixed effects models. *J. Stat. Soft*. 10.18637/jss.v082.i13 (2017).

[90] R Core Team. *R: A language and environment for statistical computing*. https://www.R-project.org/ (2020).

[91] Kay S, Fiszbein A, Opler LA (1987). The positive and negative syndrome scale (PANSS) for schizophrenia. Schizophr. Bull..

[92] Dwyer DB (2020). An investigation of psychosis subgroups with prognostic validation and exploration of genetic underpinnings: the PsyCourse study. JAMA Psychiatry.

[93] Halekoh, U. & Højsgaard, S. A Kenward-roger approximation and parametric bootstrap methods for tests in linear mixed models - the R package pbkrtest. *J. Stat. Soft*. **59**, 10.18637/jss.v059.i09 (2014).

