# Supplementary material for: Pattern of predictive features of continued cannabis use in patients with recent-onset psychosis and clinical high-risk for psychosis
Source: Schizophrenia (Heidelb). 2022 Mar 9;8(1):19. doi: 10.1038/s41537-022-00218-y (PMC8907166; doi:10.1038/s41537-022-00218-y)
Supplement: Supplementary file 1 — Supplementary: Pattern of predictive features of continued cannabis use in patients with recent-onset psychosis and clinical high-risk for psychosis [file 41537_2022_218_MOESM1_ESM.docx]

**Supplementary: Pattern of predictive features of continued cannabis use in patients with recent-onset psychosis and clinical high-risk for psychosis**

Nora Penzel, MSc^1,2,3^, Rachele Sanfelici, MSc^2,4^, Linda A. Antonucci, PhD^2,5^, Linda T. Betz, MSc^1^, Dominic Dwyer, PhD^2^, Anne Ruef, MSc^2^, Kang Ik Kevin Cho, PhD^6^, Paul Cumming, PhD^7,8,9^, Oliver Pogarell, MD^2^, Oliver Howes, PhD^10,11,12,13^, Peter Falkai, MD^2,4^, Rachel Upthegrove, MBBS FRCPsych, PhD^14,15^, Stefan Borgwardt, MD^16,17^, Paolo Brambilla, MD^18,19^, Rebekka Lencer, MD^17,20,21^, Eva Meisenzahl, MD^22^, Frauke Schultze-Lutter, PhD^22,23,24^, Marlene Rosen, PhD^1^, Theresa Lichtenstein, MD^1^, Lana Kambeitz-Ilankovic, PhD^1,2^, Stephan Ruhrmann, MD^1^, Raimo R. K. Salokangas, PhD, MD^25^, Christos Pantelis, PhD, MD^26^, Stephen J. Wood, PhD^14,27,28^, Boris B. Quednow, PhD^29^, Giulio Pergola, PhD^3^, Alessandro Bertolino, PhD, MD^3^, Nikolaos Koutsouleris, MD^2,4,30^, Joseph Kambeitz, MD^1*^ and the PRONIA Consortium

1 University of Cologne, Faculty of Medicine and University Hospital Cologne, Department of Psychiatry and Psychotherapy, Cologne, Germany

2 Department of Psychiatry and Psychotherapy, Ludwig-Maximilian-University, Munich, Germany

3 Group of Psychiatric Neuroscience, Department of Basic Medical Sciences, Neuroscience and Sense Organs, University of Bari 'Aldo Moro', Bari, Italy

4 Max-Planck Institute of Psychiatry, Munich, Germany

5 Department of Education, Psychology, Communication, University of Bari, Bari, Italy

6 Department of Psychiatry, Brigham and Women’s Hospital, Harvard Medical School, Boston, MA, USA

7 Department of Nuclear Medicine, Bern University Hospital, Bern, Switzerland

8 School of Psychology and Counselling, Queensland University of Technology, Brisbane, Australia

9 International Research Lab in Neuropsychiatry, Neuroscience Research Institute, Samara State Medical University, Samara, Russia

10 Department of Psychosis Studies, Institute of Psychiatry, Psychology & Neuroscience, King’s College London, De Crespigny Park, London, SE5 8AF, UK

11 MRC London Institute of Medical Sciences, Hammersmith Hospital, London, W12 0NN, UK

12 Institute of Clinical Sciences, Faculty of Medicine, Imperial College London, London, W12 0NN, UK

13 South London and Maudsley NHS Foundation Trust, London, SE5 8AF, UK

14 Institute for Mental Health, University of Birmingham, Birmingham, United Kingdom

15 Early Intervention Service, Birmingham Womens and Childrens NHS Foundation Trust

16 Department of Psychiatry (UPK), University of Basel, Basel, Switzerland

17 Department of Psychiatry and Psychotherapy, University of Lübeck, Germany

18 Department of Neurosciences and Mental Health, Fondazione IRCCUS Ca' Granda Ospedale Maggiore Policlinico, University of Milan, Milan, Italy

19 Department of Pathophysiology and Transplantation, University of Milan, Milan, Italy

20 Department of Psychiatry and Psychotherapy, University of Münster, Münster, Germany

21 Otto Creutzfeldt Center for Behavioral and Cognitive Neuroscience, University of Münster, Münster, Germany

22 Department of Psychiatry and Psychotherapy, Medical Faculty, Heinrich-Heine University, Düsseldorf, Germany

23 Department of Psychology, Faculty of Psychology, Airlangga University, Surabaya, Indonesia

24 University Hospital of Child and Adolescent Psychiatry and Psychotherapy, University of Bern, Bern, Switzerland

25 Department of Psychiatry, University of Turku, Turku, Finland

26 Melbourne Neuropsychiatry Centre, University of Melbourne & Melbourne Health, Australia

27 Orygen, Melbourne, Australia,

28 Centre for Youth Mental Health, University of Melbourne, Melbourne, Australia

29 Experimental and Clinical Pharmacopsychology, Department of Psychiatry, Psychotherapy, and Psychosomatics, Psychiatric Hospital of the University of Zurich, Lenggstr. 31, 8032 Zurich, Switzerland

30 Institute of Psychiatry, Psychology & Neuroscience, Department of Psychosis Studies, King’s College London, London, United Kingdom

**Corresponding author:**

Joseph Kambeitz

Professor for Biological Psychiatry

Department of Psychiatry and Psychotherapy

Universitätsklinikum Köln, Kerpenerstraße 62

D-50937 Köln, Germany

Tel: 0049-(0)-221-478-4024

Fax: 0049-(0)-221-478-87139

[joseph.kambeitz@uk-koeln.de](mailto:joseph.kambeitz@uk-koeln.de)

Content

[1.) Exclusion criteria for patients with recent-onset psychosis and clinical high-risk for psychosis 5](#_Toc92748024)

[2.) Acquisition and pre-processing of structural magnetic resonance imaging 5](#_Toc92748025)

[3.) Design of Machine learning analysis performed on structural magnetic resonance imaging 6](#_Toc92748026)

[3.1 Healthy individuals used for harmonization between sites and regression of age- and sex specific effects 6](#_Toc92748027)

[3.2 G-theory based mask correction for site-specific variation 6](#_Toc92748028)

[3.3 Harmonization between sites using the ComBat-algorithm 7](#_Toc92748029)

[3.4 Machine learning pipeline of structural magnetic resonance imaging predictor 8](#_Toc92748030)

[4.) Assessment of potential confounds in the clinical and structural magnetic resonance imaging predictors 9](#_Toc92748031)

[5.) Visualization of feature importance 9](#_Toc92748032)

[6.) Investigation of different results in patients with SCID-IV diagnoses of cannabis use disorder 10](#_Toc92748033)

[7.) Supplementary Tables 12](#_Toc92748034)

[sTable 1 Assessments of the cannabis induced psychosis and the ‘Personalized Prognostic Tools for Early Psychosis Management’ studies - Table from ^2^, originally adapted from ^5^ 12](#_Toc92748035)

[sTable 2 Features of clinical and cognitive predictors 15](#_Toc92748036)

[sTable 3 sMRI protocol per Site 21](#_Toc92748037)

[sTable 4 Healthy individuals used for harmonization between sites and regression of age- and sex specific effects 21](#_Toc92748038)

[sTable 5 Association between component-loadings derived with group information-guided independent component analysis and site before and after ComBat-harmonization in healthy individuals 22](#_Toc92748039)

[sTable 6 Associations between site and component-loadings derived with group information-guided independent component analysis before and after application of ComBat-harmonization estimates learned in healthy individuals applied to patients with recent-onset psychosis and clinical high-risk for psychosis patients 22](#_Toc92748040)

[sTable 7 Matched recent-onset psychosis sample based on age, sex, site and scores of the Global Assessment of Functioning (functional disability) to the original sample 23](#_Toc92748041)

[sTable 8 Differences between patients with continued and discontinued cannabis use 24](#_Toc92748042)

[sTable 9 Prediction results in recent-onset psychosis matched based on age, sex, site and scores from the Global Assessment of Functioning (functional disability) to original sample 27](#_Toc92748043)

[sTable 10 Results of Mixed-model analysis of illness course 28](#_Toc92748044)

[sTable 11 Results of the clinical predictor for patients with and without cannabis use disorder separately 29](#_Toc92748045)

[8.) Supplementary Figures 30](#_Toc92748046)

[sFigure 1 Substance Use Questionnaire 30](#_Toc92748047)

[sFigure 2 Machine learning pipeline of structural Magnetic Resonance Imaging (sMRI) 31](#_Toc92748048)

[sFigure 3 Age and sex distribution of healthy individuals used for harmonization between sites and regression of age- and sex specific effects 32](#_Toc92748049)

[sFigure 4 Components derived by group information-guided independent component analysis in healthy individuals used for harmonization between sites and regression of age- and sex specific effects before and after ComBat-harmonization 33](#_Toc92748050)

[sFigure 5 Effect sizes between patients with continued/discontinued cannabis use in recent-onset psychosis before and after harmonization 34](#_Toc92748051)

[sFigure 6 Effect sizes between patients with continued/discontinued cannabis use in clinical high-risk for psychosis before and after harmonization 35](#_Toc92748052)

[sFigure 7 Flow Diagram of inclusion/exclusion for the three different predictors 36](#_Toc92748053)

[sFigure 8 Comparison between different predictor performances 37](#_Toc92748054)

[References 38](#_Toc92748055)

# 1.) Exclusion criteria for patients with recent-onset psychosis and clinical high-risk for psychosis

Patients with recent-onset psychosis (ROP) were excluded if they had taken antipsychotic medication for more than 90 cumulative days, at or above the minimum dosage indicated for first episode psychosis as specified in the DGPPN S3 Guidelines (guideline manual is available in

<https://www.dgppn.de/_Resources/Persistent/43ca38d4b003b8150b856df48211df68e412d9c9/038-009k_S3_Schizophrenie_2019-03.pdf>). Patients with CHR were excluded if they had taken antipsychotic medication for more than 30 cumulative days or if they had received antipsychotic medication at the minimum recommended dosage for first episode psychosis in the past three months prior to baseline assessment.

Further exclusion criteria were any traumatic head injury with loss of consciousness for more than five minutes, any contraindication for magnetic resonance imaging (MRI), any neurological or somatic disease affecting the brain, a lifetime diagnosis of alcohol dependence, current or within-past-six-months polysubstance dependence or inadequate language proficiency in English or the national language at the respective site.

# 2.) Acquisition and pre-processing of structural magnetic resonance imaging

Data was acquired with isotropic or nearly isotropic voxel size, with a preferred voxel size of one mm^3^. The parameters of the field of view had to ensure full 3D coverage of the entire brain including the cerebellum, and other imaging parameters had to maximize the contrast between white matter and cortical ribbon as well as obtaining an optimal signal-to-noise ratio.

For pre-processing, we used the open-source CAT12 toolbox (version r1155;<http://dbm.neuro.uni-jena.de/cat12/>), an extension of SPM12 running in MATLAB 2018a. As a first step, all images were segmented into grey matter (GM), white matter and cerebrospinal fluid maps and normalized to stereotactic space of Montreal Neurological Institute (MNI-152 space). To derive GM volume maps, images were multiplied with the Jacobian determinants obtained during registration. *Post-hoc* quality checks were performed by correlating each slice across all subjects. Four scans from patients with ROP and one scan from a CHR patient deviated by more than two standard deviations (SD) of the mean, and were consequently re-examined visually. Due to artifacts, one scan from a patient with ROP and one image from a patient with CHR had to be excluded from the subsequent analysis, whereas the other images passed inspection (sFigure 6). Subsequently, images were realigned to a two mm voxel resolution and smoothed with a ten mm (full-width at half maximum) Gaussian kernel ^1^.

# 3.) Design of Machine learning analysis performed on structural magnetic resonance imaging

## 3.1 Healthy individuals used for harmonization between sites and regression of age- and sex specific effects

To build a reliable classifier of continued cannabis use (CCu) based on structural magnetic resonance imaging (sMRI) data, we had first to remove the effect of several well-known confounders, i.e., age-, sex- and site-specific variation. To remove variation related to differences between these factors and simultaneously preserve the effect of interest (CCu) we used a subset of the healthy individuals (HC) from the ‘Personalized Prognostic Tools for Early Psychosis Management’ study (PRONIA) to define the age, sex, and site-specific variation and then remove these effects individually from our patient groups (ROP, CHR) individually. HC from the seven sites included in the sMRI classifier were matched for age and sex between sites (see sFigure 3 for age and sex distribution by site, statistics in sTable 4) and used as a target for harmonization.

## 3.2 G-theory based mask correction for site-specific variation

As a first step, following previous publications from our group we used the so-called g-theory based mask to correct for site-specific scanner variation ^2–4^. In short, MRI data from six individuals, who agreed to be scanned at six of the seven included PRONIA sites (Munich, Milan Niguarda, Basel, Cologne, Birmingham, Turku), were analysed voxel-wise for subject- and site-specific variation. Here, higher g-values indicate high subject- and low site-specific variation, and low g-values indicate high site- and low-subject specific variation ^5^. The reference components (RCs) and all images from the patient groups and HCs used for harmonization between sites and regression of age- and sex specific effects, were thresholded with the g-mask (voxels>0) using SPM12 running in MATLAB 2020 (sFigure 2) to exclude voxels showing only between-site but no inter-subject-variation ^2,6^.

## 3.3 Harmonization between sites using the ComBat-algorithm

Second, we applied on the remaining voxels the ComBat algorithm ^7^, which is a harmonization method removing non-biological variation related to differences between MRI scanners. To preserve the biological variation of interest (predicting CCu), we trained the ComBat algorithm on our matched HCs. Before applying the learned rule from the ComBat-algorithm trained on HCs to the patient data we first compared the uncorrected sMRI data with the corrected data. Here, we used group information-guided independent components analysis (GIG-ICA) pre- and post-harmonization to find nine components of interest (COIs) in HCs that were optimized for similarity with our nine RCs and for independence between each other. Then, we tested for site-specific effects on the COIs by conducting an analysis of variance with loadings serving as the independent factor, and site as a dummy-coded variable serving as the dependent factor. Further, we investigated the pattern of sex- and age-specific variation in the COIs pre- and post-harmonization by visualizing their pattern (sFigure 3). Pronounced site-specific effects that were present pre-harmonization were removed post-harmonization in all COIs while preserving age- and sex-specific variations (see sTable 5, sFigure 3).

We then applied our model to the discovery (ROP) and replication sample (CHR) independently, and retested whether site-specific variation had indeed been removed from the COIs. This time we investigated whether the biological signature of interest was preserved despite the harmonization process. To this end, we calculated the effect sizes of CCu on COIs pre- and post- harmonization as Cohen’s d. Even though some significant site-specific variation remained the effects were reduced (sTable5), while the effect sizes of the effect of interest (CCu) in all COIs and sites were preserved (sFigure 6).

## 3.4 Machine learning pipeline of structural magnetic resonance imaging predictor

Finally, the thresholded and site-corrected sMRI images were entered into our machine learning pipeline. We first corrected for sex and age effects using betas computed in our HC subsample. To strictly separate training from testing, we implemented GIG-ICA from the GIFT toolbox (<http://mialab.mrn.org/software/gift/>) in NeuroMiner to reduce dimensionality. GIG-ICA was optimized to identify nine independent components with maximum similarity to nine RCs that had previously been associated with schizophrenia ^1^. In each inner cross-validation (CV)-fold, GM images were converted to one-dimensional row vectors and concatenated across patients deriving a N_patients_-by-205,075 grey matter volume voxel matrix. This matrix was decomposed into a source matrix and a mixing matrix. The mixing matrix represents loadings, i.e., the weights of individual patients on each COI. The source matrix represents the relationship between each voxel and the COI. This decomposition simultaneously maximizes the correspondence to the RCs and the independence of the components from each other such that each row in the source matrix is maximally independent from the others. Next, the components were scaled between zero and one. As in the other modalities, we employed a linear support vector machine (SVM) ^8^ with an optimization of the C-parameter within a range from 2^[−4∈ℤ→ +4] 3^. For a graphical depiction of the entire sMRI machine learning pipeline see sFigure 3.

# 4.) Assessment of potential confounds in the clinical and structural magnetic resonance imaging predictors

We are aware that several well-known confounding factors might have biased our predictive accuracies in the clinical- and sMRI-predictors. For sMRI, site-, age- and sex-effects might be particularly relevant. Further, level of functioning was associated with CCu in follow-up (see “Results Section” of the main manuscript Figure 1) and was one of the most important patterns of our clinical predictor of CCu (see “Results Section” of the main manuscript Figure 2). Therefore, we performed a validation analysis to explore the specificity of our prediction for CCu. We used patients with ROP (ROP-artificial) that had been excluded from the main study due to missing cannabis information (N = 73) or non-user status at baseline (N = 36). We assigned artificial “continued cannabis use (CCu)” and “discontinued cannabis use (DCu)” labels to the ROP-artificial patients. Group membership (CCu/DCu) was defined by matching subjects for age-, sex-, site- and highest functioning in past month measured with the Global Assessment of Functioning scale to our original ROP patients (see sTable 7). Then, we used the identical machine learning pipeline for the clinical- and sMRI predictor that we had employed in the main part of the study for the prediction of CCu. Results of this prediction are presented in sTable 8. Both predictors provided non-significant predictions, leading us to conclude that our results are unlikely to be biased by age, sex, site, or functioning.

# 5.) Visualization of feature importance

To understand which features were most reliably contributing to the prediction of CCu, we computed the CV ratio $(CVR=\frac{mean(w)}{{SE}_{(w)}})$ ^4,9^. Here, $w$ represents the normalized weights under Euclidian assumptions of the linear SVM generated in our repeated nested CV scheme. A positive $CVR$ indicates that an increase of a feature predicts CCu, while a negative $CVR$ means that any decrease in the respective feature predicts CCu. Significance of features for predictors that included wrapper-based feature selection (clinical and cognition) was calculated by sign-based consistency

${(I}_{j}=\frac{|\sum_{i=1}^{n} \hat{w}_{j}^{i}>0-\sum_{i=1}^{n} \hat{w}_{j}^{i}<0 |}{n}*(1-\frac{|\sum_{i=1}^{n} \hat{w}_{j}^{i}=0}{n})$ ^3^.

Here, $I_{j}$ represents the importance of the $j^{th}$ feature and $\hat{w}_{j}^{i}$ represents the normalized weights under Euclidian assumptions of the linear SVM for the $j^{th}$ feature. In the first term of the equation (left-hand side), the consistency of the weights assigned by SVM to given features are calculated. This consistency is reduced by the second term of the equation (right-hand side), which measures the fraction of SVMs that de-selected the particular feature. Significance threshold was defined based on z-statistic, defined as ${(z}_{j}=\frac{I_{j}}{\sqrt{var\{I_{j}\}}})$ and p-values were corrected for the false-discovery rate (FDR) following previous approaches^3^.

# 6.) Investigation of different results in patients with SCID-IV diagnoses of cannabis use disorder

ROP patients had more lifetime cannabis use disorder than patients with CHR. As substance use patterns were an important predictor for CCu and patients with CHR were more likely classified as having DCu (low sensitivity of the predictor) we further investigated whether the sensitivity and specificity differed between individuals with cannabis use disorder at baseline. Thus, we calculated the overall sensitivity and specificity as well as for the individual median for the five permutations of the outer CV2-folds for patients with and without cannabis use disorder in ROP and CHR patients, separately. We then employed unpaired two-sample Wilcoxon-rank-sum test to compare sensitivity and specificity between patients with cannabis use disorder and without for ROP and CHR patients. The metrics differed significantly in both patient groups (sTable 11). Therefore, the moderate generalizability of the classifier might at least partially be explained by the differences in severity of substance use between ROP and CHR patients.

# 7.) Supplementary Tables

## sTable 1 Assessments of the cannabis induced psychosis and the ‘Personalized Prognostic Tools for Early Psychosis Management’ studies - Table from ^2^, originally adapted from ^5^

| **Instrument** | **Form** | **Baseline** |  | **IV3** | **IV6** | **T1** |  | **IV12** | **IV15** | **T2** |
| --- | --- | --- | --- | --- | --- | --- | --- | --- | --- | --- |
|  |  | **ROP** | **CIP** | **ROP** | **ROP** | **ROP** | **CIP** | **ROP** | **ROP** | **ROP** |
| **General data** | **OR** | **X** | **X** |  |  | **X** | **X** |  |  |  |
| **Reasons for referral** | **OR** | **X** | **X** |  |  |  |  |  |  |  |
| **Treatment documentation** | **OR** | **X** | **X** | **X** | **X** | **X** | **X** | **X** | **X** | **X** |
| **Somatic state and health history** | **OR** | **X** | **X** |  |  | **X** | **X** |  |  | **X** |
| **SPI-A COGDIS/ COPER^10^** | **OR** | **X** |  | **X** | **X** | **X** |  | **X** | **X** | **X** |
| **SIPS positive symptoms^11^** | **OR** | **X** | **X** | **X** | **X** | **X** | **X** | **X** | **X** | **X** |
| **CAARMS^12^** | **OR** | **X** | **X** | **X** | **X** | **X** | **X** | **X** | **X** | **X** |
| **GAF^13^** | **OR** | **X** | **X** | **X** | **X** | **X** | **X** | **X** | **X** | **X** |
| **UHR - Schizotypy, genetic risk** | **OR** | **X** | **X** | **X** |  | **X** | **X** |  |  | **X** |
| **CHR criteria** | **OR** | **X** |  |  |  | **X** |  |  |  | **X** |
| **SCID-IV screening^14^** | **OR** | **X** | **X** |  |  | **X** | **X** |  |  | **X** |
| **SCID-IV summary^14^** | **OR** | **X** | **X** |  |  | **X** | **X** |  |  | **X** |
| **Demographic and biographic data** | **OR** | **X** | **X** |  |  | **X** | **X** |  |  | **X** |
| **PAS^15^** | **OR** | **X** | **X** |  |  | **X** | **X** |  |  | **X** |
| **SPI-A^10^** | **OR** | **X** |  |  |  | **X** |  | **X** | **X** | **X** |
| **SIPS negative, disorganized and general symptoms^11^** | **OR** | **X** | **X** |  |  | **X** | **X** |  |  | **X** |
| **PANSS^16^** | **OR** | **X** | **X** | **X** | **X** | **X** | **X** | **X** | **X** | **X** |
| **SANS^17^** | **OR** | **X** | **X** |  |  | **X** | **X** |  |  | **X** |
| **Chart of life events** | **OR** | **X** | **X** | **X** | **X** | **X** | **X** | **X** | **X** | **X** |
| **FROGS^18^** | **OR** | **X** | **X** |  |  | **X** | **X** |  |  | **X** |
| **GF: Social & role^19^** | **OR** | **X** | **X** | **X** | **X** | **X** | **X** | **X** | **X** | **X** |
| **Prognostic evaluation** | **OR** | **X** | **X** |  |  | **X** | **X** |  |  | **X** |
| **Substance use questionnaire** | **OR** | **X** | **X** | **X** | **X** | **X** | **X** | **X** | **X** | **X** |
| **MSPSS^20^** | **SR** | **X** | **X** |  |  | **X** | **X** |  |  | **X** |
| **RSA^21^** | **SR** | **X** | **X** |  |  | **X** | **X** |  |  | **X** |
| **CISS 24^22^** | **SR** | **X** | **X** |  |  | **X** | **X** |  |  | **X** |
| **SPIN^23^** | **SR** | **X** | **X** |  |  | **X** | **X** |  |  | **X** |
| **BDI-II^24^** | **SR** | **X** | **X** | **X** | **X** | **X** | **X** | **X** | **X** | **X** |
| **WHO-QOL-BREF^25^** | **SR** | **X** | **X** |  |  | **X** | **X** |  |  | **X** |
| **EHI-SR^26^** | **SR** | **X** | **X** |  |  |  |  |  |  |  |
| **LEE^27^** | **SR** | **X** | **X** |  |  | **X** | **X** |  |  | **X** |
| **Wisconsin scales^28^** | **SR** | **X** | **X** |  |  |  |  |  |  |  |
| **EDS^29^** | **SR** | **X** | **X** |  |  |  |  |  |  |  |
| **Bullying scale ^30^** | **SR** | **X** | **X** |  |  |  |  |  |  |  |
| **CTQ^31^** | **SR** | **X** | **X** |  |  |  |  |  |  |  |
| **NEO-FFI^32^** | **SR** | **X** | **X** |  |  |  |  |  |  |  |
| **Substance use** | **SR** |  | **X** |  |  |  |  |  |  |  |
| **Cannabis experience questionnaire (CEQ)** | **SR** |  | **X** |  |  |  |  |  |  |  |
| **Severity of dependency scale (SES)** | **SR** |  | **X** |  |  |  |  |  |  |  |
| **DS backward (BACS)** | **NPT** | **X** | **X** |  |  | **X** |  |  |  |  |
| **DS forward (BACS)** | **NPT** | **X** | **X** |  |  | **X** |  |  |  |  |
| **CPT-IP (BACS)^33^** | **NPT** | **X** | **X** |  |  | **X** |  |  |  |  |
| **DANVA^34^** | **NPT** | **X** | **X** |  |  | **X** |  |  |  |  |
| **DSST** | **NPT** | **X** | **X** |  |  | **X** |  |  |  |  |
| **RAVLT*^35^** | **NPT** | **X** | **X** |  |  | **X** |  |  |  |  |
| **ROCF^36^** | **NPT** | **X** | **X** |  |  | **X** |  |  |  |  |
| **SAT^37^** | **NPT** | **X** | **X** |  |  | **X** |  |  |  |  |
| **SOPT^38^** | **NPT** | **X** | **X** |  |  | **X** |  |  |  |  |
| **TMT-A^39^** | **NPT** | **X** | **X** |  |  | **X** |  |  |  |  |
| **TMT-B^39^** | **NPT** | **X** | **X** |  |  | **X** |  |  |  |  |
| **VF phonetic** | **NPT** | **X** | **X** |  |  | **X** |  |  |  |  |
| **VF semantic** | **NPT** | **X** | **X** |  |  | **X** |  |  |  |  |
| **WAIS-III^40^** | **NPT** | **X** | **X** |  |  | **X** |  |  |  |  |
| **sMRI** | **MRI** | **X** | **X** |  |  | **X** |  |  |  |  |
| **rs-fMRI** | **MRI** | **X** | **X** |  |  | **X** |  |  |  |  |
| **DWI** | **MRI** | **X** | **X** |  |  | **X** |  |  |  |  |
| **blood sample** | **bio** | **X** |  |  |  | **X** |  |  |  |  |
| **hair sample** | **THC** |  | **X** |  |  |  |  |  |  |  |
| **Urine sample** | **THC** |  | **X** |  |  |  |  |  |  |  |
| **EEG** | **EEG** |  | **X** |  |  |  |  |  |  |  |

*Abbreviation:* IV3 = interval three months after baseline, IV6 = interval six months after baseline, T1 = interval nine months after baseline, IV12 = interval 12 months after baseline, IV15 = interval 15 months after baseline, T2 = interval 18 months after baseline, OR = Observer-based-rating instrument, SR = Self-rating-based instrument, NPT = Neuropsychological Test, MRI = Magnetic Resonance Imaging, sMRI = structural Magnetic Resonance Imaging, rs-fMRI = resting-state functional Magnetic Resonance Imaging, DWI = Diffusion Weighted Imaging, bio = biological test, THC = Cannabis-related test, EEG = Electro Encephalography, SPI-A COGDIS/COPER = Schizophrenia Proneness Instrument - Cognitive disturbances / Cognitive-Perceptual disturbances, CAARMS = Comprehensive Assessment of the At-Risk Mental States, CHR Criteria, SIPS = Standardized Interview for the assessment of Prodromal Symptoms (modified version 5.0), GAF = Global Assessment of Functioning, UHR – Schizotypy, genetic risk = Genetic Risk Interview for the Assessment of Schizotypal personality traits, and familial risk for psychosis, CHR criteria = Clinical High-Risk criteria summary questionnaire, SCID-IV Screening/Summary = Structured Clinical Interview for DSM-IV, PAS = Premorbid Adjustment Scale, PANSS = Positive and Negative Syndrome Scale, SANS = Scale for the Assessment of Negative Symptoms, FROGS = Functional Remission in General Schizophrenia, GF: Social/Role = Global Functioning: Social/Role, MSPSS = the Multidimensional Scale for Perceived Social Support, RSA = Resilience Scale for Adults, CISS 24 = Coping Inventory for Stressful Situations – 24 items, SPIN = Social Phobia Inventory, BDI-II = Beck Depression Inventory II, WHO-QOL-BREF = WHO Quality of Life Questionnaire – Brief Version, EHI-SR = Edinburgh Handedness Inventory – Short Version, LEE = Level of Expressed Emotions, Wisconsin scales = , EDS = Everyday Discrimination Scale – Modified Version, CTQ = Childhood Trauma Questionnaire, NEO-FFI = NEO Five Factor Inventory of Personality Traits, DS = Auditory Digit Span (Forward/Backward) adapted from the PEBL battery, CPT-IP (BACS) = Continuous-Performance Test – Identical Pairs (adapted tablet version), DANVA = Diagnostic Analysis of

Non-Verbal Accuracy 2 (adapted tablet version), DSST = Digit-Symbol-Substitution Test from the BACS battery, RAVLT = Rey Auditory Verbal test, ROCF = Rey-Osterrieth complex figure, SAT = Salience Attribution Task (adapted version), SOPT = self-ordered pointing task (adapted version), TMT-A/-B = Trail-Making Test A and B, VF phonetic/semantic = verbal fluency test, WAIS-III = Wechsler Adult Intelligence Scale (3^rd^ edition), ROP = recent-onset psychosis via the ‘Personalized Prognostic Tools for Early Psychosis Management’, CIP = recent-onset psychosis patients included via the cannabis induced psychosis study, CHR = clinical high-risk for psychosis

***In one of the research sites (Turku) the revised version of the Hopkins Verbal Learning Test (HVLT-R) was included instead of the RAVLT that was not available in Finnish.**

## sTable 2 Features of clinical and cognitive predictors

| **Category** | **Feature** | **Percent missing in Discovery sample (ROP)** | **Percent missing in Replication sample (CHR)** |
| --- | --- | --- | --- |
| Substance Use related items | Number of Other Substances besides Cannabis tried in Lifetime | 22.0 | 24.7 |
| Substance Use related items | DSM-IV Lifetime Diagnosis of Cannabis Abuse | 0.0 | 1.4 |
| Substance Use related items | DSM-IV Lifetime Diagnosis of Cannabis Dependency | 0.0 | 1.4 |
| Substance Use related items | Average Number of cigarettes per day | 7.3 | 2.7 |
| Substance Use related items | Average Units of alcohol consumed per day | 22.9 | 23.3 |
| Substance Use related items | Cumulative Frequency of Cannabis used in the last 3 months | 26.6 | 26 |
| Substance Use related items | Cumulative Frequency of Other Substances besides Cannabis used in the last 3 months | 39.4 | 53.4 |
| Substance Use related items | Age of cannabis use initiation | 10.1 | 20.5 |
| Substance Use related items | Cumulative time of Cannabis Use in Lifetime (months) | 14.7 | 20.5 |
| Substance Use related items | Time since last Cannabis Use before Baseline (months) | 10.1 | 13.7 |
| Environmental risk factors | Sum of experienced burden of Recent Life Events (last year) | 2.8 | 0.0 |
| Environmental risk factors | CTQ (emotional abuse) | 14.7 | 6.8 |
| Environmental risk factors | CTQ (emotional neglect) | 13.8 | 5.5 |
| Environmental risk factors | CTQ (physical abuse) | 12.8 | 6.8 |
| Environmental risk factors | CTQ (physical neglect) | 13.8 | 8.2 |
| Environmental risk factors | CTQ (sexual abuse) | 15.6 | 8.2 |
| Environmental risk factors | WSS (magical ideation) | 15.6 | 11.0 |
| Environmental risk factors | WSS (perceptual aberration) | 12.8 | 8.2 |
| Environmental risk factors | WSS (physical anhedonia) | 16.5 | 8.2 |
| Environmental risk factors | WSS (social anhedonia) | 16.5 | 9.6 |
| Symptoms | BDI-II 1 – Sadness | 8.3 | 5.5 |
| Symptoms | BDI-II 2 - Pessimism | 9.2 | 4.1 |
| Symptoms | BDI-II 3 – Past failures | 9.2 | 4.1 |
| Symptoms | BDI-II 4 – Loss of Pleasure | 9.2 | 4.1 |
| Symptoms | BDI-II 5 – Guilt Feelings | 9.2 | 4.1 |
| Symptoms | BDI-II 6 – Punishment Feelings | 9.2 | 4.1 |
| Symptoms | BDI-II 7 – Self Dislike | 9.2 | 4.1 |
| Symptoms | BDI-II 8 – Self Criticalness | 9.2 | 4.1 |
| Symptoms | BDI-II 9 – Suicidal thoughts or wishes | 9.2 | 4.1 |
| Symptoms | BDI-II 10 – Crying | 9.2 | 5.5 |
| Symptoms | BDI-II 11 – Agitation | 9.2 | 4.1 |
| Symptoms | BDI-II 12 – Loss of Interest | 9.2 | 5.5 |
| Symptoms | BDI-II 13 – Indecisiveness | 9.2 | 4.1 |
| Symptoms | BDI-II 14 – Worthlessness | 9.2 | 4.1 |
| Symptoms | BDI-II 15 – Loss of Energy | 9.2 | 4.1 |
| Symptoms | BDI-II 16 – Changes in Sleeping Pattern | 9.2 | 5.5 |
| Symptoms | BDI-II 17 – Irritability | 9.2 | 4.1 |
| Symptoms | BDI-II 18 – Changes in Appetite | 9.2 | 5.5 |
| Symptoms | BDI-II 19 – Concentration Difficulty | 9.2 | 5.5 |
| Symptoms | BDI-II 20 – Tiredness or Fatigue | 9.2 | 5.5 |
| Symptoms | BDI-II 21 – Loss of Interest in Sex | 11 | 4.1 |
| Symptoms | PANSS G1 – Somatic concern | 1.8 | 4.1 |
| Symptoms | PANSS G2 – Anxiety | 1.8 | 4.1 |
| Symptoms | PANSS G3 – Guilt feelings | 1.8 | 4.1 |
| Symptoms | PANSS G4 – Tension | 1.8 | 4.1 |
| Symptoms | PANSS G5 – Mannerisms and posturing | 1.8 | 4.1 |
| Symptoms | PANSS G6 – Depression | 1.8 | 4.1 |
| Symptoms | PANSS G7 – Motor retardation | 1.8 | 4.1 |
| Symptoms | PANSS G8 – Uncooperativeness | 1.8 | 4.1 |
| Symptoms | PANSS G9 – Unusual thought content | 1.8 | 4.1 |
| Symptoms | PANSS G10 – Disorientation | 1.8 | 4.1 |
| Symptoms | PANSS G11 – Poor attention | 1.8 | 4.1 |
| Symptoms | PANSS G12 – Lack of judgement and insight | 1.8 | 4.1 |
| Symptoms | PANSS G13 – Disturbance of volition | 1.8 | 4.1 |
| Symptoms | PANSS G14 – Poor impulse control | 1.8 | 4.1 |
| Symptoms | PANSS G15 – Preoccupation | 1.8 | 4.1 |
| Symptoms | PANSS G16 – Active social avoidance | 1.8 | 4.1 |
| Symptoms | PANSS N1 – Blunted affect | 1.8 | 4.1 |
| Symptoms | PANSS N2 – Emotional withdrawal | 1.8 | 4.1 |
| Symptoms | PANSS N3 – Poor Rapport | 1.8 | 4.1 |
| Symptoms | PANSS N4 – Passive/apathetic social withdrawal | 1.8 | 4.1 |
| Symptoms | PANSS N5 – Difficulty in abstract thinking | 1.8 | 4.1 |
| Symptoms | PANSS N6 – Lack of spontaneity and flow of conversation | 1.8 | 4.1 |
| Symptoms | PANSS N7 – Stereotyped thinking | 1.8 | 4.1 |
| Symptoms | PANSS P1 – Delusions | 1.8 | 4.1 |
| Symptoms | PANSS P2 – Conceptual disorganization | 1.8 | 4.1 |
| Symptoms | PANSS P3 – Hallucinatory behavior | 1.8 | 4.1 |
| Symptoms | PANSS P4 – Excitement | 1.8 | 4.1 |
| Symptoms | PANSS P5 – Grandiosity | 1.8 | 4.1 |
| Symptoms | PANSS P6 – Suspiciousness/persecution | 1.8 | 4.1 |
| Symptoms | PANSS P7 – Hostility | 1.8 | 4.1 |
| Global Functioning | GAF Disability/Impairment Lifetime | 0.9 | 0.0 |
| Global Functioning | GAF Disability/Impairment Past Month | 0.0 | 0.0 |
| Global Functioning | GAF Disability Impairment Past Year | 0.0 | 0.0 |
| Global Functioning | GAF Symptoms Lifeimte | 0.9 | 0.0 |
| Global Functioning | GAF Symptoms Past Month | 0.0 | 0.0 |
| Global Functioning | GAF Symptoms Past Year | 0.0 | 0.0 |
| Global Functioning | GF: Role Current | 0.9 | 0.0 |
| Global Functioning | GF: Role Lowest Past Year | 0.9 | 0.0 |
| Global Functioning | GF: Role Highest Past Year | 0.9 | 0.0 |
| Global Functioning | GF: Role Highest Lifetime | 1.8 | 0.0 |
| Global Functioning | GF: Social Current | 0.9 | 0.0 |
| Global Functioning | GF: Social Lowest Past Year | 0.9 | 0.0 |
| Global Functioning | GF: Social Highest Past Year | 0.9 | 0.0 |
| Global Functioning | GF: Social Highest Lifetime | 0.9 | 0.0 |
| CISS 24 | CISS 1 „Try to be with other people.” | 10.1 | 8.2 |
| CISS 24 | CISS 2 „Blame myself for putting things off.” | 11.0 | 5.5 |
| CISS 24 | CISS 3 „Blame myself for having gotten into this situation.” | 10.1 | 6.8 |
| CISS 24 | CISS 4 „Window shop.“ | 10.1 | 6.8 |
| CISS 24 | CISS 5 „Outline my priorities.“ | 10.1 | 6.8 |
| CISS 24 | CISS 6 „Treat myself to a favorite food or snack.” | 11.0 | 6.8 |
| CISS 24 | CISS 7 „Feel anxious about not being able to cope.” | 10.1 | 6.8 |
| CISS 24 | CISS 8 „Become very tense.“ | 10.1 | 6.8 |
| CISS 24 | CISS 9 „Think about how I solved similar problems.“ | 10.1 | 6.8 |
| CISS 24 | CISS 10 „Go out for a snack or meal.” | 10.1 | 6.8 |
| CISS 24 | CISS 11 „Become very upset.“ | 10.1 | 6.8 |
| CISS 24 | CISS 12 „Determine a course of action and follow it.” | 11.0 | 8.2 |
| CISS 24 | CISS 13 „Blame myself for not knowing what to do.” | 10.1 | 6.8 |
| CISS 24 | CISS 14 „Work to understand the situation.” | 10.1 | 6.8 |
| CISS 24 | CISS 15 „Think about the event and learn from my mistakes.” | 11.0 | 6.8 |
| CISS 24 | CISS 16 „Wish that I could change what had happened or how I felt.” | 10.1 | 5.5 |
| CISS 24 | CISS 17 „Visit a friend.“ | 11.0 | 6.8 |
| CISS 24 | CISS 18 „Spend time with a special person.” | 10.1 | 6.8 |
| CISS 24 | CISS 19 „Analyse my problem before reacting.” | 10.1 | 5.5 |
| CISS 24 | CISS 20 „Phone a friend.“ | 10.1 | 5.5 |
| CISS 24 | CISS 21 „Get angry.“ | 10.1 | 6.8 |
| CISS 24 | CISS 22 „See a movie.“ | 10.1 | 6.8 |
| CISS 24 | CISS 23 „Come up with several different solutions to the problem.” | 10.1 | 6.8 |
| CISS 24 | CISS 24 „Try to be organised so I can be on top of the situation.” | 10.1 | 6.8 |
| Sociodemographic data | Age | 0.0 | 0.0 |
| Sociodemographic data | Population Density of place of living | 0.9 | 0.0 |
| Sociodemographic data | Population Density of Place of birth | 4.6 | 5.5 |
| Sociodemographic data | Lived in a partnership for at least one year | 1.8 | 0.0 |
| Sociodemographic data | Number of people living with | 2.8 | 1.4 |
| Sociodemographic data | Years of education | 1.8 | 1.4 |
| Sociodemographic data | Type of current work: Home work (looking after family or home) | 0.0 | 0.0 |
| Sociodemographic data | Type of current work: in full time education | 22.9 | 13.7 |
| Sociodemographic data | Type of current work: unemployed but available for work (with regard to health) | 22.9 | 13.7 |
| Sociodemographic data | Type of current work: unable to work because of physical long-term sickness or disability | 16.5 | 13.7 |
| Sociodemographic data | Type of current work: unable to work because of mental long-term sickness or disability | 22.9 | 13.7 |
| Sociodemographic data | Type of current work: other | 16.5 | 13.7 |
| Sociodemographic data | Sex | 0.0 | 0.0 |
| Body Mass Index | Body Mass Index | 0.9 | 8.3 |
| Cognition (CPT) | Number correct responses overall (True positives) | 0.0 | 4.1 |
| Cognition (CPT) | Number errors distracting stimuli overall (False positives) | 0.0 | 4.1 |
| Cognition (CPT) | Reaction time correct responses overall | 0.0 | 4.1 |
| Cognition (RAVLT) | 1. immediate repetition list A | 10.5 | 9.6 |
| Cognition (RAVLT) | 2. immediate repetition list A | 10.5 | 9.6 |
| Cognition (RAVLT) | 3. immediate repetition list A | 10.5 | 8.2 |
| Cognition (RAVLT) | 4. immediate repetition list A | 10.5 | 8.2 |
| Cognition (RAVLT) | 5. immediate repetition list A | 10.5 | 8.2 |
| Cognition (BDS) | Number of correct trials auditory digit span backward | 0.0 | 0.0 |
| Cognition (DANVA) | Number of correctly recognized faces | 0.0 | 0.0 |
| Cognition (DSST) | Number of correctly matched symbols – Number of incorrectly matched symbols | 1.9 | 4.1 |
| Cognition (FDS) | Number of correctly remembered digit strings | 0.0 | 0.0 |
| Cognition (PVF) | Number of correct words from a phonetic category in 60 seconds | 0.0 | 1.4 |
| Cognition (PVF) | Number of incorrect words from a phonetic category in 60 seconds | 0.0 | 1.4 |
| Cognition (PVF) | Number of repeated words from a phonetic category in 60 seconds | 0.0 | 1.4 |
| Cognition (SVF) | Number of correct words from a semantic category in 60 seconds | 0.0 | 1.4 |
| Cognition (SVF) | Number of incorrect words from a semantic category in 60 seconds | 1.0 | 1.4 |
| Cognition (SVF) | Number of repeated words from a semantic category in 60 seconds | 1.0 | 1.4 |
| Cognition (ROCF) | Accuracy: sum score of all elements – phase 2 (drawing from memory immediately after copying) | 1.9 | 4.1 |
| Cognition (ROCF) | Accuracy: sum score of all elements – phase 3 (drawing from memory 30 minutes after copying) | 1.9 | 4.1 |
| Cognition (ROCF) | Placement: sum score of all elements – phase 1 (drawing from figure) | 1.9 | 4.1 |
| Cognition (ROCF) | Placement: sum score of all elements – phase 2 (drawing from memory immediately after copying) | 1.9 | 4.1 |
| Cognition (ROCF) | Placement: sum score of all elements – phase 3 (drawing from memory 30 minutes after copying) | 1.9 | 4.1 |
| Cognition (ROCF) | Time of execution: phase 1 (drawing from figure) | 2.9 | 4.1 |
| Cognition (ROCF) | Time of execution: phase 2 (drawing from memory immediately after copying) | 5.7 | 4.1 |
| Cognition (ROCF) | Time of execution: phase 3 (drawing from memory 30 minutes after copying) | 5.7 | 4.1 |
| Cognition (GTMA) | Time of execution TMT-A | 3.8 | 8.2 |
| Cognition (GTMB) | Time of execution TMT-B | 3.8 | 8.2 |
| Cognition (SOPT) | Maximum correct responses before error by 4 elements – trial 1 | 0.0 | 1.4 |
| Cognition (SOPT) | Maximum correct responses before error by 4 elements – trial 2 | 0.0 | 1.4 |
| Cognition (SOPT) | Maximum correct responses before error by 4 elements – trial 3 | 0.0 | 1.4 |
| Cognition (SOPT) | Maximum correct responses before error by 6 elements – trial 1 | 1.0 | 4.1 |
| Cognition (SOPT) | Maximum correct responses before error by 6 elements – trial 2 | 1.0 | 4.1 |
| Cognition (SOPT) | Maximum correct responses before error by 6 elements – trial 3 | 1.0 | 4.1 |
| Cognition (SOPT) | Maximum correct responses before error by 8 elements – trial 1 | 0.0 | 4.1 |
| Cognition (SOPT) | Maximum correct responses before error by 8 elements – trial 2 | 0.0 | 4.1 |
| Cognition (SOPT) | Maximum correct responses before error by 8 elements – trial 3 | 0.0 | 4.1 |
| Cognition (SOPT) | Maximum correct responses before error by 10 elements – trial 1 | 0.0 | 4.1 |
| Cognition (SOPT) | Maximum correct responses before error by 10 elements – trial 2 | 0.0 | 4.1 |
| Cognition (SOPT) | Maximum correct responses before error by 10 elements – trial 3 | 1.0 | 4.1 |
| Cognition (WAIS) | Matrices test-raw score | 4.8 | 5.5 |
| Cognition (WAIS) | Matrices test–standard score | 5.7 | 5.5 |
| Cognition (WAIS) | Vocabulary test-raw score | 4.8 | 1.4 |
| Cognition (WAIS) | Vocabulary test-standard score | 6.7 | 1.4 |

*Abbreviations:* DSM-IV = Diagnostic and Statistical Manual of Mental Disorders, fourth edition, ROP = recent-onset psychosis, CHR = clinical high-risk for psychosis, CTQ = Childhood Trauma Questionnaire, WSS = Wisconsin Schizotypy scales, BDI-II = Beck Depression Inventory II, PANSS = Positive and Negative Syndrome Scale, GAF = Global Assessment of Functioning, GF: Social/Role = Global Functioning: Social/Role, CISS 24 = Coping Inventory for Stressful Situations – 24 items, CPT-IP (BACS) = Continuous-Performance Test – Identical Pairs (adapted tablet version), RAVLT = Rey Auditory Verbal Learning test, DANVA = Diagnostic Analysis of Non-Verbal Accuracy 2 (adapted tablet version), DSST = Digit-Symbol-Substitution Test from the BACS battery, DS (F/B) = Auditory Digit Span (Forward/Backward) adapted from the PEBL battery, PVF = phonetic verbal fluency test, SVF = semantic verbal fluency test, ROCF = Rey-Osterrieth complex figure, TMT-A/-B = Trail-Making Test A and B, SOPT = self-ordered pointing task (adapted version), WAIS-III = Wechsler Adult Intelligence Scale (3^rd^ edition)

***In one of the research sites (Turku) the revised version of the Hopkins Verbal Learning Test (HVLT-R) was included instead of the RAVLT, which is not available in Finnish language. Due to this inconsistency, these values were changed to “missing”, and imputed during feature engineering in the cross-validation scheme for all subjects that had performed the HVLT-R.**

## sTable 3 sMRI protocol per Site

| **PRONIA Site** | **Model** | **Field Strength** | **Flip Angle** | **TR (ms)** | **TE (ms)** | **Voxel size [mm]** | **FOV** | **Slice Number** |
| --- | --- | --- | --- | --- | --- | --- | --- | --- |
| Munich | Philips Ingenia | 3T | 8 | Shortest (9.4) | Shortest (5.5) | 0.97 x 0.97 x 1.0 | 250 x 250 | 190 |
| Milan Niguarda | Philips Achieva Intera | 1.5T | 12 | Shortest (8.1) | Shortest (3.7) | 0.94 x 0.94 x 1.0 | 240 x 240 | 170 |
| Basel | SIEMENS Verio / Prisma | 3T | 8 | 2000 | 3.4 | 1.0 x 1.0 x 1.0 | 256 x 256 | 176 |
| Cologne | Philips Achieva | 3T | 8 | 9.5 | 5.5 | 0.97 x 0.97 x 1.0 | 250 x 250 | 190 / 165 |
| Birming-  ham | Philips Achieva | 3T | 8 | 8.4 | 3.8 | 1.0 x 1.0 x 1.0 | 288 x 288 | 175 |
| Turku | Philips Ingenuity | 3T | 7 | 8.1 | 3.7 | 1.0 x 1.0 x 1.0 | 256 x 256 | 176 |
| Muenster | SIEMENS Prisma^fit^ | 3T | 8 | 2130 | 2.3 | 1.0 x 1.0 x 1.0 | 256 x 256 | 192 |

*Abbreviations:* T = tesla, TR = repetition time, TE = echo time, ms = milliseconds, mm = millimeter, FOV = field of view

## sTable 4 Healthy individuals used for harmonization between sites and regression of age- and sex specific effects

| **Institute** | **Sample Size** | **Sex [Female (%)]** | **Statistical Analysis** | ***p-values*** | **Age [mean (SD) years]** | **Statistical Analysis** | ***p-values*** |
| --- | --- | --- | --- | --- | --- | --- | --- |
| **Munich** | 22 | 12 (55.5) | χ^2^_6_ = 0.24 | 1.000 | 26.9 (4.8) | F_6_ = 0.57 | .751 |
| **Milan** | 22 | 12 (55.5) |  |  | 27.0 (4.9) |  |  |
| **Basel** | 22 | 12 (55.5) |  |  | 26.4 (4.3) |  |  |
| **Cologne** | 22 | 12 (55.5) |  |  | 27.0 (5.2) |  |  |
| **Birmingham** | 22 | 11 (50.0) |  |  | 25.5 (6.1) |  |  |
| **Turku** | 22 | 12 (55.5) |  |  | 27.2 (5.2) |  |  |
| **Muenster** | 12 | 7 (58.3) |  |  | 24.7 (4.1) |  |  |

*Abbreviations:* df = degrees of freedom, SD = standard deviation

## sTable 5 Association between component-loadings derived with group information-guided independent component analysis and site before and after ComBat-harmonization in healthy individuals

| **Component** | **Before ComBat** | | | | **After ComBat** | | | |
| --- | --- | --- | --- | --- | --- | --- | --- | --- |
|  | **Sum of Squares** | **df** | **F** | ***p-values*** | **Sum of Squares** | **df** | **F** | ***p-values*** |
| **COI – 1** | 0.000 | 6, 137 | 1.15 | .345 | 0.000 | 6, 137 | 0.29 | .940 |
| **COI – 2** | 0.000 | 6, 137 | 1.39 | .224 | 0.000 | 6, 137 | 0.69 | .657 |
| **COI – 3** | 0.001 | 6, 137 | 8.81 | <.001 | 0.000 | 6, 137 | 0.71 | .644 |
| **COI – 4** | 0.001 | 6, 137 | 7.41 | <.001 | 0.000 | 6, 137 | 0.31 | .932 |
| **COI – 5** | 0.001 | 6, 137 | 1.78 | .108 | 0.000 | 6, 137 | 0.21 | .975 |
| **COI – 6** | 0.002 | 6, 137 | 3.39 | .004 | 0.000 | 6, 137 | 0.47 | .830 |
| **COI – 7** | 0.000 | 6, 137 | 1.07 | .384 | 0.000 | 6, 137 | 0.07 | .999 |
| **COI – 8** | 0.001 | 6, 137 | 8.10 | <.001 | 0.000 | 6, 137 | 1.79 | .107 |
| **COI – 9** | 0.001 | 6, 137 | 2.53 | .024 | 0.000 | 6, 137 | 0.67 | .669 |

*Abbreviations:* df = degrees of freedom, COI = component of interest

## sTable 6 Associations between site and component-loadings derived with group information-guided independent component analysis before and after application of ComBat-harmonization estimates learned in healthy individuals applied to patients with recent-onset psychosis and clinical high-risk for psychosis patients

| **patients with recent-onset psychosis** | | | | | | | | |
| --- | --- | --- | --- | --- | --- | --- | --- | --- |
| **Component** | **Before ComBat** | | | | **After ComBat** | | | |
|  | **Sum of Squares** | **df** | **F** | ***p-values*** | **Sum of Squares** | **df** | **F** | ***p-values*** |
| **COI – 1** | 0.000 | 6, 94 | 0.44 | .848 | 0.000 | 6, 94 | 0.36 | .903 |
| **COI – 2** | **0.001** | **6, 94** | **2.95** | **.011** | **0.001** | **6, 94** | **2.54** | **.025** |
| **COI – 3** | 0.000 | 6, 94 | 1.33 | .254 | **0.000** | **6, 94** | **2.26** | **.044** |
| **COI – 4** | **0.002** | **6, 94** | **9.32** | **<.001** | 0.000 | 6, 94 | 0.96 | .455 |
| **COI – 5** | 0.001 | 6, 94 | 1.45 | .206 | 0.001 | 6, 94 | 0.93 | .481 |
| **COI – 6** | 0.000 | 6, 94 | 0.92 | .482 | 0.000 | 6, 94 | 0.49 | .818 |
| **COI – 7** | 0.000 | 6, 94 | 2.01 | .071 | **0.001** | **6, 94** | **2.22** | **.048** |
| **COI – 8** | **0.000** | **6, 94** | **2.48** | **.028** | **0.001** | **6, 94** | **2.34** | **.038** |
| **COI – 9** | 0.000 | 6, 94 | 1.05 | .401 | **0.001** | **6, 94** | **2.64** | **.021** |
|  | | | | | | | | |
| **patients with clinical high-risk for psychosis** | | | | | | | | |
| **Component** | **Before ComBat** | | | | **After ComBat** | | | |
|  | **Sum of Squares** | **df** | **F** | ***p-values*** | **Sum of Squares** | **df** | **F** | ***p-values*** |
| **COI – 1** | **0.001** | **6, 54** | **3.09** | **.011** | **0.001** | **6, 54** | **2.74** | **.021** |
| **COI – 2** | **0.001** | **6, 54** | **3.01** | **.013** | **0.001** | **6, 54** | **2.70** | **.023** |
| **COI – 3** | 0.000 | 6, 54 | 1.35 | .250 | **0.001** | **6, 54** | **2.76** | **.021** |
| **COI – 4** | **0.001** | **6, 54** | **3.36** | **.007** | 0.000 | 6, 54 | 0.99 | .440 |
| **COI – 5** | 0.001 | 6, 54 | 1.45 | .214 | 0.002 | 6, 54 | 2.11 | .068 |
| **COI – 6** | 0.001 | 6, 54 | 1.20 | .323 | 0.001 | 6, 54 | 1.35 | .253 |
| **COI – 7** | **0.001** | **6, 54** | **3.12** | **.011** | **0.001** | **6, 54** | **2.57** | **.029** |
| **COI – 8** | 0.000 | 6, 54 | 1.68 | .145 | 0.001 | 6, 54 | 1.22 | .313 |
| **COI – 9** | **0.001** | **6, 54** | **2.58** | **.028** | 0.001 | 6, 54 | 1.70 | .138 |

*Abbreviations:* df = degrees of freedom, COI = component of interest

## sTable 7 Matched recent-onset psychosis sample based on age, sex, site and scores of the Global Assessment of Functioning (functional disability) to the original sample

|  | **“Artificial CCu”** | **“Artificial DCu”** | **Statistical Analysis** | ***p-values*** |
| --- | --- | --- | --- | --- |
| **Patients with recent-onset psychosis not included in original sample (N = 109)** | | | | |
| Sample Size [N] | 54 | 55 |  |  |
| Munich (%) | 29 (53.7) | 25 (45.5) | χ^2^_9_ = 11.90 | .156 |
| Milan (%) | 4 (7.4) | 3 (5.5) |  |  |
| Basel (%) | 9 (16.7) | 3 (5.5) |  |  |
| Cologne (%) | 2 (3.7) | 9 (16.4) |  |  |
| Birmingham (%) | 3 (5.6) | 3 (5.5) |  |  |
| Turku (%) | 4 (7.4) | 7 (12.7) |  |  |
| Udine (%) | 0 (0.0) | 0 (0.0) |  |  |
| Bari (%) | 0 (0.0) | 2 (3.6) |  |  |
| Duesseldorf (%) | 1 (1.9) | 0 (0.0) |  |  |
| Muenster (%) | 3 (5.6) | 2 (3.6) |  |  |
| Age [mean (SD) years] | 24.6 (5.5) | 25.7 (6.3) | t_105_ = -0.96 | .338 |
| Sex [Female (%)] | 23 (42.6) | 25 (45.5) | χ^2^_1_ = 0.309 | .578 |
| GF-Social: Highest Lifetime | 7.6 (1.1) | 7.8 (0.8) | t_98_ = -1.15 | .255 |
| **GF-Social: Baseline** | **5.5 (1.6)** | **6.1 (1.1)** | **t_95_ = -2.09** | **.040** |
| GF-Role: Highest Lifetime | 7.7 (1.2) | 7.9 (1.0) | t_103_ = -0.60 | .551 |
| GF-Role: Baseline | 4.9 (2.0) | 5.4 (1.7) | t_104_ = -1.55 | .125 |
| GAF Disability/Impairment Highest Lifetime | 78.0 (8.1) | 77.7 (13.3) | t_87_ = 0.14 | .889 |
| GAF Disability/Impairment Highest Past Month | 41.8 (12.6) | 45.3 (13.7) | t_106_ = -1.41 | .163 |
| GAF Symptoms Highest Lifetime | 78.7 (9.9) | 77.9 (14.3) | t_94_ = 0.33 | .741 |
| GAF Symptoms Highest Past Month | 40.4 (14.7) | 39.8 (15.1) | t_107_ = 0.21 | .833 |
| Positive and Negative Syndrome Scale – Positive [mean (SD)] | 18.9 (5.6) | 17.8 (6.6) | t_97_ = 0.85 | .397 |
| Positive and Negative Syndrome Scale – Negative [mean (SD)] | 16.8 (8.8) | 14.5 (5.7) | t_92_ = 1.64 | .104 |
| Positive and Negative Syndrome Scale – General [mean (SD)] | 35.9 (10.0) | 33.5 (10.2) | t_101_ = 1.21 | .228 |
| No Cannabis User Baseline (%) | 19 (35.2) | 17 (30.9) | t_90_ = 1.22 | .227 |

*Abbreviations:* df = degrees of freedom, GAF = Global Assessment of Functioning, GF: Social/Role = Global Functioning: Social/Role, CCu = continued cannabis use, DCu = discontinued cannabis use

## sTable 8 Differences between patients with continued and discontinued cannabis use

|  | **CCu** | **DCu** | **Statistical Analysis** | ***p-values*** | **CCu** | **DCu** | **Statistical Analysis** | ***p-values*** |
| --- | --- | --- | --- | --- | --- | --- | --- | --- |
| **Discovery Sample (ROP; N=109)** | | | | | **Validation Sample (CHR; N=73)** | | | |
| Sample Size [N (%)] | 54 (49.5) | 55 (50.5) |  |  | 36 (49.3) | 37 (50.7) |  |  |
| Number of Other Substances besides Cannabis tried in Lifetime [mean (SD)] | **1.7 (1.7)** | **0.6 (1.1)** | **t_62_ = 3.6** | **<.001** | 0.6 (1.2) | 0.7 (0.9) | t_43_ = -0.31 | .756 |
| Lifetime History of DSM-IV Cannabis Use Disorder [N (%)] | | | | | | | | |
| Cannabis Abuse (%) | **21 (38.9)** | **19 (34.5)** | **χ^2^_2_ = -9.61** | **.008** | 16 (44.4) | 12 (32.4) | χ^2^_2_ = -1.14 | .566 |
| Cannabis Dependency (%) | **17 (31.5)** | **6 (10.9)** |  |  | 1 (2.8) | 1 (2.7) |  |  |
| Average Number of cigarettes per day [mean (SD)] | 8.0 (7.4) | 7.2 (7.6) | t_100_ = 0.60 | .553 | 7.7 (8.6) | 4.6 (6.1) | t_63_ = 1.71 | .093 |
| Average Units of alcohol consumed per day [mean (SD)] | 4.4 (4.2) | 5.4 (6.1) | t_75_ = -0.92 | .360 | 4.2 (5.6) | 4.0 (3.3) | t_46_ = 0.24 | .811 |
| Level of Cannabis Use in the Last 3 Months – Cumulative Frequency (%) | | | | | | | | |
| 0 times | 11 (20) | 22 (40) | χ^2^_6_ = 5.52 | .479 | **9 (25)** | **23 (62)** | **χ^2^_4_ = 11.22** | **.024** |
| 1-5 times | 7 (13) | 7 (13) |  |  | **8 (22)** | **6 (16)** |  |  |
| 6-10 times | 4 (7) | 3 (5) |  |  | **2 (6)** | **0 (0)** |  |  |
| 11-15 times | 4 (7) | 1 (2) |  |  | **2 (6)** | **1 (3)** |  |  |
| 16-20 times | 1 (2) | 1 (2) |  |  | **0 (0)** | **0 (0)** |  |  |
| 21-30 times | 2 (4) | 1 (2) |  |  | **0 (0)** | **0 (0)** |  |  |
| > 30 times | 8 (15) | 8 (15) |  |  | **3 (8)** | **0 (0)** |  |  |
| Level of Use of Other Substances in the Last 3 Months – Cumulative Frequency (%) | | | | | | | | |
| 0 times | 17 (31) | 29 (53) | χ^2^_5_ = 5.55 | .353 | 15 (42) | 11 (30) | χ^2^_2_ = 2.90 | .234 |
| 1-5 times | 7 (13) | 3 (5) |  |  | 2 (6) | 5 (14) |  |  |
| 6-10 times | 2 (4) | 1 (2) |  |  | 0 (0) | 1 (3) |  |  |
| 11-15 times | 1 (2) | 1 (2) |  |  | 0 (0) | 0 (0) |  |  |
| 16-20 times | 0 (0) | 0 (0) |  |  | 0 (0) | 0 (0) |  |  |
| 21-30 times | 1 (2) | 0 (0) |  |  | 0 (0) | 0 (0) |  |  |
| > 30 times | 1 (2) | 1 (2) |  |  | 0 (0) | 0 (0) |  |  |
| Age at Cannabis Initiation [mean (SD) years] | 16.5 (2.6) | 17.5 (3.7) | t_86_ = -1.55 | .126 | 17.1 (2.2) | 17.5 (2.7) | t_56_ = -0.64 | .526 |
| Duration Lifetime Cannabis Use [mean (SD) months] | 63.7 (50.0) | 51.8 (43.9) | t_90_ = 1.22 | .227 | 52.9 (64.4) | 33.7 (43.4) | t_45_ = 1.31 | .195 |
| Time since last Cannabis Use at Baseline [mean (SD) months] | **8.7 (25.3)** | **33.4 (60.9)** | **t_66_ = -2.52** | **.010** | **10.6 (29.2)** | **33.6 (53.8)** | **t_50_ = -2.14** | **.038** |
| Sum of experienced burden of Recent Life Events (last year) [mean (SD)] | 9.3 (10.9) | 12.3 (11.4) | t_104_ = -1.40 | .164 | 15.1 (10.4) | 13.8 (9.9) | t_71_ = 0.53 | .595 |
| CTQ (emotional abuse) [mean (SD)] | 12.3 (3.3) | 11.4 (3.1) | t_91_ = 1.31 | .193 | **13.3 (3.2)** | **11.5 (2.3)** | **t_61_ = 2.67** | **.010** |
| CTQ (emotional neglect) [mean (SD)] | 10.9 (4.2) | 10.6 (4.8) | t_86_ = 0.35 | .727 | **12.7 (4.4)** | **10.6 (4.0)** | **t_66_ = 2.08** | **.041** |
| CTQ (physical abuse) [mean (SD)] | 7.0 (3.8) | 6.4 (3.4) | t_93_ = 0.79 | .433 | **7.7 (3.6)** | **5.8 (2.1)** | **t_56_ = 2.66** | **.010** |
| CTQ (physical neglect) [mean (SD)] | 7.8 (3.1) | 6.8 (2.0) | t_83_ = 1.87 | .064 | 7.9 (2.6) | 6.8 (2.4) | t_64_ = 1.88 | .064 |
| CTQ (sexual abuse) [mean (SD)] | 5.7 (2.0) | 6.0 (2.0) | t_89_ = -0.73 | .468 | 5.8 (1.9) | 6.6 (3.4) | t_51_ = -1.21 | .232 |
| WSS (magical ideation) [mean (SD)] | **4.9 (3.3)** | **3.3 (3.7)** | **t_89_ = 2.60** | **.011** | 3.3 (2.9) | 2.9 (2.7) | t_62_ = 0.58 | .564 |
| WSS (perceptual aberration) [mean (SD)] | **3.5 (4.3)** | **1.7 (2.8)** | **t_87_ = 2.53** | **.013** | 2.1 (2.3) | 3.1 (3.2) | t_60_ = -1.38 | .172 |
| WSS (physical anhedonia) [mean (SD)] | 4.3 (3.2) | 3.3 (2.7) | t_88_ = 1.62 | .109 | 3.8 (2.9) | 2.6 (2.4) | t_64_ = 1.83 | .072 |
| WSS (social anhedonia) [mean (SD)] | **4.8 (3.0)** | **3.4 (3.1)** | **t_89_ = 2.19** | **.031** | 6.0 (3.5) | 4.6 (2.9) | t_62_ = 1.79 | .079 |
| Becks Depression Inventary – II [mean (SD)] | | | | | | | | |
| BDI-II 1 - Sadness | 0.9 (0.9) | 0.7 (0.6) | t_90_ = 1.27 | .209 | 1.4 (0.8) | 1.3 (0.8) | t_67_ = 0.39 | .696 |
| BDI-II 2 - Pessimism | 0.9 (0.9) | 0.7 (0.8) | t_97_ = 1.10 | .273 | 1.3 (0.9) | 1.1 (0.9 | t_68_ = 0.66 | .513 |
| BDI-II 3 – Past failures | 1.2 (1.0) | 0.9 (0.8) | t_96_ = 1.49 | .140 | 1.8 (1.0) | 1.4 (1.0) | t_68_ = 1.57 | .121 |
| BDI-II 4 – Loss of Pleasure | 1.0 (0.9) | 0.9 (0.8) | t_96_ = 0.72 | .473 | 1.4 (8.5) | 1.5 (0.8) | t_68_ = -0.59 | .559 |
| BDI-II 5 – Guilt Feelings | 0.9 (0.8) | 0.7 (0.8) | t_97_ = 1.08 | .284 | 1.2 (1.0) | 1.0 (0.8) | t_65_ = 0.82 | .418 |
| BDI-II 6 – Punishment Feelings | 1.0 (1.0) | 0.8 (1.0) | t_97_ = 1.31 | .192 | 1.0 (1.1) | 0.8 (1.0) | t_68_ = 0.68 | .497 |
| BDI-II 7 – Self Dislike | 1.0 (1.0) | 0.9 (0.9) | t_96_ = 0.55 | .582 | 1.3 (0.9) | 1.4 (1.0) | t_67_ = -0.13 | .901 |
| BDI-II 8 – Self Criticalness | 1.2 (1.1) | 0.9 (0.8) | t_94_ = 1.63 | .106 | 1.3 (1.1) | 1.3 (0.9) | t_65_ = 0.00 | 1.000 |
| BDI-II 9 – Suicidal thoughts or wishes | 0.5 (0.7) | 0.5 (0.6) | t_96_ = 0.70 | .485 | 1.1 (0.7) | 0.8 (0.6) | t_67_ = 1.79 | .078 |
| BDI-II 10 – Crying | 0.7 (1.1) | 0.6 (0.9) | t_96_ = 0.58 | .561 | 1.2 (1.2) | 1.2 (1.2) | t_67_ = -0.18 | .858 |
| BDI-II 11 - Agitation | 1.0 (0.9) | 0.8 (0.7) | t_95_ = 1.35 | .179 | 1.1 (0.9) | 1.1 (0.7) | t_64_ = 0.28 | .779 |
| BDI-II 12 – Loss of Interest | 1.1 (1.0) | 1.0 (1.1) | t_96_ = 0.66 | .513 | 1.2 (1.0) | 1.3 (1.0) | t_67_ = -0.22 | .830 |
| BDI-II 13 – Indecisiveness | 1.2 (1.1) | 1.0 (1.0) | t_97_ = 0.83 | .406 | 1.6 (1.2) | 1.7 (1.0) | t_67_ = -0.22 | .827 |
| BDI-II 14 – Worthlessness | 0.9 (1.0) | 0.8 (0.8) | t_96_ = 0.73 | .469 | 1.1 (0.8) | 1.3 (1.0) | t_67_ = -0.53 | .597 |
| BDI-II 15 – Loss of Energy | 1.0 (0.8) | 0.9 (0.8) | t_97_ = 0.39 | .695 | 1.2 (0.8) | 1.4 (0.7) | t_67_ = -1.28 | .206 |
| BDI-II 16 – Changes in Sleeping Pattern | 2.3 (1.9) | 1.8 (1.8) | t_97_ = 1.13 | .260 | 2.5 (1.8) | 2.4 (1.9) | t_67_ = 0.22 | .824 |
| BDI-II 17 – Irritability | 0.9 (1.0) | 0.6 (0.7) | t_91_ = 1.96 | .053 | 1.2 (1.1) | 0.9 (0.8) | t_62_ = 1.36 | .177 |
| BDI-II 18 – Changes in Appetite | 1.8 (1.7) | 1.2 (1.5) | t_97_ = 1.87 | .064 | 1.3 (1.6) | 1.6 (1.7) | t_67_ = -0.92 | .360 |
| BDI-II 19 – Concentration Difficulty | 1.4 (1.0) | 1.2 (0.8) | t_96_ = 0.92 | .362 | 1.6 (0.8) | 1.8 (0.7) | t_66_ = -1.07 | .287 |
| BDI-II 20 – Tiredness or Fatigue | 1.1 (0.8) | 1.0 (0.8) | t_97_ = 0.37 | .713 | 1.4 (0.9) | 1.6 (0.7) | t_65_ = -0.85 | .397 |
| BDI-II 21 – Loss of Interest in Sex | 0.8 (1.0) | 0.9 (1.0) | t_95_ = -0.76 | .451 | 0.8 (0.9) | 0.8 (1.0) | t_68_ = 0.13 | .901 |
| Positive and Negative Syndrome Scale [mean (SD)] | | | | | | | | |
| PANSS general 1 - Somatic concern | 2.2 (1.7) | 1.8 (1.6) | t_104_ = 1.33 | .186 | 1.7 (1.0) | 1.8 (1.3) | t_65_ = -0.52 | .606 |
| PANSS general 2 - Anxiety | 2.9 (1.6) | 2.8 (1.6) | t_105_ = 0.53 | .596 | 2.7 (1.1) | 2.7 (1.4) | t_63_ = 0.00 | 1.000 |
| PANSS general 3 - Guilt feelings | 2.1 (1.6) | 2.3 (1.6) | t_105_ = -0.65 | .516 | 2.1 (1.5) | 1.7 (1.2) | t_65_ = 1.42 | .161 |
| PANSS general 4 - Tension | 2.6 (1.4) | 2.2 (1.3) | t_104_ = 1.22 | .225 | 1.9 (0.8) | 1.9 (1.1) | t_63_ = -0.12 | .903 |
| PANSS general 5 - Mannerisms and posturing | **1.5 (1.0)** | **1.2 (0.5)** | **t_78_ = 2.41** | **.018** | 1.2 (0.4) | 1.1 (0.4) | t_68_ = 0.95 | .346 |
| PANSS general 6 – Depression | 3.4 (1.6) | 2.8 (1.6) | t_105_ = 1.85 | .067 | 3.9 (1.3) | 3.8 (1.3) | t_68_ = 0.38 | .707 |
| PANSS general 7 - Motor retardation | 1.6 (1.1) | 1.5 (1.0) | t_103_ = 0.41 | .684 | 1.4 (0.9) | 1.1 (0.4) | t_49_ = 1.98 | .054 |
| PANSS general 8 - Uncooperativeness | 1.5 (1.3) | 1.3 (0.6) | t_75_ = 1.30 | .199 | 1.1 (0.5) | 1.1 (0.4) | t_61_ = 0.26 | .795 |
| PANSS general 9 - Unusual thought content | **3.5 (2.0)** | **2.6 (1.7)** | **t_102_ = 2.46** | **.015** | 1.8 (1.1) | 1.7 (1.1) | t_68_ = 0.34 | .738 |
| PANSS general 10 – Disorientation | 1.5 (early0.8) | 1.3 (0.9) | t_105_ = 0.95 | .344 | 1.2 (0.9) | 1.3 (0.8) | t_67_ = -0.14 | .886 |
| PANSS general 11 - Poor attention | 2.5 (1.3) | 2.2 (1.2) | t_105_ = 1.33 | .188 | 2.0 (1.1) | 2.4 (1.1) | t_68_ = -1.52 | .133 |
| PANSS general 12 - Lack of judgement and insight | 2.9 (1.5) | 2.4 (1.5) | t_105_ = 1.65 | .103 | 1.4 (0.9) | 1.3 (0.6) | t_61_ = 0.63 | .533 |
| PANSS general 13 - Disturbance of volition | 2.1 (1.2) | 2.0 (1.5) | t_103_ = 0.72 | .474 | 2.4 (1.6) | 1.8 (1.2) | t_63_ = 1.72 | .089 |
| PANSS general 14 - Poor impulse control | 1.7 (1.0) | 1.6 (1.3) | t_101_ = 0.39 | .695 | 1.4 (0.7) | 1.4 (0.8) | t_67_ = 0.31 | .758 |
| PANSS general 15 – Preoccupation | **2.2 (1.4)** | **1.7 (1.1)** | **t_100_ = 2.12** | **.036** | 1.8 (1.1) | 1.7 (1.0) | t_68_ = 0.23 | .821 |
| PANSS general 16 - Active social avoidance | **2.8 (1.6)** | **2.1 (1.3)** | **t_99_ = 2.25** | **.027** | 2.6 (1.6) | 2.6 (1.6) | t_68_ = 0.00 | 1.000 |
| PANSS N1 – Blunted affect | 2.3 (1.5) | 2.2 (1.4) | t_104_ = 0.22 | .825 | 2.1 (1.2) | 2.3 (1.4) | t_67_ = -0.36 | .717 |
| PANSS N2 - Emotional withdrawal | 2.6 (1.2) | 2.4 (1.4) | t_104_ = 1.07 | .285 | 2.4 (1.4) | 2.6 (1.6) | t_67_ = -0.57 | .574 |
| PANSS N3 – Poor Rapport | 2.0 (1.3) | 1.9 (1.2) | t_104_ = 0.23 | .822 | 1.9 (1.2) | 2.1 (1.3) | t_68_ = -0.57 | .570 |
| PANSS N4 – Passive/apathetic social withdrawal | 2.6 (1.8) | 2.5 (1.4) | t_102_ = 0.44 | .657 | 2.7 (1.5) | 2.6 (1.4) | t_67_ = 0.33 | .745 |
| PANSS N5 – Difficulty in abstract thinking | 1.8 (1.6) | 1.5 (0.8) | t_100_ = 1.53 | .130 | 1.3 (0.8) | 1.5 (1.0) | t_65_ = -0.51 | .614 |
| PANSS N6 – Lack of spontaneity and flow of conversation | 1.9 (1.0) | 1.8 (1.2) | t_101_ = 0.24 | .808 | 1.8 (1.2) | 1.6 (0.9) | t_64_ = 0.76 | .449 |
| PANSS N7 – Stereotyped thinking | 2.0 (1.3) | 1.8 (1.3) | t_105_ = 0.67 | .507 | 1.5 (1.1) | 1.3 (0.6) | t_51_ = 0.81 | .424 |
| PANSS P1 – Delusions | 4.5 (2.0) | 4.5 (1.9) | t_104_ = -0.17 | .863 | 1.9 (1.8) | 2.1 (1.2) | t_68_ = -0.91 | .368 |
| PANSS P2 - Conceptual disorganization | 2.3 (1.5) | 2.1 (1.4) | t_104_ = 1.03 | .307 | 1.6 (0.8) | 1.7 (1.0) | t_65_ = -0.39 | .699 |
| PANSS P3 – Hallucinatory behavior | 2.8 (1.9) | 3.0 (1.9) | t_105_ = -0.57 | .573 | 1.8 (1.0) | 2.1 (1.3) | t_64_ = -1.33 | .187 |
| PANSS P4 – Excitement | 2.0 (1.3) | 2.0 (1.4) | t_105_ = 0.00 | 1.00 | 1.1 (0.4) | 1.3 (0.6) | t_59_ = -1.51 | .138 |
| PANSS P5 – Grandiosity | 2.5 (1.9) | 2.0 (1.8) | t_104_ = 1.30 | .196 | 1.3 (0.8) | 1.2 (0.7) | t_67_ = 0.15 | .878 |
| PANSS P6 – Suspiciousness/persecution | 4.1 (2.1) | 4.0 (2.2) | t_105_ = 0.36 | .718 | 2.3 (1.2) | 1.8 (.10) | t_66_ = 1.80 | .077 |
| PANSS P7 - Hostility | 1.8 (1.3) | 1.7 (1.2) | t_103_ = 0.67 | .503 | 1.3 (0.7) | 1.2 (0.7) | t_68_ = 0.53 | .601 |
| Global Functioning [mean (SD)] | | | | | | | | |
| GAF Disability/Impairment Highest Lifetime | 77.5 (8.8) | 78.5 (9.0) | t_104_ = -0.58 | .561 | 76.6 (8.6) | 79.0 (8.3) | t_71_ = -1.22 | .227 |
| GAF Disability/Impairment Highest Past Year | **59.4 (14.1)** | **66.5 (14.6)** | **t_107_ = -2.61** | **.010** | 62.5 (12.3) | 66.1 (13.4) | t_71_ = -1.21 | .234 |
| GAF Disability/Impairment Highest Past Month | **41.3 (10.4)** | **49.0 (16.6)** | **t_91_ = -2.92** | **.004** | 48.3 (11.3) | 53.0 (11.2) | t_70_ = -0.52 | .607 |
| GAF Symptoms Highest Lifetime | 77.7 (8.5) | 79.6 (9.6) | t_106_ = -1.07 | .286 | 78.0 (9.9) | 79.1 (8.8) | t_71_ = -1.78 | .079 |
| GAF Symptoms Highest Past Year | **56.6 (15.8)** | **64.7 (16.5)** | **t_107_ = -2.65** | **.009** | 61.9 (11.8) | 65.3 (11.3) | t_71_ = -1.24 | .220 |
| GAF Symptoms Highest Past Month | 40 (12.4) | 43.4 (16.4) | t_100_ = -1.21 | .230 | 47.0 (10.2) | 51.8 (11.8) | t_70_ = -1.86 | .067 |
| GF-Social: Highest Lifetime | 7.8 (0.8) | 8.0 (0.8) | t_106_ = -1.36 | .177 | 7.7 (0.9) | 8.1 (0.8) | t_68_ =-1.64 | .107 |
| GF-Social: Lowest Past Year | 5.2 (1.6) | 5.3 (1.6) | t_106_ = -0.33 | .740 | 5.7 (1.2) | 6.0 (1.5) | t_70_ =-1.05 | .299 |
| GF-Social: Highest Past Year | **6.5 (1.2)** | **7.2 (1.3)** | **t_106_ = -2.7** | **.008** | **6.8 (1.2)** | **7.4 (0.9)** | **t_69_ =-2.37** | **.020** |
| GF-Social: Baseline | 5.6 (1.5) | 5.9 (1.5) | t_106_ = -0.94 | .352 | 5.9 (1.4) | 6.6 (1.5) | t_71_ = -1.93 | .058 |
| GF-Role: Highest Lifetime | **7.4 (0.9)** | **7.9 (0.9)** | **t_105_ = -2.67** | **.009** | 7.9 (0.9) | 7.9 (0.8) | t_69_ = -0.16 | .877 |
| GF-Role: Lowest Past Year | 4.3 (1.7) | 4.9 (1.9) | t_105_ = -1.7 | .094 | 5.2 (1.9) | 5.6 (1.3) | t_63_ = -1.19 | .239 |
| GF-Role: Highest Past Year | **6.0 (1.8)** | **7.2 (1.2)** | **t_93_ = -3.9** | **<.001** | **6.9 (1.2)** | **7.4 (0.9)** | **t_66_ = -2.06** | **.043** |
| GF-Role: Baseline | 4.6 (1.7) | 5.3 (1.9) | t_106_ = -1.96 | .052 | 5.4 (1.9) | 6.0 (1.4) | t_64_ = -1.60 | .114 |
| CISS 1 „Try to be with other people.” | 3.0 (1.2) | 3.1 (1.2) | t_96_ = -0.62 | .540 | 2.6 (1.0) | 2.9 (1.0) | t_64_ = -1.13 | .264 |
| CISS 2 „Blame myself for putting things off.” | 3.8 (1.1) | 3.4 (1.2) | t_92_ = 1.61 | .112 | 3.7 (1.1) | 4.0 (0.) | t_65_ = -0.93 | .356 |
| CISS 3 „Blame myself for having gotten into this situation.” | 3.6 (1.3) | 3.2 (1.2) | t_96_ = 1.30 | .197 | 3.8 (1.2) | 3.7 (1.0) | t_65_ = 0.22 | .823 |
| CISS 4 „Window shop.“ | 2.1 (1.3) | 2.4 (1.2) | t_96_ = -1.22 | .226 | 2.1 (1.3) | 2.4 (1.1) | t_65_ = -1.25 | .218 |
| CISS 5 „Outline my priorities.“ | 3.2 (1.2) | 3.3 (0.9) | t_92_ = -0.87 | .387 | 2.9 (1.0) | 2.9 (1.1) | t_66_ = -0.23 | .818 |
| CISS 6 „Treat myself to a favorite food or snack.” | 3.3 (1.2) | 3.7 (1.2) | t_94_ = -1.59 | .115 | 3.3 (1.2) | 3.4 (1.2) | t_66_ = -0.40 | .692 |
| CISS 7 „Feel anxious about not being able to cope.” | 3.4 (1.2) | 3.3 (1.2) | t_96_ = 0.31 | .758 | 3.9 (1.0) | 3.9 (1.1) | t_66_ = 0.11 | .910 |
| CISS 8 „Become very tense.“ | 3.6 (1.2) | 3.1 (1.3) | t_94_ = 1.71 | .091 | 4.3 (0.8) | 4.1 (1.0) | t_63_ = 0.91 | .368 |
| CISS 9 „Think about how I solved similar problems.“ | 3.1 (1.1) | 3.3 (1.2) | t_94_ = -0.94 | .347 | 3.0 (1.2) | 2.9 (1.1) | t_66_ = 0.21 | .832 |
| CISS 10 „Go out for a snack or meal.” | 2.7 (1.4) | 2.9 (1.3) | t_96_ = -0.55 | .587 | 2.9 (1.2) | 2.7 (1.0) | t_64_ = 0.64 | .523 |
| CISS 11 „Become very upset.“ | 2.9 (1.3) | 2.6 (1.1) | t_95_ = 1.06 | .291 | 3.3 (1.1) | 3.2 (1.2) | t_66_ = 0.31 | .758 |
| CISS 12 „Determine a course of action and follow it.” | 2.9 (1.3) | 3.2 (1.2) | t_95_ = -1.23 | .223 | 2.5 (0.9) | 2.5 (0.8) | t_65_ = -0.35 | .729 |
| CISS 13 „Blame myself for not knowing what to do.” | **3.5 (1.2)** | **2.9 (1.4)** | **t_92_ = 2.28** | **.025** | 3.8 (1.0) | 3.6 (1.0) | t_66_ = 0.61 | .544 |
| CISS 14 „Work to understand the situation.” | 3.9 (1.1) | 4.0 (0.8) | t_92_ = -0.48 | .631 | 4.1 (1.0) | 3.8 (.8) | t_60_ = 1.06 | .291 |
| CISS 15 „Think about the event and learn from my mistakes.” | 3.6 (1.2) | 3.8 (1.1) | t_95_ = -0.85 | .396 | 3.6 (1.2) | 36 (1.1) | t_65_ = -0.11 | .916 |
| CISS 16 „Wish that I could change what had happened or how I felt.” | 3.7 (1.4) | 3.5 (1.3) | t_96_ = 0.79 | .431 | 4.1 (1.1) | 4.0 (1.1) | t_67_ = 0.43 | .672 |
| CISS 17 „Visit a friend.“ | 2.9 (1.2) | 3.2 (1.2) | t_94_ = -0.97 | .334 | 3.0 (1.3) | 2.9 (1.1) | t_65_ = 0.20 | .844 |
| CISS 18 „Spend time with a special person.” | 3.2 (1.1) | 3.4 (1.1) | t_95_ = -0.84 | .403 | 3.3 (1.3) | 3.2 (1.3) | t_66_ = 0.37 | .710 |
| CISS 19 „Analyse my problem before reacting.” | 3.2 (1.2) | 3.3 (1.2) | t_95_ = -0.43 | .667 | 3.7 (1.0) | 3.5 (1.0) | t_67_ = 0.77 | .446 |
| CISS 20 „Phone a friend.“ | 2.6 (1.2) | 3.0 (1.2) | t_-95_ = -1.78 | .079 | 2.5 (1.3) | 2.5 (1.3) | t_67_ = 0.05 | .964 |
| CISS 21 „Get angry.“ | **3.3 (1.2)** | **2.6 (1.4)** | **t_91_ = 2.34** | **.021** | 3.4 (1.2) | 2.9 (1.3) | t_66_ = 1.75 | .085 |
| CISS 22 „See a movie.“ | 3.0 (1.4) | 2.8 (1.4) | t_95_ = 0.77 | .445 | 3.3 (1.2) | 3.2 (1.4) | t_65_ = 0.28 | .782 |
| CISS 23 „Come up with several different solutions to the problem.” | 3.1 (1.2) | 3.1 (1.1) | t_96_ = -0.30 | .762 | 3.1 (1.1) | 2.7 (1.0) | t_65_ = 1.48 | .145 |
| CISS 24 „Try to be organised so I can be on top of the situation.” | 3.1 (1.2) | 3.6 (1.2) | t_96_ = -2.11 | .037 | 2.6 (1.2) | 2.9 (1.2) | t_66_ = -0.94 | .349 |
| Age [mean (SD) years] | 23.8 (4.3) | 25.1 (5.2) | t_104_ = -1.45 | .151 | 21.96 (3.65) | 23.8 (5.2) | t_65_ = -1.80 | .076 |
| Population density of place of living [mean (SD)] | 3146 (2479) | 2851 (2208) | t_104_ = 0.65 | .517 | 2830 (2476) | 2625 (1970) | t_67_ = 0.39 | .698 |
| Population density of place of birth [mean (SD)] | 3889 (6315) | 2976 (4534) | t_88_ = 0.84 | .402 | 3056 (3997) | 2032 (1905) | t_43_ = 1.33 | .192 |
| Lived in a partnership for at least one year [yes (%)] | 24 (44) | 29 (53) | χ^2^_1_ = 0.24 | .627 | **11 (31)** | **23 (62)** | **χ^2^_1_ = 6.11** | **.013** |
| Number of people living with [mean (SD)] | 6.5 (27.8) | 2.5 (2.6) | t_51_ = 1.01 | .318 | 3.4 (3.0) | 2.5 (1.7) | t_56_ = 1.51 | .138 |
| Education [mean (SD) years] | 13.3 (2.7) | 13.9 (2.7) | t_105_ = -1.12 | .268 | 13.1 (2.6) | 14.2 (2.7) | t_70_ = -1.64 | .106 |
| Type of current work [N (%)] | | | | | | | | |
| Home work (looking after family or home) | 0 (0) | 0 (0) | χ^2^_4_ = 7.16 | .128 | **0 (0)** | **0 (0)** | **χ^2^_3_ = 8.16** | **.043** |
| in full time education | 14 (26) | 21 (38) |  |  | **16 (44)** | **22 (59)** |  |  |
| unemployed but available for work (with regard to health) | 9 (17) | 7 (13) |  |  | **1 (3)** | **5 (14)** |  |  |
| unable to work because of physical long-term sickness or disability | 0 (0) | 1 (2) |  |  | **0 (0)** | **0 (0)** |  |  |
| unable to work because of mental long-term sickness or disability | 21 (39) | 10 (18) |  |  | **13 (36)** | **5 (16)** |  |  |
| other | 1 (2) | 0 (0) |  |  | **1 (3)** | **0 (0)** |  |  |
| Sex [Female (%)] | 15 (27.8) | 19 (34.5) | χ^2^_1_ = 0.31 | .578 | **11 (30.6)** | **23 (62.2)** | **χ^2^_1_ = -6.11** | **.013** |
| Body Mass Index [mean (SD)] | 23.1 (4.1) | 22.9 (4.0) | t_97_ = -0.30 | .763 | 23.5 (4.4) | 22.0 (2.9) | t_59_ = 1.71 | .092 |

*bold: significant at p<.05

*Abbreviations:* CCu = continued cannabis use, DCu = discontinued cannabis use, ROP = recent-onset psychosis, CHR = clinical high-risk for psychosis, GAF = Global Assessment of functioning, GF = Global Functioning, WSS = Wisconsin Schizotypyp Scale, PANSS G= Positive and Negative Syndrome Scale general, PANSS P= Positive and Negative Syndrome Scale positive, PANSS N= Positive and Negative Syndrome Scale negative, CISS = Coping Inventory of Stressful Situations, BDI-II = Beck’s Depression Inventory – II, SD = standard deviation

## sTable 9 Prediction results in recent-onset psychosis matched based on age, sex, site and scores from the Global Assessment of Functioning (functional disability) to original sample

|  | **TP** | **TN** | **FP** | **FN** | **Sens%** | **Spec%** | **BAC%** | **PPV** | **NPV** | **PSI** | **NLR** | **PLR** | **AUC** | **p-value** |
| --- | --- | --- | --- | --- | --- | --- | --- | --- | --- | --- | --- | --- | --- | --- |
| **Clinical predictor** | | | | | | | | | | | | | | |
| ROP (N = 109) | 30 | 32 | 22 | 25 | 54.5 | 59.3 | 56.9 | 57.5 | 56.1 | 13.8 | 0.8 | 1.3 | 0.62 | .083 |
| **sMRI predictor** | | | | | | | | | | | | | | |
| ROP (N = 101) | 18 | 25 | 26 | 32 | 36.0 | 49.0 | 42.5 | 40.9 | 43.9 | -15.2 | 1.3 | 0.7 | 0.43 | .989 |

*Abbreviations:* TP = true positive, TN = true negative, FP = false positive, FN = false negative, Sens % = Sensitivity, BAC = balanced accuracy, PPV = positive predictive value, NPV = negative predictive value, PSI = prognostic summary index, NLR = negative likelihood ratio, PLR = positive likelihood ratio, AUC = area under the curve, ROP = recent-onset psychosis

## sTable 10 Results of Mixed-model analysis of illness course

The degrees of freedom were approximated using Satterthwaite method (lmerTest). Parametric permutation-based Likelihood Ratio Tests (LRT) were conducted with 200 permutations to determine significant benefits of adding additional predictors (Time^2^, Outcome:Time^2^; Time^3^, Outcome:Time^3^) in comparison with the linear model. We present here the comparison of the model providing the best fit based on LRT (quadratic/polynomial) with the linear fit. In case that the linear model provided the best fit we present the LRT-test result in comparison with the quadratic model.

|  | **Recent-Onset Psychosis** | | | | | **Clinical High-Risk for Psychosis** | | | | |
| --- | --- | --- | --- | --- | --- | --- | --- | --- | --- | --- |
| **Positive and Negative Syndrome Scale – Positive** | | | | | | | | | | |
| **Term** | **df** | **t-value** | **p_FDR_** | **LRT χ^2^_(df)_** | **LRT p_(FDR)_** | **df** | **t-value** | **p_FDR_** | **LRT χ^2^_(df)_** | **LRT p_(FDR)_** |
| Group | 205 | 2.22 | .042 | 53.66_4_ | <.001 | 114 | 0.48 | .759 | 3.37_2_ | .186 |
| Time (linear) | 306 | -5.73 | <.001 |  |  | 261 | -3.72 | <.001 |  |  |
| Time (quadratic) | 312 | 2.76 | .010 |  |  | - | - | - |  |  |
| Time (polynomial) | 316 | -1.64 | .110 |  |  | - | - | - |  |  |
| Time x Group | 315 | -1.38 | .420 |  |  | 261 | 0.38 | .779 |  |  |
| Time^2^ x Group | 318 | 1.55 | .420 |  |  | - | - | - |  |  |
| Time^3^ x Group | 320 | -1.34 | .420 |  |  | - | - | - |  |  |
| **Positive and Negative Syndrome Scale – General** | | | | | | | | | | |
| Group | 168 | 3.75 | .001 | 43.30_4_ | <.001 | 106 | 1.13 | .392 | 4.56_2_ | .102 |
| Time (linear) | 297 | -3.96 | <.001 |  |  | 257 | -4.57 | <.001 |  |  |
| Time (quadratic) | 302 | 1.65 | .110 |  |  | - | - | - |  |  |
| Time (polynomial) | 305 | -0.68 | .500 |  |  | - | - | - |  |  |
| Time x Group | 306 | 2.62 | .060 |  |  | 258 | -0.59 | .697 |  |  |
| Time^2^ x Group | 308 | -2.62 | .060 |  |  | - | - | - |  |  |
| Time^3^ x Group | 309 | 2.50 | .060 |  |  | - | - | - |  |  |
| **Positive and Negative Syndrome Scale – Negative** | | | | | | | | | | |
| Group | 145 | 0.77 | .444 | 9.50_2_ | <.009 | 106 | 0.10 | .921 | 5.07_2_ | .079 |
| Time (linear) | 302 | -3.41 | .002 |  |  | 258 | -3.67 | <.001 |  |  |
| Time (quadratic) | 306 | 1.94 | .074 |  |  | - | - | - |  |  |
| Time (polynomial) | - | - | - |  |  | - | - | - |  |  |
| Time x Group | 307 | -0.02 | .991 |  |  | 259 | -0.05 | .962 |  |  |
| Time^2^ x Group | 309 | 0.06 | .991 |  |  | - | - | - |  |  |
| Time^3^ x Group | - | - | - |  |  | - | - | - |  |  |
| **Beck’s Depression Inventory** – **II** | | | | | | | | | | |
| Group | 122 | 1.15 | .303 | 8.31_2_ | .016 | 198 | -1.29 | .293 | 29.41_4_ | <.001 |
| Time (linear) | 219 | -2.98 | .006 |  |  | 131 | 1.46 | .250 |  |  |
| Time (quadratic) | 219 | 1.72 | .110 |  |  | 193 | -0.54 | .588 |  |  |
| Time (polynomial) | - | - | - |  |  | 193 | 1.07 | .320 |  |  |
| Time x Group | 226 | -0.01 | .991 |  |  | 195 | -4.46 | <.001 |  |  |
| Time^2^ x Group | 227 | 0.31 | .991 |  |  | 199 | 3.89 | <.001 |  |  |
| Time^3^ x Group | - | - | - |  |  | 199 | -3.35 | .003 |  |  |
| **Global Assessment of Functioning – functional disability** | | | | | | | | | | |
| Group | 158 | -3.15 | .006 | 23.77_2_ | <.001 | 159 | -1.80 | .220 | 15.35_2_ | <.001 |
| Time (linear) | 308 | 6.00 | <.001 |  |  | 279 | 4.32 | <.001 |  |  |
| Time (quadratic) | 313 | -3.37 | .002 |  |  | 283 | -2.38 | .030 |  |  |
| Time (polynomial) | - | - | - |  |  | - | - | - |  |  |
| Time x Group | 313 | -0.44 | .991 |  |  | 266 | 1.34 | .362 |  |  |
| Time^2^ x Group | 315 | 0.25 | .991 |  |  | 268 | -1.36 | .362 |  |  |
| Time^3^ x Group | - | - | - |  |  | - | - | - |  |  |
| **Global Assessment of Functioning** – **Symptoms** | | | | | | | | | | |
| Group | 207 | -2.46 | .030 | 17.25_2_ | <.001 | 282 | 3.70 | .220 | 9.42_2_ | .009 |
| Time (linear) | 318 | 5.86 | <.001 |  |  | 168 | -2.02 | <.001 |  |  |
| Time (quadratic) | 327 | -3.08 | .004 |  |  | 286 | -1.95 | .075 |  |  |
| Time (polynomial) | - | - | - |  |  | - | - | - |  |  |
| Time x Group | 325 | -0.81 | .837 |  |  | 268 | 1.18 | .399 |  |  |
| Time^2^ x Group | 329 | 0.53 | .991 |  |  | 270 | -0.96 | .482 |  |  |
| Time^3^ x Group | - | - | - |  |  | - | - | - |  |  |

*Abbreviations:* LRT = Likelihood Ratio Tests

## sTable 11 Results of the clinical predictor for patients with and without cannabis use disorder separately

|  | **Cannabis use disorder fulfilled** | **No Cannabis use disorder fulfilled** | **Wilcoxon-test:**  **Z-statistic** | **Wilcoxon-test: p-value** |
| --- | --- | --- | --- | --- |
| **Patients with recent-onset psychosis** | | | | |
| **True positives** | 30 | 8 | - | - |
| **True negatives** | 16 | 26 | - | - |
| **False positives** | 9 | 4 | - | - |
| **False negatives** | 8 | 9 | - | - |
| **Sensitivity (min-max)** | 78.9 (69.4 – 81.6) | 50.0 (37.5 – 56.2) | 2.66 | .008 |
| **Specificity (min-max)** | 64.0 (56.0 – 66.7) | 86.7 (80.0 – 86.7) | -2.52 | .012 |
| **balanced accuracy** | 71.4 | 68.3 | - | - |
| **Patients at clinical high-risk for psychosis** | | | | |
| **True positives** | 8 | 7 | - | - |
| **True negatives** | 8 | 20 | - | - |
| **False positives** | 5 | 4 | - | - |
| **False negatives** | 9 | 12 | - | - |
| **Sensitivity (min-max)** | 47.1 (47.1 – 47.1) | 36.8 (36.8 – 42.1) | 2.77 | .006 |
| **Specificity (min-max)** | 61.5 (53.8 – 61.5) | 86.9 (83.3 – 83.3) | -2.77 | .006 |
| **balanced accuracy** | 54.3 | 61.9 | - | - |

*Abbreviations:* min/max = lowest/highest sensitivity/specificity across permutations at the outer cross-validation folds.

# 8.) Supplementary Figures

| **SUBSTANCE USE**  (dd-mm-yyyy) | **- -** | |
| --- | --- | --- |
| **Cannabis lifetime**  *(follow-up: since the last assessment)* | 0 = No 1 = Yes | Date of onset: Date of offset:  (ongoing: 66-66-6666) |
| **Cumulative number of months:** |  | |
| **Daily/weekly frequency of use (average) during the last 3 months**  *(follow-up: since the last visit)* | 1 = daily  2 = > 3 days a week 3 = <= 3 days a week 4 = less than weekly 5 = never |  |
| **Cannabis - cumulative frequency of use during the last 3 months**  *(follow-up: since the last visit)* | 1 = 1-5 times  2 = 6-10 times  3 = 11-15 times  4 = 16-20 times  5 = 21-30 times  6 = > 30 times  7 = not applicable |  |
| **Last consumption**  (dd-mm-yyyy) | **- -** | |
| **Other substances lifetime**  *(follow-up: since the last visit)* | 1 = hallucinogens  2 = cocaine  3 = amphetamine-type stimulants incl. MDMA  4 = inhalants  5 = opioids  6 = PCP or similar type  7 = other designer drugs  8 = sedative-hypnotic- anxiolytic  9 = none |  |
| **Other substances – daily/weekly frequency of use (average) during the last 3 months**  *(follow-up: since the last visit)* | 1 = daily  2 = > 3 days a week 3 = <= 3 days a week 4 = less than weekly 5 = never |  |
| **Other substances - cumulative frequency of use during the last 3 months (different drugs can be added)**  *(follow-up: since the last visit)* | 1 = 1-5 times  2 = 6-10 times  3 = 11-15 times  4 = 16-20 times  5 = 21-30 times  6 = > 30 times  7 = not applicable |  |
| **Last consumption**  (dd-mm-yyyy) | **- -** | |

## sFigure 1 Substance Use Questionnaire

*Abbreviations:* MDMA = methylenedioxy-N-methylamphetamine, PCP = phencyclidine

##
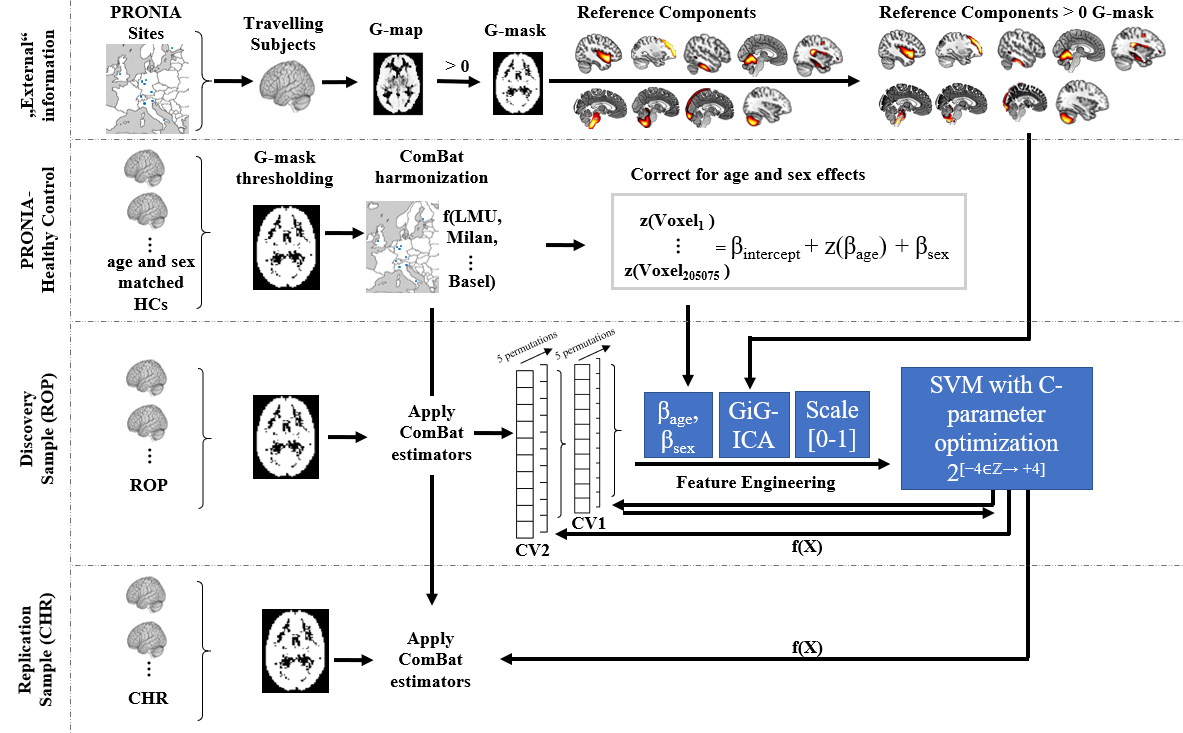
sFigure 2 Machine learning pipeline of structural Magnetic Resonance Imaging (sMRI)

In our machine learning pipeline, we used “external” information to account for site-specific scanner differences. Six subjects were scanned at six of the seven PRONIA sites, and their images were used to build a g-theory mask. This mask was used to exclude all voxels that were only associated with site-specific differences but showed no subject specific variation from the reference components (RCs) and all subjects. Then, the ComBat-algorithm was used to adjust for site specific-effects in a group of matched healthy individuals (HC), and the estimators were then used to remove site-specific effects independently from each ROP and CHR patient. Additionally, sex- and age- specific effects were regressed linearly from HC-sMRI images and the betas were used to remove these effects from independently from ROP and CHR patients in the machine learning pipeline. Finally, we trained and tested our machine learning model in ROP and applied the model to the completely held-out CHR. *Abbreviations:* CHR = clinical high-risk for psychosis, ROP = recent-onset psychosis, GiG-ICA = group information guided–independent component analysis, SVM = support vector machine.


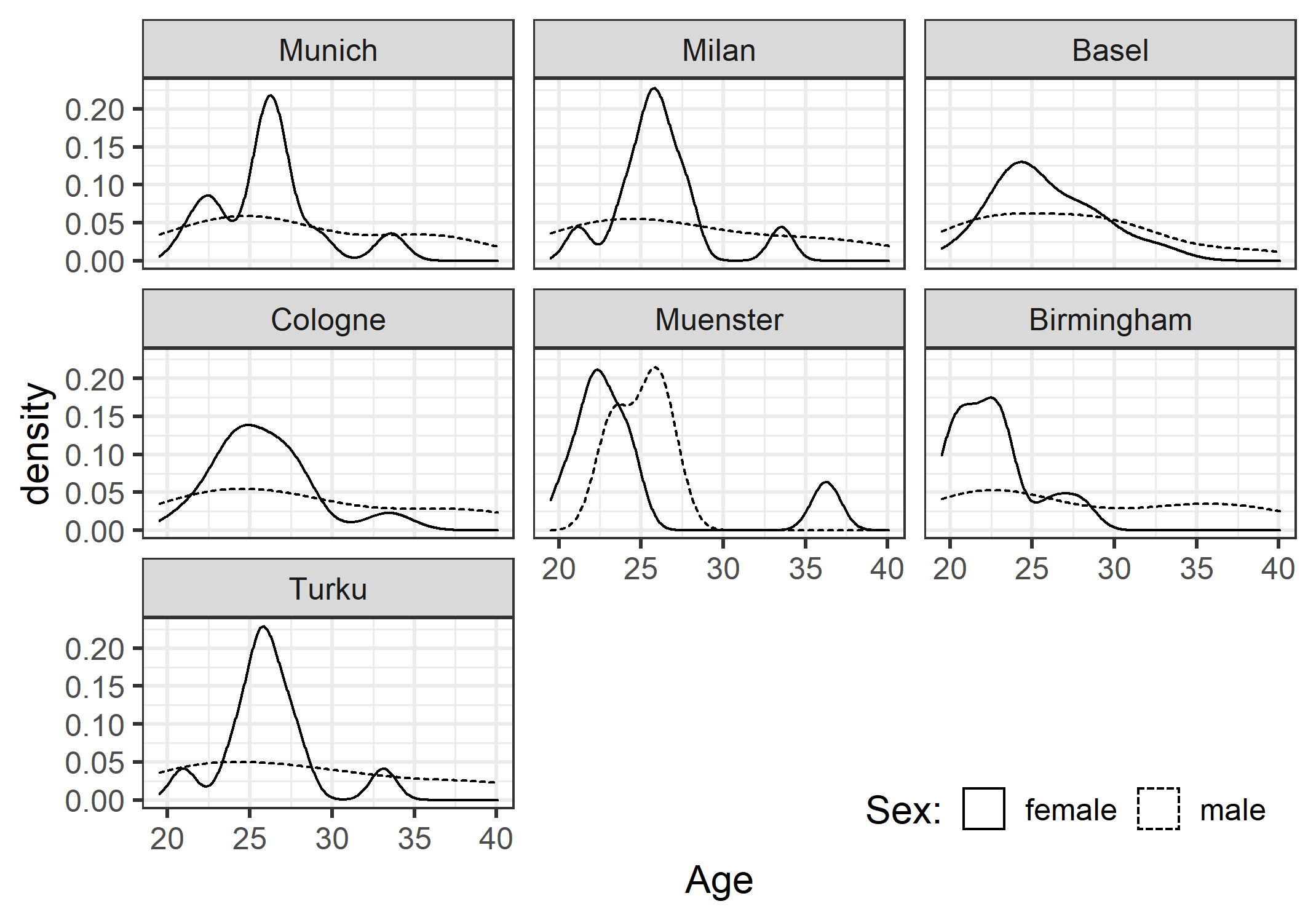


## sFigure 3 Age and sex distribution of healthy individuals used for harmonization between sites and regression of age- and sex specific effects


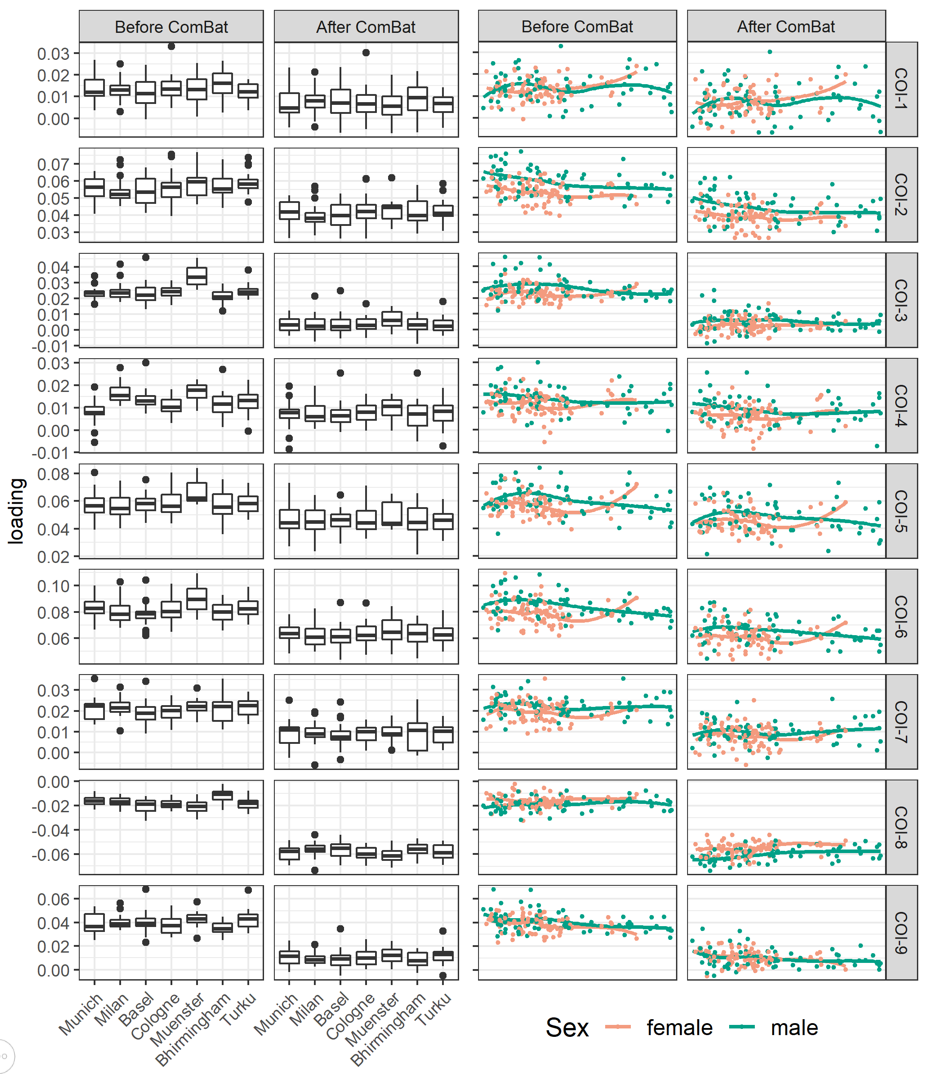


## sFigure 4 Components derived by group information-guided independent component analysis in healthy individuals used for harmonization between sites and regression of age- and sex specific effects before and after ComBat-harmonization

Differences between sites (left). Associations between loadings and age and sex (right). *Abbreviations:* COI = component of interest


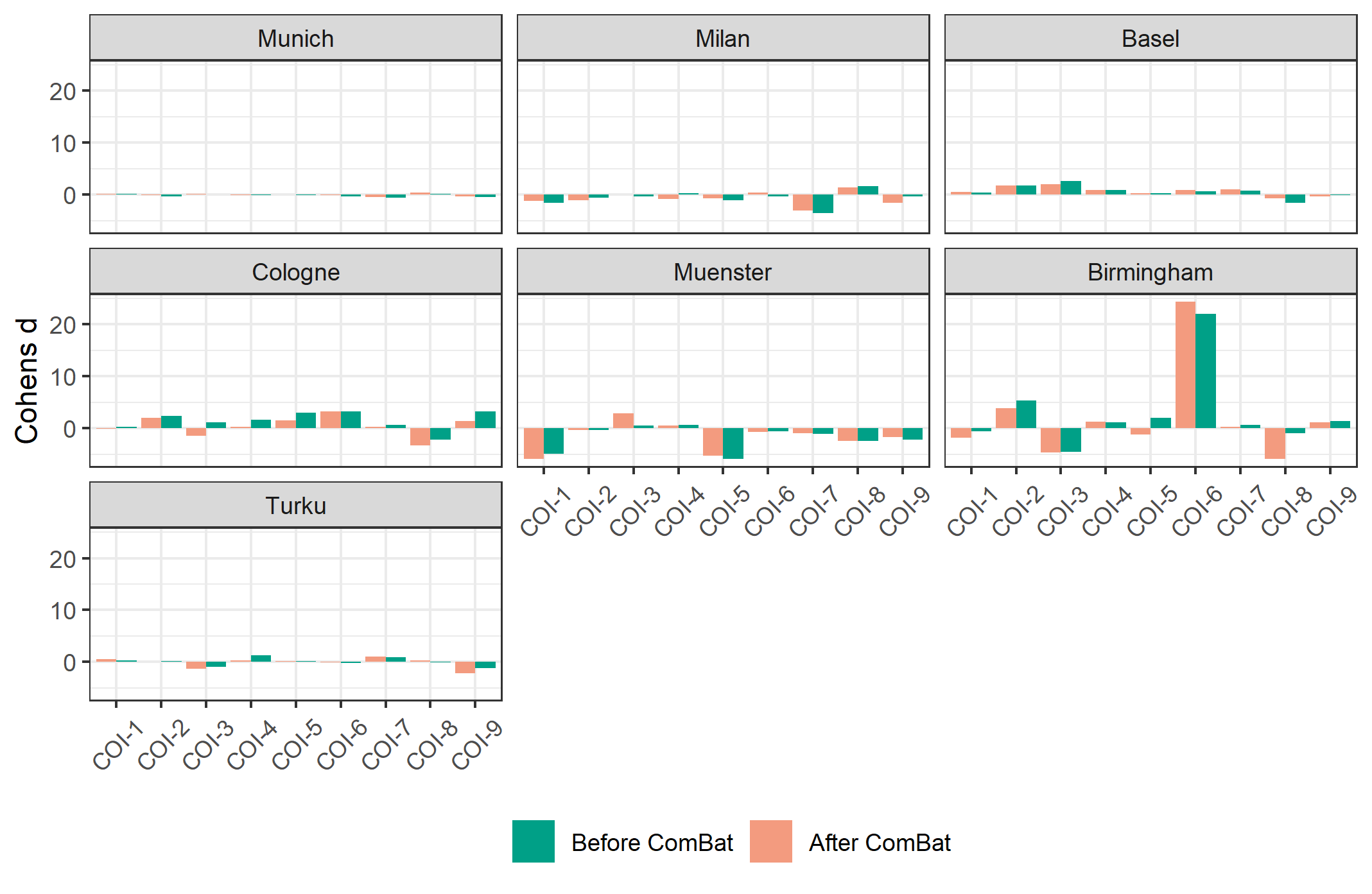


## sFigure 5 Effect sizes between patients with continued/discontinued cannabis use in recent-onset psychosis before and after harmonization

The effect sizes (Cohen’s d) between patients with continued cannabis use and discontinued cannabis use before and after harmonization (ComBat) for each site and each component of interest in recent-onset psychosis. *Note that the effect sizes are maintained. *Abbreviations:* COI = component of interest.


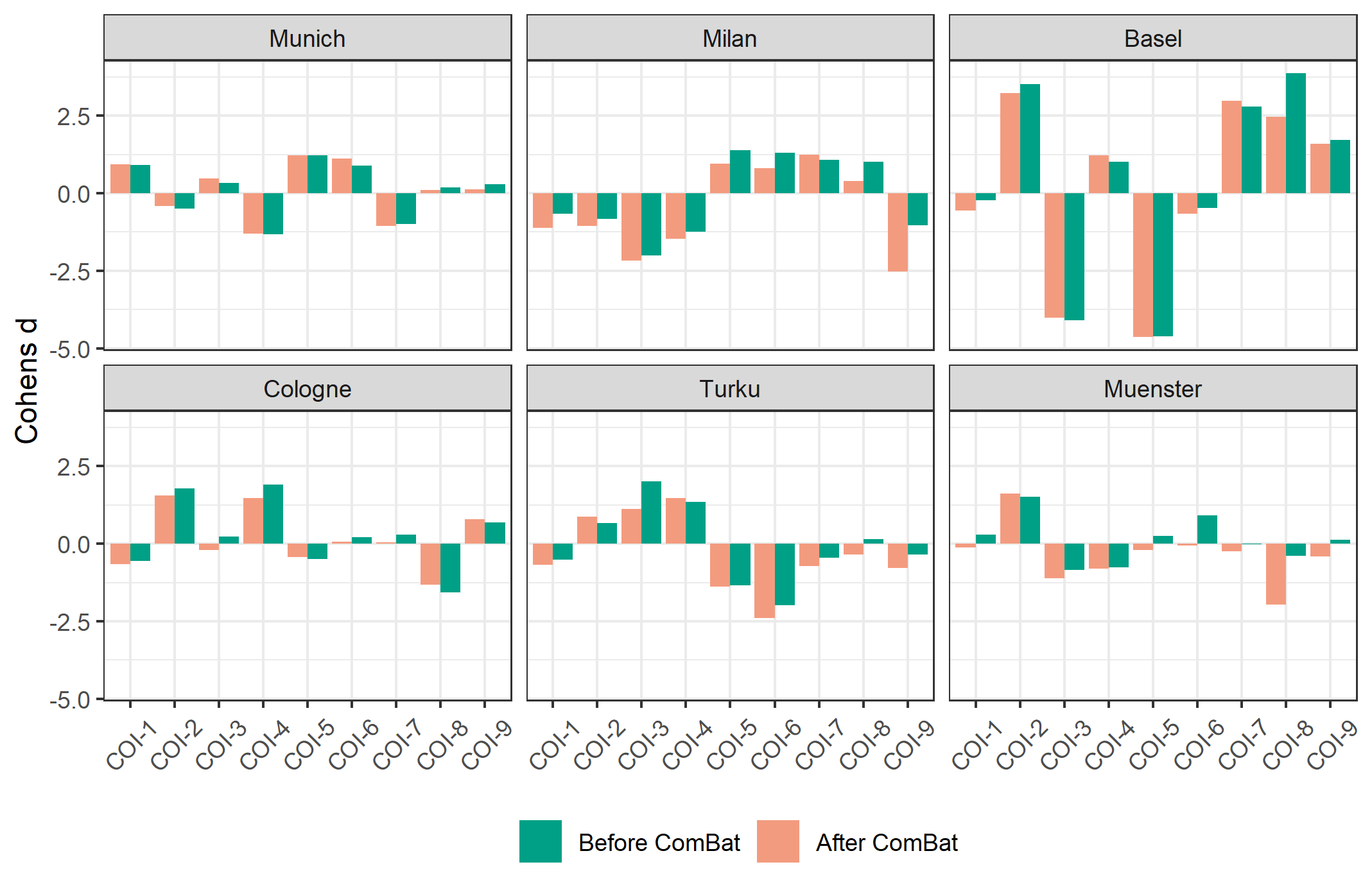


## sFigure 6 Effect sizes between patients with continued/discontinued cannabis use in clinical high-risk for psychosis before and after harmonization

The effect sizes (Cohen’s d) between patients with continued cannabis use and discontinued cannabis use before and after harmonization (ComBat) for each site and each component of interest in clinical high-risk for psychosis. *From Birmingham only subjects with discontinued cannabis use were included, thus no effect sizes could be calculated. Note that the effect sizes are maintained. *Abbreviations:* COI = component of interest.


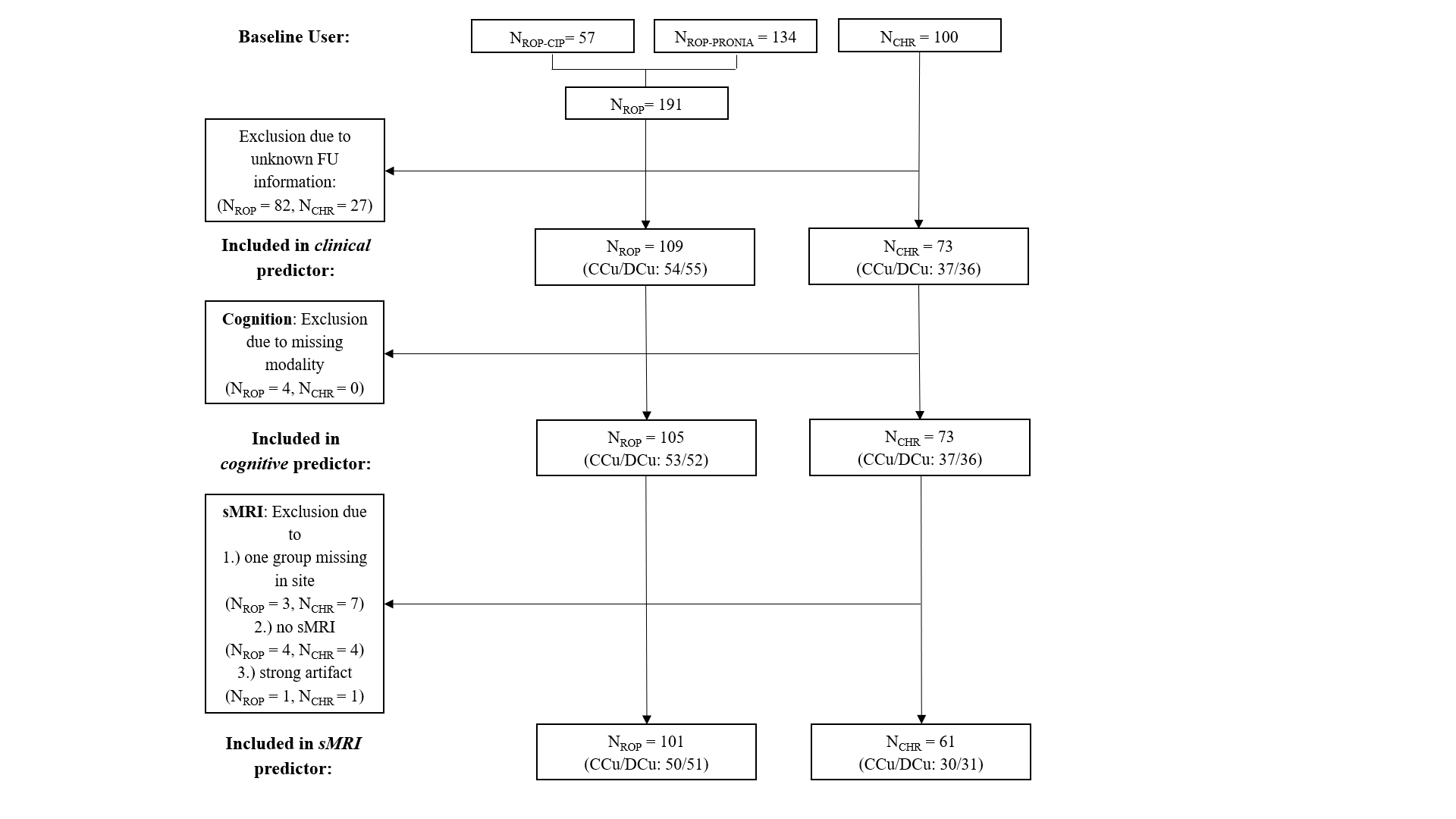


## sFigure 7 Flow Diagram of inclusion/exclusion for the three different predictors

*Abbreviations:* ROP = recent-onset psychosis, ROP-CIP = recent-onset psychosis patients included via the cannabis induced psychosis study, ROP-PRONIA = recent-onset psychosis patients included via the Personalized Prognostic Tools for Early Psychosis Management study, CHR = clinical high-risk for psychosis, sMRI = structural magnetic resonance imaging, CCu = continued cannabis use, DCu = discontinued cannabis use


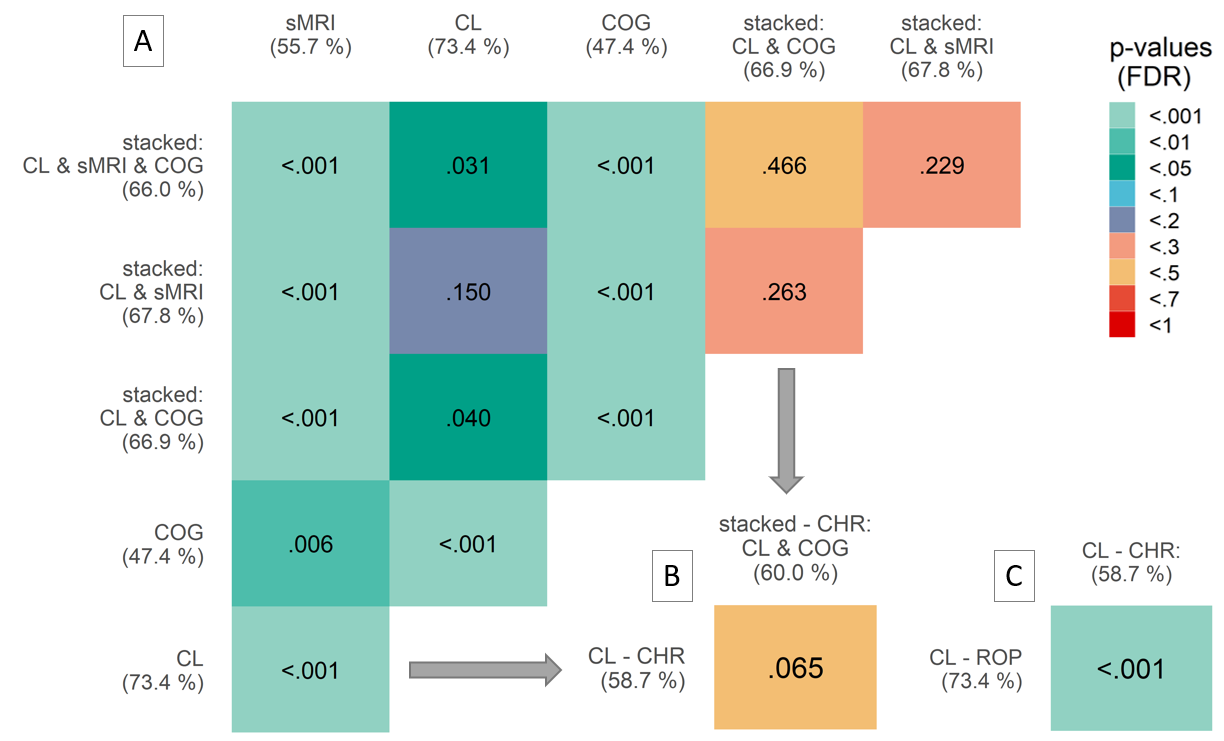


## sFigure 8 Comparison between different predictor performances

We compared the predictors’ median balanced accuracy (BAC) across all outer cross-validation (CV2) folds test data partitions between all unimodal and multimodal predictors (A). As we found a significant difference between all discovery models in our omnibus test (F_5, 245_ = 7.9, p < .001), we calculated pairwise comparisons between all predictors using the t-distribution approximation. P-values were false discovery rate (FDR) corrected. Further, we compared the median BAC from our replication data using the Wilcoxon test between our best performing uni- and multimodal predictors (B). Finally, we compared the best predictor (clinical) between the replication (recent-onset psychosis) and the discovery sample (clinical high-risk for psychosis) using the unpaired Wilcoxon test based on the respective median BACs across all CV2-folds (C). Numbers in the rectangles are respective p_FDR_-values between the two corresponding tests. Numbers in brackets indicate the BAC of the respective predictor. *Abbreviations:* sMRI = structural magnetic resonance imaging predictor, CL = clinical predictor, COG = cognitive predictor, ROP = recent-onset psychosis, CHR = clinical high-risk for psychosis, FDR = false discovery rate.

References

1. Gupta, C. N. *et al.* Patterns of Gray Matter Abnormalities in Schizophrenia Based on an International Mega-analysis. *Schizophrenia bulletin* **41,** 1133–1142; 10.1093/schbul/sbu177 (2015).

2. Penzel, N. *et al.* Association between age of cannabis initiation and gray matter covariance networks in recent onset psychosis. *Neuropsychopharmacology : official publication of the American College of Neuropsychopharmacology* **46,** 1484–1493; 10.1038/s41386-021-00977-9 (2021).

3. Koutsouleris, N. *et al.* Multimodal Machine Learning Workflows for Prediction of Psychosis in Patients With Clinical High-Risk Syndromes and Recent-Onset Depression. *JAMA psychiatry* **78,** 195–209; 10.1001/jamapsychiatry.2020.3604 (2021).

4. Koutsouleris, N. *et al.* Prediction Models of Functional Outcomes for Individuals in the Clinical High-Risk State for Psychosis or With Recent-Onset Depression: A Multimodal, Multisite Machine Learning Analysis. *JAMA psychiatry* **75,** 1156–1172; 10.1001/jamapsychiatry.2018.2165 (2018).

5. Koutsouleris, N. *et al.* Individualized Prediction of Functional Outcomes in Mental Healthcare: A Multi-modal, Multi-Site Machine Learning Analysis in the Clinical High-Risk State for Psychosis and Recent-Onset Depression. Supplementary Methods and Results. *JAMA psychiatry* **75,** 1–20 (2018).

6. Mushquash, C. & O’Conner, B. P. SPSS and SAS programs for generalizability theory analyses. *Behavior research methods* **38 (3),** 542–547 (2006).

7. Fortin, J.-P. *et al.* Harmonization of cortical thickness measurements across scanners and sites. *NeuroImage* **167,** 104–120; 10.1016/j.neuroimage.2017.11.024 (2018).

8. Chang, C.-C. & Lin, C.-J. LIBSVM. *ACM Trans. Intell. Syst. Technol.* **2,** 1–27; 10.1145/1961189.1961199 (2011).

9. Antonucci, L. A. *et al.* Multivariate classification of schizophrenia and its familial risk based on load-dependent attentional control brain functional connectivity. *Neuropsychopharmacology : official publication of the American College of Neuropsychopharmacology* **45,** 613–621; 10.1038/s41386-019-0532-3 (2020).

10. Schultze-Lutter, F., Addington, J., Ruhrmann, S. & Klosterkötter, J. *Schizophrenia Proneness Instrument. Adult version (SPI-A)* (Giovanni Fioriti, Rome, 2007).

11. McGlashan, T., Walsh, B. & Woods, S. *The psychosis-risk syndrome. Handbook for diagnosis and follow-up.* (Oxford University Press, New York, NY, 2010).

12. Yung, A. R. *et al.* *CAARMS. Comprehensive assessment at risk mental states.* (Parkville Victoria: The PACE Clinic, ORYGEN Research Centre, University of Melbourne, Department of Psychiatry, 2006).

13. Startup, M., Jackson, M. C. & Bendix, S. The concurrent validity of the Global Assessment of Functioning (GAF). *The British Journal of Psychological Society,* 417–422 (2002).

14. Spitzer, M. B., Gibbon, Robert, M., Gibbons, W., Janet, B. W. & Gibbon, Miriam, R. L., Williams, J. B. W. Structured Clinical Interview for DSM-IV-TR Axis I Disorders, Research Version, Non-Patient Edition.(SCID-I/NP). (2002).

15. Alvarez, E. *et al.* Premorbid Adjustment Scale as a Prognostic Predictor for Schizophrenia. *The British Journal of Psychiatry* **1987**.

16. Kay, S. R., Fiszbein, A. & Opler, L. A. The Positive and Negative Syndrome Scale (PANSS) for Schizophrenia. *Schizophrenia bulletin* **13,** 261–276; 10.1093/schbul/13.2.261 (1987).

17. Andreasen, N. The Scale for the Assessment of Negative Symptoms (SANS): Conceptual and Theoretical Foundations. *british journal of psychiatry* **1989,** 49–52.

18. Llorca, P.-M. *et al.* The "Functional Remission of General Schizophrenia" (FROGS) scale. Development and validation of a new questionnaire. *Schizophrenia research* **113,** 218–225; 10.1016/j.schres.2009.04.029 (2009).

19. Cornblatt, B. A. *et al.* Preliminary findings for two new measures of social and role functioning in the prodromal phase of schizophrenia. *Schizophrenia bulletin* **33,** 688–702; 10.1093/schbul/sbm029 (2007).

20. Zimet, G. D., Powell, S. S., Farley, G. K., Werkman, S. & Berkoff, K. A. Psychometric characteristics of the Multidimensional Scale of Perceived Social Support. *Journal of personality assessment* **55,** 610–617; 10.1080/00223891.1990.9674095 (1990).

21. Friborg, O., Hjemdal, O., Rosenvinge, J. H. & Martinussen, M. A new rating scale for adult resilience: what are the central protective resources behind healthy adjustment? *International journal of methods in psychiatric research* **12,** 65–76 (2003).

22. Endler, N. S. & Parker, J. D. Multidimensional assessment of coping. A critical evaluation. *Journal of Personality and Social Psychology* **58,** 844–854; 10.1037//0022-3514.58.5.844 (1990).

23. Connor, K. M. *et al.* Psychometric properties of the Social Phobia Inventory (SPIN). New self-rating scale. *british journal of psychiatry* **176,** 379–386 (2000).

24. Beck, A. T., Steer, R. A. & Brown, G. K. Beck depression inventory-II. San Antonio, 78(2), 490-8. *San Antonio* **78,** 490–498 (1996).

25. WHO. WHOQOL-BREF. Introduction, Administration, Scoring and Generic Version of the Assessment. *WHO Division of Mental Health* **1996**.

26. Veale, J. F. Edinburgh Handedness Inventory - Short Form. A revised version based on confirmatory factor analysis. *Laterality* **19,** 164–177; 10.1080/1357650X.2013.783045 (2014).

27. Cole, J. D. & Kazarian, S. S. The level of expressed emotion scale: A new measure of expressed emotion. *Journal of Clinical Psychology* **44,** 392–397 (1988).

28. Klein, M. H. *et al.* The Wisconsin Personality Disorders Inventory:. Development, Reliability and Validity. *Journal of Personality Disorders* **7(4),** 285–303 (1993).

29. David R. Williams, Yan Yu, James S. Jackson & and Norman B. Anderson. Racial Differences in Physical and Mental Health.

30. Haidl, T. K. *et al.* Validation of the Bullying Scale for Adults - Results of the PRONIA-study. *Journal of psychiatric research* **129,** 88–97; 10.1016/j.jpsychires.2020.04.004 (2020).

31. Bernstein, D. P., Fink, L., Handelsman, L. & Foote, J. *Childhood Trauma Questionnaire. Assessment of family violence: A handbook for researchers and practitioners.* (APA PsycTests, 1998).

32. Costa, P. T. & McCrae, R. R. *The NEO Inventories* (Routledge/Taylor & Francis Group., 2008).

33. Cornblatt, B. A., Risch, N. J., Faris, G., Friedman, D. & Erlenmeyer-Kimling, L. The Continuous Performance Test, Identical Paris Version (CPT-IP): I. New Findings About Sustained Attention in Normal Families. *Psychiatry research* **26,** 223–238 (1988).

34. Nowicki, S. & Duke, M. P. Individual differences in the nonverbal communication of affect. The diagnostic analysis of nonverbal accuracy scale. *J Nonverbal Behav* **18,** 9–35; 10.1007/BF02169077 (1994).

35. Schmidt, M. *Rey auditory verbal learning test:. A handbook* (CA: Western Psychological Services, Los Angeles, 1996).

36. Rey, A. L'examen Psychologique Dans les cas D'encephalopathie Traumatique (Les Problems),. *Archives de Psychologie,* 215–285 (1941).

37. Roiser, J. P., Stephan, K. E., den Ouden, H. E. M., Friston, K. J. & Joyce, E. M. Adaptive and aberrant reward prediction signals in the human brain. *NeuroImage* **50,** 657–664; 10.1016/j.neuroimage.2009.11.075 (2010).

38. Petrides, M. & Milner, B. Deficits on subject-ordered tasks after frontal- and temporal-lobe lesions in man. *Neuropsychologia* **20,** 249–262 (1982).

39. Reitan, R. M. *Trail Making Test:. Manual for administration and scoring.* (1992).

40. Wechsler, D. *WAIS-3., WMS-3: Wechsler adult intelligence scale, Wechsler memory scale: Technical manual.* (1997).
